# Analysis of ligand recognition by choline O-acetyltransferase reveals thiol-reactive assay interference and weak ligand affinity in solution

**DOI:** 10.1016/j.jbc.2026.113115

**Published:** 2026-05-07

**Authors:** Nina Forsgren, Frida Jonsson, Marcus Carlsson, Robin Afshin Sander, Andreas Larsson, Pernilla Lindén, Anna Linusson, Cecilia Springer Engdahl, Daniel Wiktelius, Fredrik Ekström

**Affiliations:** 1 CBRN Defence and Security, Swedish Defence Research Agency, Umeå, Sweden; 2 Department of Chemistry, Umeå University, Umeå, Sweden

**Keywords:** choline O-acetyltransferase (ChAT), carnitine O-acetyltransferase (CrAT), CoA mimicry, structural biology, surface plasmon resonance (SPR), biochemical assay interference, cysteine reactivity, X-ray crystallography

## Abstract

Choline O-acetyltransferase (ChAT) catalyzes the biosynthesis of acetylcholine and is a cysteine-rich enzyme that has been investigated using a range of biochemical, biophysical and structural approaches. Existing ChAT ligands rely on electrophilic or unstable scaffolds that limit their suitability for biological systems. Prior work established that arylvinylpyridiniums (AVPs) are substrate mimics that undergo ChAT-catalyzed hydrothiolation with CoA to form covalent AVP-CoA adducts. Here, we applied a structure-guided strategy to design nonreactive ligands intended to mimic key features of the AVP-CoA binding pose while avoiding covalent reactivity. Nineteen analogs were synthesized and evaluated across complementary biochemical, structural, and biophysical assays. X-ray crystallography confirmed that the new ligands bind within the ChAT tunnel similar to AVP-CoAs. Importantly, the high cysteine content of ChAT, especially within a reactive CXCXXC motif, rendered the enzyme susceptible to modification by the widely used 7-diethylamino-3-(4′-maleimidylphenyl)-4-methylcoumarin (CPM) reagent used for measuring ChAT activity, leading to confounding results in thiol-dependent activity assays. Enzyme-free counter-screens demonstrated that all apparent inhibitory activity arose from interference with assay readout rather than true enzymatic inhibition. Surface plasmon resonance measurements established that none of the designed ligands display detectable reversible affinity for ChAT, despite their crystallographically validated poses, and no selectivity over the related enzyme carnitine O-acetyltransferase (CrAT) was observed. These findings demonstrate that confirmed binding with X-ray crystallography is insufficient to establish functional interaction with ChAT and highlight the susceptibility of this enzyme to thiol-reactive assay artefacts. More broadly, this work underscores the necessity of integrating orthogonal biophysical validation when studying ligand binding to cysteine-rich enzymes.

The choline/carnitine O-acyltransferase enzyme family catalyzes the reversible transfer of acyl or acetyl groups between coenzyme A (CoA) and substrates. Within this family, choline O-acetyltransferase (ChAT) uniquely catalyzes the synthesis of the neurotransmitter acetylcholine from choline and acetylcoenzyme A (AcCoA), whereas the carnitine O-acyltransferases, a subgroup containing several enzymes, facilitate the transport and β-oxidation of fatty acids. These enzymes have also been proposed to play a role in maintaining the pool of AcCoA/CoA (1). The pivotal role of choline/carnitine-O-acyltransferases is manifested in genetic disorders, such as the potentially lethal congenital myasthenic syndrome disease that is linked to the gene expressing ChAT (2). The enzymes in the choline/carnitine-O-acyltransferases family are also potential targets for discovery of drugs for treatments of various conditions. For example, carnitine O-palmitoyltransferase (CPT1a, CPT1b, CPT1c, and CPT2) have been proposed as targets for anti-inflammatory therapeutics (3). Carnitine O-acetyltransferase (CrAT) and carnitine O-octanoyltransferase (CrOT) are potential therapeutic targets in metastatic melanoma (4), whereas ChAT is a proposed target for treatment of blood pressure disorders and intoxications caused by organophosphorus nerve agents (OPNAs) (5, 6).

OPNAs, such as sarin, VX, and A232 (Fig. 1*A*), function as covalent inhibitors of the essential enzyme acetylcholinesterase (AChE) (7). AChE terminates cholinergic signaling by a rapid hydrolysis of acetylcholine. Inhibition of AChE by OPNAs causes an accumulation of acetylcholine in the synapse, leading to a rapid and potentially lethal overstimulation of the nervous system. Intoxications caused by OPNAs can be treated with nucleophilic antidotes that attack the phosphorus atom of the OPNA-adduct, thereby liberating free reactivated AChE (8). One important limitation of currently available antidotes is their inadequate efficacy on certain OPNAs that form highly resistant adducts (9). Compounds inhibiting ChAT activity could potentially be used for symptomatic treatment of intoxications caused by OPNAs and thus is of interest for drug development. By limiting the production of acetylcholine, such drugs could mitigate the overstimulation of the nervous system, a hallmark of OPNA poisoning. This mechanism of action could complement existing nucleophilic antidotes, potentially providing a broad-spectrum solution effective across a wide variety of OPNAs. However, despite long-standing interest in choline/carnitine O-acyltransferase enzymes (10, 11), there is a notable absence of drug like inhibitors with a well-established inhibition mechanism. For example, we recently showed that the arylvinylpyridiniums (AVPs), such as the prototypical compound **1** (Fig. 1*B*), is not a reversible (nonreactive) inhibitor as originally believed, but instead participate in an *in situ* hydrothiolation reaction with CoA that results in the biologically active inhibitor **1-CoA** (Fig. 1*B*) (12). The *in situ* synthesis of **1-CoA** is due to a *thia*-Michael addition performed by the thiol of **CoA** on the 1,6-acceptor motif of **1** (*i.e.* a vinyl linker and an electron-withdrawing heteroarene).

**Figure 1:**
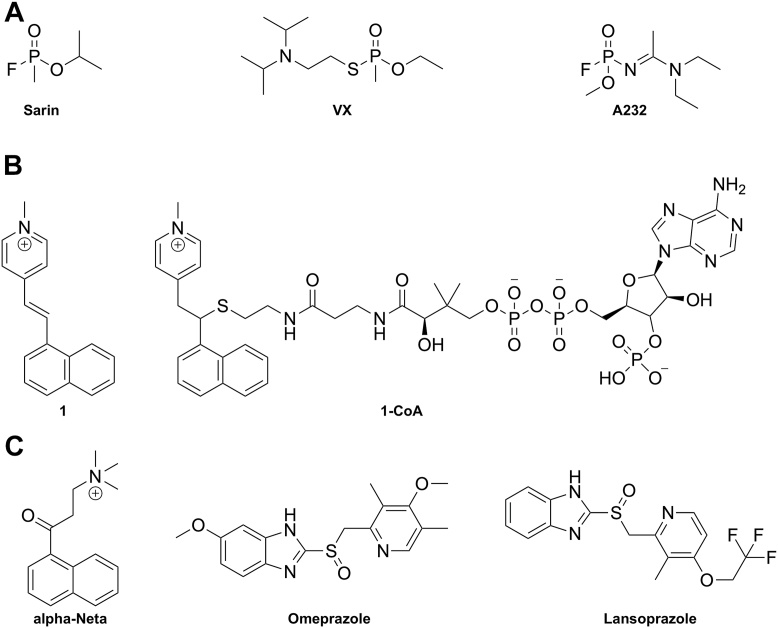
**Chemical structures of ligands that has been reported to inhibit AChE or ChAT.***A*, OPNAs that inhibit AChE with a covalent mechanism. *B*, the prototypical AVP **1** and the product of the ChAT catalyzed reaction product between **1** and CoA, **1-CoA**. *C*, previously reported chemically unstable inhibitors of ChAT. ChAT, choline O-acetyltransferase; AVP, arylvinylpyridinium; OPNA, organophosphorus nerve agent; AChE, acetylcholinesterase.

The reaction between AVPs and CoA offers unique and valuable possibilities for *in vitro* biochemical studies, as the resulting CoA analogs (AVP-CoAs) are reasonably potent and provide insights into enzyme behavior and inhibition. However, the AVPs utility *in vivo* is limited due to a combination of factors, including a confounding pharmacological profile (13, 14), a propensity of AVPs to photoisomerize (15), and the use of an electrophilic scaffold, which raises concerns about off-target toxicity. Furthermore, the corresponding AVP-CoA analogs are permanently charged molecules, which suggest they exhibit poor membrane permeability and low bioavailability (16). These limitations restrict AVPs and AVP-CoAs broader applicability despite their potential in controlled experimental environments. Proton pump inhibitors (PPIs) have also been reported to inhibit ChAT by a reversible mechanism (Fig. 1*C*) (17). However, for their molecular target, the gastric H,K-ATPase, PPIs are prodrugs that are activated by an rearrangement that occur *in vivo* before a covalent adduct is formed with target cysteine residues (18). PPIs are also known to bind a wide array of off-target proteins (19). For example, 20 and 25 experimentally confirmed targets have been identified for lansoprazole and omeprazole, respectively (20). Nonreactive CoA analogs have also been described as ChAT inhibitors (21), but their lack of selectivity toward CoA-utilizing enzymes renders them incompatible with more complex biological test systems.

Other reported inhibitors of ChAT include compounds identified by virtual screening (22), and aryl-3-oxopropanaminium compounds (*e.g.* alpha-Neta) (Fig. 1*C*) (23), which we have previously found to be chemically unstable (12). Thus, AVPs, PPIs, nonreactive CoA analogs, and aryl-3-oxopropanaminium compounds are unsuitable for cholinergic research and validation of ChAT as a drug target for treatment of OPNA intoxications.

In this study, we first investigate the selectivity of ChAT and the carnitine subgroup of enzymes by employing the *in situ* hydrothiolation reaction and a set of AVPs, which allowed us to assess the reactivity of the AVP scaffold with CoA bound to each enzyme. We subsequently focus on the design and synthesis of nonreactive ChAT ligands guided by structural information. Using the X-ray crystal structure of **1-CoA** and supporting computational modeling, we adapted the AVP scaffold by eliminating or deactivating the Michael acceptor functionality to generate reversible ligands with improved suitability for testing in biological systems. A focused series of analogs was prepared and evaluated in biochemical assays against ChAT, CrAT, and AChE. A few compounds formed the expected complexes, enabling structure determination by X-ray crystallography and initially displaying weak inhibitory activity. However, an enzyme free counter-screen later revealed these hits to be false positives arising from assay interference.

We also examined the inherent limitations of widely used biochemical assays for quantifying ChAT activity. We demonstrated that assays employing sulfhydryl-reactive detection reagents are intrinsically problematic, as highly reactive cysteine residues within ChAT readily form covalent adducts that compromise enzyme integrity and confound interpretation of biochemical data. Moreover, our results show that crystallographic complex formation alone cannot serve as a reliable orthogonal validation method in this system, since even false-positive compounds were able to bind within the active site under crystallographic conditions. To address this, we established surface plasmon resonance (SPR) as a suitable biophysical approach for confirming reversible ligand engagement. Together, these findings underscore the need for more robust assay systems capable of reliably measuring ChAT activity while minimizing nonspecific effects from reactive cysteine residues and other assay artifacts.

## Results

### Differential *in situ* hydrothiolation catalytic activity of ChAT and CrAT

**Figure 2:**
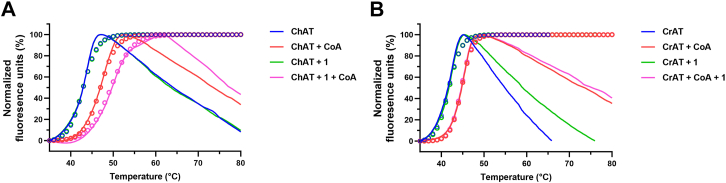
**Representative TSA curves.***A*, TSA for ChAT; (*B*) TSA for CrAT. The experimental data are shown as *solid lines* and the fit to the Boltzmann equation is indicated by *circles*. ChAT, choline O-acetyltransferase; CrAT, carnitine O-acetyltransferase; TSA, thermal shift assay.

The conserved 3D structures shared among ChAT/CrATs, along with their use of similar substrates and cofactors, motivated an investigation into the *in situ* hydrothiolation reactivity profiles of ChAT and CrAT using representative AVPs as molecular probes. We used a biophysical thermal shift assay (TSA), based on the thermal melting midpoint T_m_ of ChAT (T_m_^apo^) (24), to investigate the effect hydrothiolation products have on the enzymes. A selection of AVPs (**1**–**5**), in the presence or absence of CoA, were investigated (T_m_^sample^, Figs. 2*A* and S1). The effect of the compounds (or mixture of compounds) on the protein was calculated as a shift in melting temperature compared to apo (ΔT_m_ = T_m_^sample^ - T_m_^apo^). As the AVPs contain a Michael acceptor motif and function as co-substrates in an *in situ* hydrothiolation reaction with CoA, each compound was measured both without CoA (corresponding to a reversible complex as no *in situ* reaction can take place) and with CoA (corresponding to the stabilization provided by the AVP-CoA hydrothiolation adduct).

Using an assay concentration of 100 μM for the AVPs and CoA, we found a substantial stabilization of ChAT with a ΔT_m_ of 8.5 to 14.0 °C for the resulting hydrothiolation products of the AVPs and CoA reactions (*i.e.* CoA analogs, Table 1). As a comparison, binding of CoA alone (without any AVP) increased the T_m_ with 5.7 °C, while AVPs alone did not cause any significant stabilization of ChAT. These observations are in contrast to our studies of CrAT, where addition of CoA increased the T_m_ 2.6 °C and the addition of both CoA and AVPs resulted in a similar stabilization with ΔT_m_ of 2.5 to 2.8 °C (Fig. 2*B* and Table 1). Thus, our results indicate that the *in situ* hydrothiolation reaction does not occur in CrAT under these conditions or that the resulting CoA analogs do not provide any additional thermal stabilization of the protein.

**Table 1:** ΔT_m_ values for AVPs measured in ChAT and CrAT

Compound^b^		ChAT ΔT_m_ (°C)^a^				CrAT ΔT_m_ (°C)	
		-CoA		+CoA^c^		-CoA	+CoA
		TSA	CD^d^	TSA	CD	TSA	
	-			**5.7** (5.63–5.71)	**3.2**		**2.6** (2.55 to 2.70)
1		**0.0** (−0.13 to 0.13)	**−0.9**	**10.3** (9.95 to 10.72)	**14.6**	**0.0** (−0.20 to 0.27)	**2.8** (2.79 to 2.88)
2		**−0.6** (−0.69 to −0.55)	**0.2**	**14.0** (13.85 to 14.22)	**15.3**	**0.0** (−0.18 to 0.27)	**2.7** (2.64 to 2.79)
3		**0.2** (0.10 to 0.33)	**0.8**	**8.5** (8.23 to 8.74)	**9.8**	**0.2** (0.05 to 0.30)	**2.7** (2.60 to 2.78)
4		**0.1** (−0.05 to 0.25)	**0**	**9.1** (9.00 to 9.23)	**8.3**	**0.1** (0.05 to 0.20)	**2.6** (2.50 to 2.69)
5		**−0.5** (−0.54 to −0.49)	**−0.3**	**9.0** (9.01 to 9.10)	**8.1**	**0.1** (0.02 to 0.19)	**2.5** (2.35 to 2.59)

ChAT, choline O-acetyltransferase; AVP, arylvinylpyridinium; CrAT, carnitine O-acetyltransferase; TSA, thermal shift assay.

a Each sample was measured in four replicates, confidence intervals (95%) are shown in parenthesis.

b The concentration of the AVPs were 100 μM.

c The concentration of CoA was 100 μM.

d Data from CD measurements have been presented previously (12).

For ChAT, our results showed that changing the pyridinium *N*-substituent from methyl (**1**) to carbamoylmethyl (**2**) increased the thermal stability of the resulting AVP-CoA•ChAT complex by nearly 4 °C, while exchanging the naphthyl part of **1** to *3,4*-dichlorobenzyl- (**3** and **5**), or *3*-bromobenzyl moiety (**4**) reduced ΔT_m_ of resulting CoA analog-ChAT complex with at least 1.2 °C. We also note that the CoA analogs formed between CoA and the permanently charged compound **3,** and the neutral compound **5** in complex with ChAT had similar ΔT_m_ values. A comparison of the data obtained by TSA and previously reported data obtained *via* circular dichroism (CD) spectroscopy (12), shows that the TSA yielded results comparable to the CD assay, although a minor inconsistency was noted for compound **3**, which was ranked third in the CD assay and fifth in the TSA assay. Taken together, these findings indicate that CoA-dependent stabilization and thiol reactivity are markedly enzyme-specific within the choline/carnitine O-acyltransferase family.

### Analysis of the ***1-CoA***•*ChAT* complex and design strategy of nonreactive ligands

**Figure 3:**
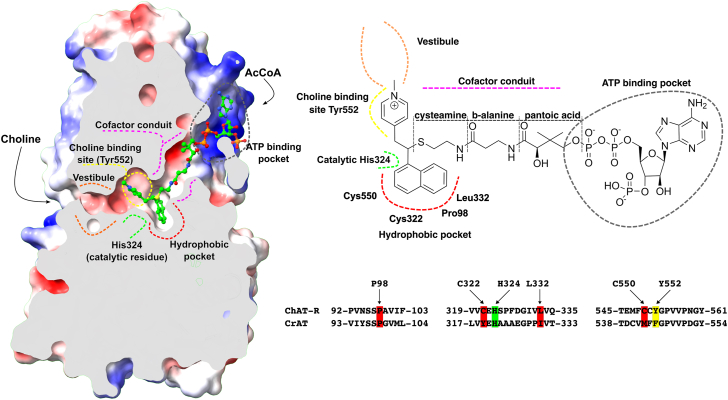
**X-ray crystal structure of a complex between 1-CoA and ChAT-SERM and a sequence alignment between ChAT and CrAT.** In the figure, **1-CoA** is shown as *green sticks*, ChAT is shown as a surface colored according to the electrostatic potential (*blue min*, *red max*) and sulfur, phosphorous, oxygen, and nitrogen are shown in *yellow*, *orange*, *red*, and *blue*, respectively. The sequence alignment between ChAT and CrAT highlights residues lining the hydrophobic pocket, the catalytic His and the choline binding arene in *red*, *green*, and *yellow*, respectively. ChAT, choline O-acetyltransferase; CrAT, carnitine O-acetyltransferase; ChAT-SERM, choline O-acetyltransferase surface-entropy-reduction mutant.

To support ligand design, we used the crystal structure of **1-CoA** bound to a reproducibly crystallizing surface-entropy-reduction mutant of ChAT (ChAT-SERM, Protein Data Bank (pdb) 7AMD) as a template (24). The choline/CrAT family features a bi-reactant tunnel spanning the protein, allowing CoA and the small-molecule substrate to enter from opposite sides (Fig. 3) (21, 25). In **1-CoA**•ChAT-SERM, the ATP portion of CoA binds at a shallow, solvated surface pocket (the “ATP pocket”), whereas the pantetheine chain extends through a ∼15 Å conduit toward the catalytic site. The hydrophobic pocket surrounding the bound naphthyl group differs between ChAT and the CrAT subgroup (Fig. 3), indicating a potential site for ChAT-selective interactions. The pyridinium ring engages Tyr552 in arene-arene contacts adjacent to the catalytic His324, and the N-methyl substituent of the pyridinium ring projects toward the choline entry vestibule at the opposite tunnel opening. In addition, the saturated linker in **1-CoA** allows the naphthyl and pyridinium moieties to adopt a noncoplanar orientation, clearly defined in electron density as the (R)-configured *thia*-Michael adduct.

**Figure 4:**
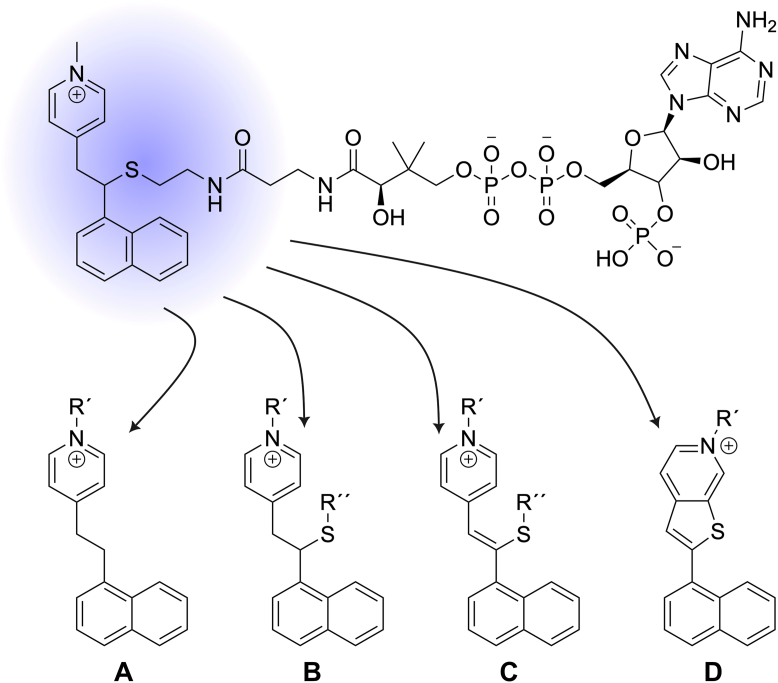
**Design of target molecules.***A*, the design was directed by the bioactive form of the AVP **1** (*i.e.* the *in situ* hydrothiolation product formed by a reaction with CoA). Four different types of compounds (*A–D*) were investigated. AVP, arylvinylpyridinium.

We designed a focused set of molecules by simplifying **1-CoA** while retaining key recognition elements (Fig. 4). Our first design (A) generated compounds that mimicked **1** but replaced the Michael acceptor motif with a fully saturated linker between the naphthyl and pyridinium moieties. Design B resulted in molecules resembling **1-CoA** but with parts of the linker and the entire ATP moiety omitted. Similar to compounds in design A, the linker is saturated, but now with short thioethers appendices on the linker between the naphthyl and the pyridinium ring, potentially enabling interactions with the cofactor conduit (design B, *cf.* Fig. 3). In design C, we designed compounds with the linker as vinyl thioether thereby retaining the molecular rigidity and electronic conjugation of AVPs. Finally, expanding on design C, in design D the vinyl-thioether linker was tied into the pyridine ring, giving a rigid thieno[3,2-c]pyridin-1-ium scaffold, which rendered molecules with significantly reduced structural flexibility. Docking studies showed that all designed compounds retained the naphthyl-pocket and pyridinium - Tyr552 interactions, with linker substituents oriented consistently toward the cofactor conduit. Stereochemical and geometric variants adopted comparable binding modes (Table S1 and S2). In total, 19 compounds were designed and synthesized (Table 2 and Table S2). Synthetic details are provided in Supp. Data S1 and Data S2. The compounds were subsequently evaluated for ChAT binding using a combination of biochemical, biophysical, and structural techniques.

**Table 2:** A selection of compounds synthesized for biochemical evaluation

Cpd no.	Type	Structure	Apparent IC_50_^a^^b^ (μM)			IC_50_^a^^b^ (μM)
			ChAT	CrAT	Counter screen	AChE
S-ethyl-CoA	Ref		1.6 (1.2 to 2.2)	168 (105 to 835)	>500	n.d.
**6**	A		>500	>500	>500	n.d.
**7**	B		>500	>500	>500	n.d.
**8**	B		>500	>500	n.d.	n.d.
**(Z)-9**	C		190 (150 to 250)	250 (180 to 340)	260 (70)^c^	9.5 (7.4 to 13)
**(E)-9**	C		380 (220 to 730)	n.d.	370 (120)	n.d.
**(Z)-10**	C		170 (150 to 210)	190 (140 to 390)	160 (90 to 15000)	0.09 (0.07 to 0.1)
**11**	D		>500	270 (120)^c^	570 (240)^c^	1.2 (1.0 to 1.3)

CrAT, carnitine O-acetyltransferase; AChE, acetylcholinesterase; ChAT, choline O-acetyltransferase; C.I., confidence interval.

a Additional compounds are shown in Table S2. Dose-response curves are shown in Fig. S6.

b Mean of at least three replicates. The 95% C.I. is shown in parenthesis.

c Only lower limit determined

#### Biochemical assays

**Figure 5:**
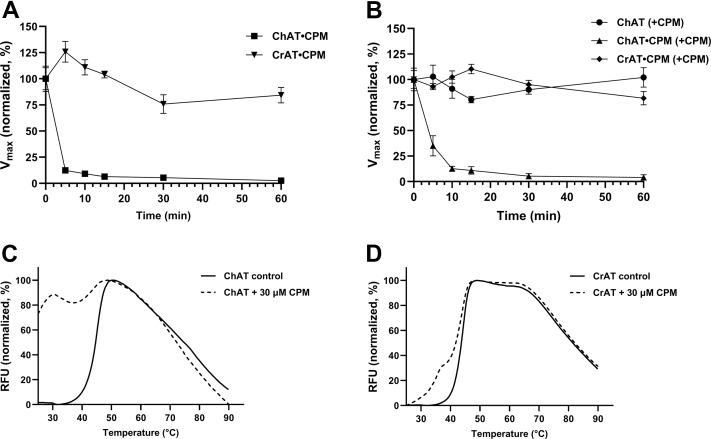
**Analysis of CPM’s influence on catalytic activity and thermal stability:***A*, residual activity of ChAT and CrAT after preincubation with 30 μM CPM for the indicated times. *B*, residual activity of ChAT and CrAT after the same preincubation, followed by a second 30 μM CPM addition immediately before activity measurement. *C*, TSA melt curve of ChAT in the presence or absence of CPM. *D*, TSA melt curve of CrAT under identical conditions. For *panels A* and *B*, bars represent the mean ± standard deviation of three independent technical replicates (n = 3); activities are expressed as a percentage of the corresponding untreated control. ChAT, choline O-acetyltransferase; CPM, 7-diethylamino-3-(4′-maleimidylphenyl)-4-methylcoumarin; CrAT, carnitine O-acetyltransferase; TSA, thermal shift assay.

7-Diethylamino-3-(4′-maleimidylphenyl)-4-methylcoumarin (CPM) based high-throughput fluorometric assay has been proposed for screening ChAT inhibitors (22). The thiol group of CoA, generated from the reaction between AcCoA and choline (in ChAT) or L-carnitine (in CrAT), reacts with CPM's maleimide moiety producing a fluorescent adduct, which has also been used in other CoA-generating assays (26, 27). In the ChAT study, the fluorescent signal was monitored 15 to 20 min, and the slope of the progress curve was related to the enzymatic activity (22). As both ChAT and CrAT have free thiols from cysteine residues, we designed experiments aimed to investigate potential reactions between CPM and cysteine residues. First, the proteins were incubated with CPM and the fluorescent progress curve was followed for 20 min. These experiments indicated an immediate reaction between CPM and ChAT, as the fluorescent signal increased over time, while CrAT appears less reactive with a progress curve that is similar to the control sample (Fig. S3). To determine whether CPM affects the catalytic activity, we preincubated the enzymes (40 nM) with 30 μM CPM at room temperature (0–60 min) and measured the activity post incubation (Fig. 5*A*). To limit the effect by side-reactions between CPM and cysteine residues, we restricted our activity measurement to 60 s. We observed a rapid decline in ChAT activity, with only ∼15% of the original activity remaining after 5 min. In contrast, CrAT exhibited greater resilience, retaining ∼85% activity after 60 min (Fig. 5*A*). Further experiments involved preincubating the enzymes with CPM, followed by the addition of a second portion of CPM immediately prior to activity measurement (Fig. 5*B*). ChAT displayed a consistent decline in activity, whereas CrAT remained largely unaffected by CPM preincubation. Notably, ChAT activity could not be restored by addition of fresh CPM immediately prior to measurement, indicating irreversible inactivation under these conditions. In contrast, a control ChAT sample that was not pretreated with CPM but otherwise handled identically retained full activity, behaving similarly to CrAT. This control demonstrates that ChAT maintains activity and that the observed loss of ChAT activity is specifically due to CPM preincubation rather than an inherent limitation of *e.g.* enzyme stability (Fig. 5*B*). To assess if CPM affects the stability of the proteins, we performed TSA in the presence of CPM. Control samples of ChAT and CrAT exhibited typical sigmoidal thermal melting profiles, indicative of well-folded proteins (Fig. 5, *C* and *D*). However, CPM addition markedly altered ChAT’s thermal melting curve, while CrAT’s profile remained comparable to the control. To investigate covalent addition of CPM to cysteine residues, we incubated ChAT and CrAT with CPM (at the same molar ratios used in the biochemical assays) for 2 minutes, and analyzed the proteins using tryptic cleavage followed by ultra-high performance liquid chromatography-high resolution mass spectrometry (UHPLC-HRMS). We found that CPM form covalent adducts with at least 10 (out of 20 possible) cysteine residues in ChAT (sequence coverage 63%, Fig. S4*A*), while we detected the CPM adduct at 3 (out of eight possible) cysteine residues in CrAT (sequence coverage 62%, Fig. S4*B*). For untreated control samples, the sequence coverage was 74 and 62% for ChAT and CrAT, respectively (Fig. S5). To conclude, CPM rapidly modifies multiple cysteine residues in ChAT, leading to time-dependent loss of catalytic activity while CrAT remains largely unaffected. TSA and UHPLC-HRMS analyses indicated that CPM disturbs ChAT’s structural integrity through covalent modification of cysteine thiols. Based on our results, we thus altered the assay conditions and limited the preincubation time to 1 min, resulting in a total assay duration of ∼2 min which ensured full enzymatic activity for ChAT during the experiments. In addition, we also included a counter screen as recommended by Baell and Walters for assays involving thiol-reactive detection reagents such as CPM, in order to detect false positives (28). A synthetic reference inhibitor, S-ethyl-CoA, was included as a positive control and showed robust and reproducible inhibition of ChAT and CrAT with half maximal inhibitory concentration (IC_50_) values of 1.6 μM and 168 μM for ChAT and CrAT, respectively (Table 2).

The IC_50_ of the compounds was determined from their dose-response against ChAT. We assessed the compounds’ selectivity by determining their inhibition of CrAT. A subset of molecules was also tested for inhibition of AChE. To measure the activity of AChE, we employed the well-established Ellman assay, which tracks the hydrolysis of acetylthiocholine into thiocholine (29). This hydrolysis reaction was monitored spectrophotometrically using the thiol-sensitive reagent DTNB (5,5′-dithio-bis-(2-nitrobenzoic acid). Some compounds showed precipitation at high concentrations in the CPM assay. To objectively identify where a precipitated compound material potentially interfered with the assay (potential false positive or potential false negative), we measured the absorbance at 700 nm following assay completion. Wells identified as cloudy in this absorbance measurement were indicated in the dataset. We found that both ChAT and CrAT were stable at 10% dimethyl sulfoxide (DMSO), which was included in the assay.

#### Biochemical evaluation

**Figure 6:**
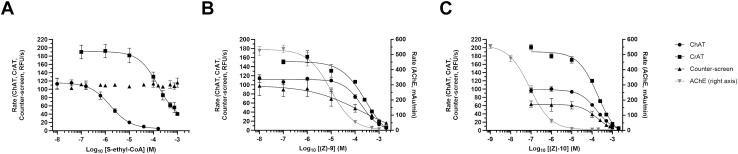
**Representative dose-response curves from biochemical assays and counter screen.***A*, dose response for S-ethyl-CoA; (*B*) **(Z)-9** and (*C*) **(Z)-10**. Bars represent the mean ± standard deviation of three independent technical replicates (n = 3).

We initially assessed the 19 designed compounds for inhibition of ChAT and CrAT using our modified version of the CPM-based biochemical assay, and a subset was also examined for AChE inhibition using the Ellman assay (29). A selection of chemical structures of analogs is shown in Table 2, with the remaining in Table S2; corresponding dose–response curves are provided in Figures 6 and S6.

Only a few compounds produced a dose-response within the accessible concentration range, while several compounds precipitated during testing. Among those initially appearing active, Group C derivatives produced the most pronounced effects. For example, analogs such as **(Z)-9**, **(E)-9,** and **(Z)-10** showed apparent IC_50_ values in the midmicromolar range, and some group D analogs appeared to inhibit CrAT preferentially. A notable feature of several dose–response curves was the presence of abnormally steep Hill slopes, often substantially greater than expected for a 1:1 binding stoichiometry. Curve shapes displaying Hill coefficients far exceeding unity are characteristic of nonspecific assay interference, including artefacts arising from compound aggregation or perturbation of detection chemistry.

In the enzyme-free counter-screen where compounds and all assay reagents were incubated in the absence of enzyme, AcCoA was replaced with CoA to allow fluorescence generation by CPM in the absence of ChAT. Notably, addition of increasing concentrations of the molecules resulted in dose-response relationships under enzyme-free conditions for the same compounds that gave sigmoidal inhibition curves in the presence of enzyme (Fig. 6). This shows that the CPM signal loss originates from compound interference of the fluorescent signal rather than ChAT or CrAT inhibition. Taken together, these findings demonstrate that none of the tested compounds inhibited ChAT or CrAT in the CPM assay. The apparent activity trends instead reflect assay interference and thus cannot inform ligand optimization (26).

#### SPR reveals reversible binding of CoA and S-ethyl-CoA

**Figure 7:**
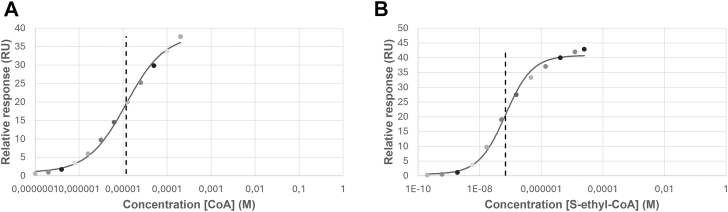
**SPR binding affinity of compound.***A*, CoA and (*B*) S-ethyl-CoA. Representative data. SPR, surface plasmon resonance.

To reliably assess ligand engagement with ChAT under solution conditions and to avoid the interference observed in CPM-based biochemical assays, we established an SPR assay using immobilized ChAT. SPR is a biophysical method that directly monitors binding interactions in real time and is well suited for quantifying low-affinity, reversible ligand interactions. As reference compounds, CoA and S-ethyl-CoA were included and found to generate clear, concentration-dependent responses yielding an apparent equilibrium dissociation constant (*K*_*D*_) of 12 μM for CoA and 69 nM for S-ethyl-CoA (Fig. 7 and Table S3). These results confirmed the robustness of the SPR assay and demonstrated that the immobilized ChAT maintains native ligand recognition.

In contrast, the compounds **1**, **(Z)-9**, **11**, **22**, **23,** and **24** displayed no concentration depended binding to ChAT (maximal tested concentration 600 μM), and kinetic fitting or steady-state analysis was not possible within the accessible solubility and concentration limits (Fig. S7). The combined lack of measurable inhibition in the biochemical assays and the absence of detectable affinity by SPR demonstrate that the ligands do not effectively compete for the ChAT active site under these conditions. These findings provided a critical benchmark for interpreting the subsequent structural studies and underscore the necessity of further optimization to achieve functionally relevant inhibition.

#### Crystallographic studies of the compounds binding to ChAT and CrAT

In parallel with the biochemical experiments, we also conducted crystallographic studies aiming for exploring ligand recognition by ChAT and CrAT. Crystals of choline O-acetyltransferase surface-entropy-reduction mutant (ChAT-SERM) and CrAT were soaked with compounds from the four chemotypes (groups A–D). Although most attempts did not give ligand-bound structures (ligand concentration exceeding 10 mM), we obtained ChAT-SERM complexes with compound **6** (group A, **6**•ChAT-SERM, 1.6 Å resolution, pdb entry code 9F85), compound **8** (group B, **8**•ChAT-SERM, 1.9 Å resolution, pdb entry code 9F84), and **(E)-9** (group C, **(E)-9**•ChAT-SERM, 1.8 Å resolution, pdb entry code 9RT3). For CrAT, a complex was solved with **11** from group D to a resolution of 1.4 Å (**11**•CrAT, pdb entry code 9SCK). Data collection and refinement statistics are given in Table S4. Electron density maps are shown in Figs. S8 and S9 for ChAT and CrAT, respectively.

**Figure 8:**
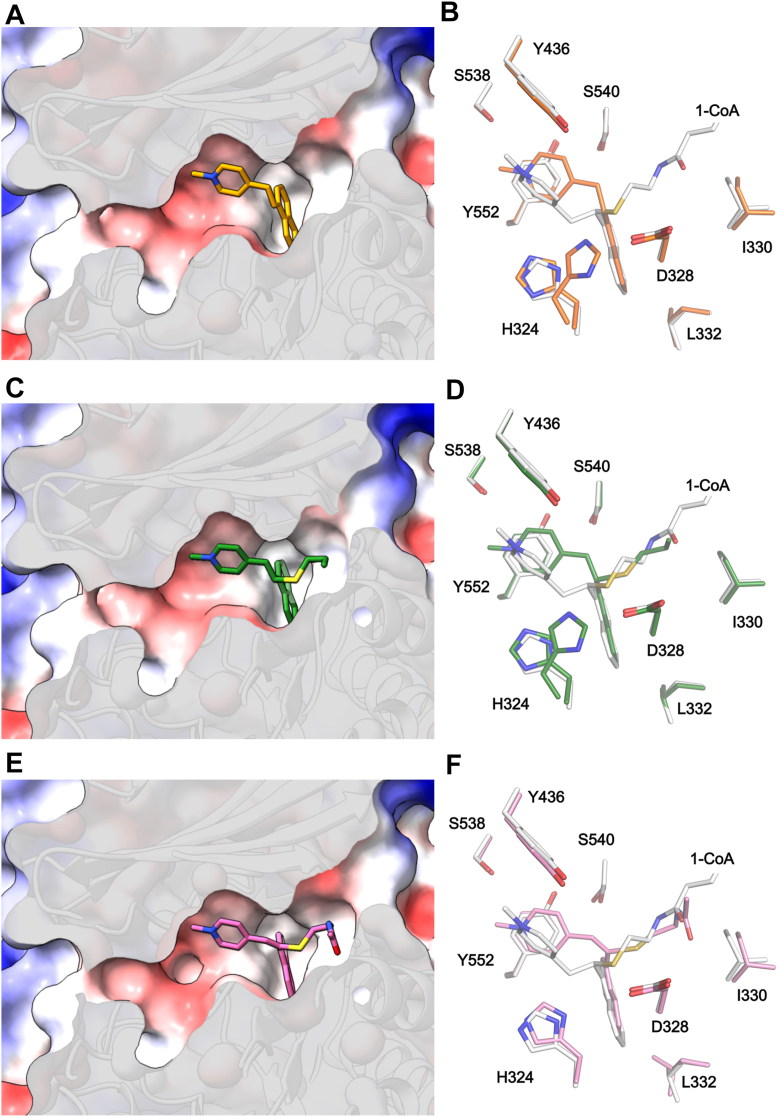
**X-ray crystal structures, these binding modes do not confer measurable potency.***A*, the binding of **6** in the active site tunnel of ChAT-SERM. *B*, superposition between **6**•ChAT-SERM and **1-CoA**•ChAT-SERM. *C,* binding of **8** to ChAT-SERM. *D*, superpositioning of **8**•ChAT-SERM and **1-CoA**•ChAT-SERM. *E*, binding of **(E)-9** to ChAT-SERM. *F*, superpositioning of **(E)-9**•ChAT-SERM and **1-CoA**•ChAT-SERM; *A*, *C*, and *E*, the surface is colored according to the electrostatic potential with compound **6** (*orange*), **8** (*green*), and **(E)-9***(pink)* shown as *sticks*. *B*, *D*, and *F***6**•ChAT-SERM, **8**•ChAT-SERM, **(E)-9**•ChAT-SERM and **1-CoA**•ChAT-SERM are shown in *orange*, *green*, *pink*, and *gray*, respectively. Oxygen, nitrogen, and sulfur are shown in *red*, *blue*, and *yellow*, respectively. ChAT, choline O-acetyltransferase; ChAT-SERM, choline O-acetyltransferase surface-entropy-reduction mutant.

Compounds **6**, **8,** and **(E)-9** bind in a similar fashion to ChAT (Figs. 8, *A*–*F* and S8), and agreed well with the predicted binding poses by molecular docking simulations and the structure of **1-CoA** (12). In all cases, the naphthyl ring is deeply buried in the hydrophobic pocket, the pyridinium ring is accommodated in the choline binding site, and each molecule’s aliphatic branch extends along the cofactor conduit. In **6**•ChAT-SERM, the saturated ethylene linker extends toward the choline binding site and the pyridinium of **6** form a nearly parallel face-to-face arene-arene interaction with the phenol ring of Tyr552 (Fig. 8, *A* and *B*). The N-methyl moiety is accommodated in a shallow pocket. In comparison with **1-CoA**•ChAT-SERM, the plane of the pyridinium is rotated nearly 80° in the structure of **6**•ChAT-SERM. Furthermore, the sidechain of the catalytic His324 is found in two distinct conformations, which may be induced by unfavorable close contacts between the linker and the imidazole group of His324. The **8**•ChAT-SERM (Fig. 8, *C* and *D*) and **(E)-9**•ChAT-SERM (Fig. 8, *E* and *F*) complexes are similar to **6**•ChAT-SERM, the naphthyl and pyridinium rings superimpose on those of **6**, the branch overlays the N-acetyl-cysteamine of **1-CoA** and the sulfur is close to the Gly329 backbone amide. The electron density map unambiguously shows that only the (*S*)-enantiomer of **8** binds to ChAT, contrasting with the (*R*)-enantiomer of **1-CoA** (Fig. S8). Compared with the apo structure of ChAT (apo ChAT-SERM, pdb entry code 2FY2), the structures display a change of side chain conformations of Cys322, Leu332, and Cys550 that is linked to the binding of the ligands’ naphthyl moiety in the hydrophobic pocket. Furthermore, a careful inspection of the electron density maps did not indicate any additional, potentially unspecific, low occupancy binding sites.

Compound **11** binds CrAT at the vestibule entrance, its thieno[3,2-c]pyridin-1-ium scaffold makes arene-arene contacts with Tyr89, while the benzyl group extends toward the catalytic His322 and forms edge-to-face interactions with Tyr86 (Fig. S9). This binding site is unique to CrAT, ChAT carries Asn88 in place of Tyr89, and so the same interactions cannot occur.

## Discussion

Reversible and selective small-molecule inhibitors of ChAT remain unavailable despite their importance as chemical probes and their potential role in OPNA countermeasure development. Existing chemotypes, including AVPs, PPIs, and aryl-3-oxopropanaminium compounds, suffer from electrophilic reactivity, instability, or broad off-target interactions, which limit their translational potential. In this work, we pursued a nonreactive ligand design strategy aimed at capturing the essential molecular recognition features of the **1-CoA** binding mode while eliminating the reactive motifs that have complicated previous efforts.

The structure-guided design proved conceptually sound. Docking simulations correctly predicted binding orientations that were later confirmed by X-ray crystallography. The naphthyl group of the compounds consistently occupied the hydrophobic pocket, the pyridinium ring formed arene-arene interactions with Tyr552, and the appendices of the linker interacted with the cofactor conduit. These observations demonstrate that the designed ligands can access the intended regions of the ChAT tunnel and reproduce key structural interactions of **1-CoA**. However, biophysical and biochemical measurements showed no detectable affinity within the accessible concentration range, demonstrating that these interactions were not sufficiently energetically favorable in solution. The crystallographic complexes therefore reflect low-affinity encounter states that can be trapped in the crystal lattice rather than indicative of productive binding under physiological conditions.

A comparable disconnect between well-defined binding poses and weak functional engagement has been widely reported in fragment-based and structure-based drug discovery. Fragment crystallography routinely reveals binding poses for ligands with affinities in the high-micromolar to millimolar range in biophysical assays, often with no measurable biochemical inhibition despite well-defined electron density maps (30, 31). Similarly, extensive benchmarking has shown that docking methods can correctly reproduce crystallographic poses while failing to predict affinity or rank ligands by potency (32). The accumulated literature highlights a challenge in structure-based design, molecules can adopt a chemically sensible pose yet still fail to bind with useful strength. Our ChAT ligands represent a clear example of this disconnect. Although they reproduce important recognition elements of the **1-CoA** binding mode, the free energy of binding they provide for the protein–ligand complex is insufficient to generate measurable affinity in solution.

The TSA measurements of thermal stability of the enzymes provided functional distinctions between ChAT and CrAT. AVPs stabilized ChAT only in the presence of CoA, whereas no CoA-dependent stabilization was observed for CrAT, consistent with the lack of hydrothiolation reactivity in the latter. Moreover, the stability experiments underscored the chemical vulnerability of ChAT. ChAT contains an unusually high number of cysteine residues, and UHPLC-HRMS mapping showed that CPM alkylates at least ten of them including residues in a reactive Cys266-Ile267-Cys268-Leu269-Val270-Cys271 (CXCXXC) motif. Furthermore, DTNB also alkylates Cys268 (Fig. S10) in agreement with previous studies (33). In contrast, CrAT is modified at only three cysteines. This extensive covalent modification explains the pronounced destabilization and loss of catalytic activity observed in the CPM assay. CPM has previously been employed as a probe in microscale fluorescent thermal stability assays of membrane proteins and as a probe for hyperreactive thiols on the skeletal sarcoplasmic reticulum ryanodine receptor (RyR1) due to its ability to modify cysteine residues (34, 35). These findings emphasize that CPM-based assays require particular caution (26). Furthermore, compounds that appeared active in the CPM assays produced similar dose-response curves in enzyme-free counter-screens, demonstrating that signal loss arose from assay interference rather than inhibition. CPM assays may still serve a role in high-throughput settings, but only when accompanied by careful controls such as counter-screens, shortened incubation times, and orthogonal biophysical validation. A further indication of assay interference emerged from the unusually steep Hill slopes observed for several compounds in the CPM assay. Hill coefficients far above unity are inconsistent with simple 1:1 reversible inhibition and instead suggest nonspecific mechanisms that amplify assay signal independently of true enzyme engagement. Aberrant curve shapes therefore serve as an additional diagnostic signature reinforcing that the CPM assay is unreliable for evaluating ChAT ligands in the absence of orthogonal validation. SPR was therefore essential for establishing a reliable positive control compound for reversible binding. The CoA-analog S-ethyl-CoA exhibited nanomolar binding affinity for ChAT, confirming proper folding and full activity of the immobilized protein. In contrast, none of the designed molecules resulted in measurable binding affinities, confirming the absence of relevant reversible binding in solution and underscoring that crystallography alone cannot be used as an orthogonal validation method for ChAT ligand development.

Although the designed compounds adopted the expected binding pose in ChAT, the **11**•CrAT crystal structure revealed a distinct vestibule-binding mode. Our selectivity studies indicated that ChAT and CrAT differ fundamentally in their ability to support the *in situ* hydrothiolation reaction between AVPs and CoA. ChAT exhibited robust CoA-dependent ΔT_m_ increases of 8.5 to 14 °C for AVP-CoA adducts, whereas CrAT showed only a modest, uniform ∼2.5 to 2.8 °C stabilization regardless of the AVP present, mirroring the effect of CoA alone. These observations indicate that ChAT, but not CrAT, can position AVPs and CoA in a geometry favorable to productive thia-Michael chemistry, consistent with ChAT’s ability to generate covalent AVP-CoA inhibitors *in situ*. CrAT, by contrast, either does not catalyze the hydrothiolation reaction or stabilizes the resulting adducts too weakly for detection under these conditions. The structural studies show that the designed compounds can occupy the same ChAT tunnel region as **1-CoA**, but their interaction energies are insufficient to support measurable binding or functional inhibition when not in adduct with CoA as the Michael acceptor motif enables. In other words, ChAT readily aligns AVP-like scaffolds for covalent chemistry, as evidenced by the strong CoA-dependent stabilization, yet this geometric compatibility does not translate into strong noncovalent affinity without CoA attached. These results illustrate that a correct pose does not guarantee meaningful affinity, and the capacity of ChAT to stabilize covalent CoA adducts does not imply that the same scaffold will be retained through purely noncovalent interactions.

Beyond the specific ligand series investigated here, the results of this study provide several broader insights into ligand recognition and inhibitor discovery for choline/carnitine O-acyltransferases. First, our data demonstrate that commonly used thiol-reactive reporter systems such as CPM can produce misleading results when applied to cysteine-rich enzymes such as ChAT. Mass spectrometry analysis showed that CPM rapidly forms covalent adducts with multiple cysteine residues in ChAT, leading to time-dependent loss of catalytic activity. Second, the study highlights an important limitation of relying solely on crystallographic complex formation as evidence of ligand binding. Although several compounds could be visualized in the ChAT active site tunnel in crystal structures, solution-phase measurements indicate that these complexes likely reflect weak interactions that become observable only under the high ligand concentrations used during crystal soaking. Third, our results suggest enzyme-specific reactivity within the choline/carnitine O-acyltransferase family. TSA showed that AVPs undergo the previously described *in situ* hydrothiolation reaction in ChAT but do not produce a comparable stabilization effect in the homologous enzyme CrAT under the same conditions. This difference is consistent with earlier structural observations that the hydrophobic pocket accommodating the aromatic substituent of AVPs differs between ChAT and the CrAT subgroup. This observation suggests either that the hydrothiolation reaction does not occur efficiently in CrAT or that the resulting product does not significantly stabilize the CrAT enzyme. In either case, the results indicate that productive interactions of the AVP scaffold are favored in ChAT relative to CrAT. Structural comparisons further suggest that this enzyme preference may arise from differences in residues lining the hydrophobic pocket adjacent to the catalytic site. Taken together, these findings illustrate how enzyme-specific chemical reactivity, assay interference, and inherent limitations of X-ray crystallography can complicate inhibitor discovery efforts, and they emphasize the importance of integrating orthogonal biochemical, structural, and biophysical approaches when evaluating ligand interactions with cysteine-containing enzymes.

In conclusion, this study demonstrates that correct active-site recognition, as assessed by molecular docking and X-ray crystallography, is not sufficient to confer meaningful reversible inhibition of ChAT. Although the compounds designed and synthesized here adopted the expected **1-CoA** derived binding pose, biophysical and biochemical measurements revealed that these interactions were weak in solution and do not translate into functional enzyme inhibition. Apparent inhibitory activity, including steep dose–response behavior and nominal IC_50_ values, was shown to arise from assay interference rather than enzyme inhibition, emphasizing the necessity of enzyme-free counter-screens and orthogonal validation.

A central finding is the profound susceptibility of ChAT to react with thiol-reactive molecules, such as the reagent CPM. The high cysteine content of the enzyme, including reactive motifs distal to the catalytic site, renders CPM-based assays particularly vulnerable to artefactual readouts. These results highlight the importance of careful assay development, including strict control of incubation times and reaction conditions, as well as verification of enzymatic activity. Importantly, when appropriate controls and counter-screens are applied, CPM-based assays remain functional and provide a valuable tool for ChAT inhibitor screening.

Together, these results show that reliable assessment of reversible ligand binding to ChAT requires early integration of biophysical methods such as SPR or TSA, alongside carefully controlled biochemical assays. The compounds presented herein are therefore unsuitable for further development as ChAT inhibitors, but they provide instructive examples of the limitations of structure only validation and thiol-dependent assay formats.

## Experimental procedures

### Materials

A plasmid encoding recombinant *Homo sapiens* ChAT isoform R residue 1 to 630 (*h*ChAT) was kindly provided by Dr Brian Shilton (Department of Biochemistry, Schulich School of Medicine & Dentistry, University of Western Ontario). The construct contains an N-terminal hexahistidine tag and a tobacco etch virus protease cleavage site and has been described previously (36). ChAT-SERM has been described earlier (12). The expression construct used to produce *H. sapiens* CrAT was based on the DNA sequence corresponding to amino acid residue 23 to 626 of *H. sapiens* CrAT isoform b (CRA_b) modified with the sequence MRGSHHHHHHTDPLPRLPVPPL at the N terminus (37). The gene was codon optimized, synthesized, and inserted to a pD451-SR vector by ATUM. The construct used to express *H. sapiens* AChE has been described previously (38).

### Protein purification

The plasmids of ChAT and CrAT were transformed into the BL21(DE3) strain of *Escherichia coli* and plated on Luria agar plates supplemented with appropriate antibiotics. One colony was inoculated in 100 ml Luria broth (LB) supplemented with carbenicillin at a concentration of 100 μg/ml or kanamycin at a concentration of 50 μg/ml. The starter culture was incubated 16 to 20 h at a temperature of 37 °C, whereupon 10 to 20 ml was transferred to 2 L LB or 2 L autoinduction media in 5 L baffled culture flasks. At an absorbance of 0.3 to 0.6 at a wavelength of 600 nm, the temperature was reduced to 17 °C. For cultures grown in LB, the protein production was induced after an additional 30 to 60 min incubation by addition of 500 μM Isopropyl β-D-1 thiogalactopyranoside (Sigma-Aldrich). Following a 16 to 20 h incubation, the bacteria was pelleted by centrifugation 10,000*g* for 20 min. The pellet was resuspended in 30 ml of PBS and following an additional centrifugation, the supernatant was discarded, and the pellet was stored at a temperature of −20 °C until purification. For purification, the pellet was thawed and diluted in 30 ml of buffer A (50 mM Tris–HCl pH 8.0, 1 M NaCl, 25 mM imidazole, 5% (v/v) glycerol, and 50 μM tris(2-carboxyethyl)phosphine (TCEP) supplemented with 5 mg lysozyme. Following 10 min incubation, the cells were cooled to 4 °C and lysed by sonication. The lysate was centrifuged 40,000g for 40 min whereupon the supernatant was loaded on a 5 ml HisTrap crude column (Cytiva). The column was washed with buffer A until a stable baseline was reached, and the sample was eluted using a gradient of buffer B (buffer A supplemented with 500 mM imidazole). Pooled fractions were immediately loaded on a HiPrep 26/10 desalting column (GE HealthCare) equilibrated with buffer C (20 mM Hepes pH 7.0, 50 mM NaCl, 5% (v/v) glycerol, and 50 μM TCEP) for ChAT and buffer D (25 mM Tris–HCl pH 8.0, 50 mM NaCl, 5% (v/v) glycerol and 50 μM TCEP) for CrAT. The pooled fractions were loaded on a 6 ml ResourceS (ChAT) or a 6 ml ResourceQ (CrAT) ion-exchange chromatography column (Cytiva) that were washed until a stable baseline was reached. ChAT was eluted by a gradient against buffer E (buffer C + 1 M NaCl) and CrAT was eluted by a gradient against buffer F (buffer D + 1 M NaCl). Fractions containing protein were pooled and immediately loaded on a HiPrep 26/10 desalting column (GE HealthCare) equilibrated with storage buffer (20 mM Hepes pH 7.5, 150 mM NaCl, 5% (v/v) glycerol, and 50 μM TCEP. The protein was concentrated, and the concentration was determined using Abs1% constants of 0.772 and 1.004 for ChAT and CrAT, respectively.

#### TSA assay

The dye GloMelt (Biotium, 20 × ) was prepared using dH_2_O. Purified protein was thawed on ice. Stocks of sample compounds at an appropriate concentration (*i.e.* 1 mM or 10 mM) were prepared in DMSO. The samples were prepared in 1.5 ml test tubes using the stock solutions, and the volume was adjusted to 90 μl using dH_2_O. The samples were centrifuged 20,000*g* for 2 min. and the experiment was subsequently initiated by the addition of 10 μl protein. The samples were gently mixed, briefly centrifuged and divided to four wells (*i.e.* four replicates, each well containing 20 μl of the sample) of a 96-well plate. The final concentration in the plate was GloMelt (200 × ), AVPs 100 μM, CoA 100 μM and 10% DMSO. The plate was sealed, briefly centrifuged and the shift in florescence was measured in a C1000 CFX96 Real Time Thermal Cycler (Bio-Rad) using a temperature gradient resolution of 0.1 °C and a scanning range of 25 to 90 °C. The raw data were exported, and the melting points were extracted with the semiautomated software TSA-CRAFT (39).

#### Molecular docking simulations

The 3D structure with pdb code 7AMD was imported to Maestro (40). Crystal waters and **1-CoA** were removed. The complex was subjected to the protein preparation process in Schrödinger (41), where the maximal root-mean-square deviation (RMSD) was set to 0.3 Å, and the OPLS4 force field was used. Two modifications were made during the protein preparation process within 6 Å from the ligand, His324 was modified to another tautomer, and Glu437 was protonated. Glide SP (standard precision) was used for molecular docking (42). The grid box was based on the truncated **1-CoA** to the length corresponding to **7** (**1-CoA** short) leading to a center of the box close to the sulfur atom of **1-CoA** short. During the docking, 5000 poses per ligand were kept for the first step, and 400 poses per ligand were subjected for post docking energy minimization. A maximum of 100 poses per ligand were saved, with a maximum atomic displacement of less than 0.3 Å. The designed compounds were imported to Maestro as smiles and converted into 3D structures and were processed through ligand preparation within Schrödinger package before molecular docking.

Glide SP score values were used for evaluation, where the best score value for each ligand were used as a first filter. Poses with a docking score of ≥ 90% of the best individual score values were further analyzed. All surviving poses were visually inspected and classified based on overlap with **1-CoA**. Poses where the different parts of the ligands overlapped with the corresponding parts of the crystal ligand based on the design were classified as similar X-ray poses (*i.e.* similar to the X-ray pose of **1-CoA**), and alternative poses, where some deviations were observed. All other poses were classified as discarded.

#### Synthesis

Experimental procedures for the chemical synthesis, purification, and characterization presented herein in including ^1^H- and ^13^C-NMR data can be found in Supp. Data S1 and Data S2.

#### Biochemical assays

The activity of ChAT and CrAT was determined by measuring the fluorescence of the reaction product between the thiol of CoA and the probe CPM (22). The assays were performed in 20 mM Hepes pH 7.5, 500 mM NaCl, 5% (v/v) glycerol. Stock solutions of CPM (600 μM) was prepared in DMSO, bovine serum albumin (BSA) (4 mg/ml) was prepared in dH_2_O whereas stocks of choline chloride (ChCl, 20 mM) and AcCoA (800 μM) was prepared in the above-mentioned buffer. The compounds were dissolved to a concentration of 100 mM in DMSO and dilution series thereof were also prepared in DMSO, final compound concentrations in the assay generally ranged from 0.1 μM to 1 or 2 mM with a final DMSO concentration of 10%. Following dissolution of stock solutions followed a rapid and standardized protocol: the reagents were immediately aliquoted, frozen, and stored at −80 °C until use, except for the ChCl which was prepared fresh prior to use. A master mix of buffer, protein and BSA was prepared in advanced and stored at −80 °C until use. The required assay reagent aliquots were thawed on ice whereupon 80 μl master mix were added per well in a black 96 well plate with flat, transparent bottom followed by addition of 5 μl of the remaining reagents in a specific order (CPM, compound, ChCl, and AcCoA). The sample column contained all reagents and a dilution series of the test compound whereas in the corresponding negative control column ChCl was substituted for dH_2_O. The final concentrations in the plate were 3 μM BSA, 30 μM CPM, 1 mM ChCl, 40 μM AcCoA, 10% DMSO, and either 35.5 nM ChAT or 7.2 nM CrAT. To mitigate the risk of false positives, we also employed a counter-screen assay, incubating compounds with CPM and CoA in the absence of enzyme and choline to identify reagent interference. The fluorescence signal of the reaction product was followed for 1 minute in a FlexStation 3 Multi-Mode Microplate Reader (Molecular Devices, LLC.CA) using an excitation wavelength of 384 nm and emission wavelength of 469 nm. Immediately following the kinetic analysis, the plate was read using absorption at 700 nm to detect insoluble compounds. In the following analysis, values corresponding to two times the background absorption of the sample, that is Abs700 > 0.1, were marked as compounds with solubility issues.

To measure the activity of AChE, we employed the Ellman assay adapted to a 96-well plate format, where the hydrolysis of acetylthiocholine is monitored over time (29). This AChE-catalyzed reaction produces thiocholine, which in turn reacts with the thiol-containing reagent DTNB to generate a product that can be quantified spectrophotometrically. The assay was performed in 0.1 M sodium phosphate buffer (pH 7.4) at 30 °C with the final concentration of the substrate acetylthiocholine iodide at 1 mM, and the reagent DTNB at 0.2 mM. Dose-response curves were obtained by nonlinear regression curve fit of the relative fluorescence units per second (for ChAT and CrAT) or absorbance units per second (for AChE) *versus* logarithmized inhibitor concentration. IC_50_-values were determined using the log(inhibitor) versus response variable slope (four parameters) equation in GraphPad Prism version nine for Windows (GraphPad Software).

#### Surface plasmon resonance

SPR measurements were performed on a Biacore 1K instrument (Cytiva). All experiments were run at 25 °C. ChAT was diluted to 20 μg/ml in 0.01 M phosphate buffer pH 7.0 (immobilization buffer) and immobilized on a CM5 sensor chip (Series S Sensor chip CM5, Cytiva) using amine coupling. The chip surface of one flow cell was first activated with 1-Ethyl-3-[3-dimethylaminopropyl]carbodiimide (EDC) and N-hydroxysuccinimide (NHS) at an EDC/NHS 1:1 M ratio, ChAT was coupled to the surface (10 μl/min, 420 s contact time) and unbound carboxyl groups were then sealed with ethanolamine. An untreated flow cell was used as reference for background subtraction. After a stabilization period of 5 h, compounds were injected using multi cycle kinetics. For CoA and S-ethyl-CoA interaction studies, PBS supplemented with 0.05% Tween-20, 50 μM TCEP, and 0.1% BSA, pH 7.4, was used as running buffer. CoA was diluted twofold from 200 to 0.1 μM, and S-ethyl-CoA to 25 μM and then 3-fold from 12.5 μM to 0.2 nM, both dilution series in running buffer. For affinity measurements of compounds **1**, **(E)-9**, **22**, **23,** and **24** (7.8–600 μM) and **11** (8.8–600 μM), 5% DMSO was included in the running buffer to match sample and buffer refractive index. Solvent correction was performed to account for the DMSO content. For affinity measurements, compounds were injected over the surfaces at 30 μl/min, 120 s contact time, followed by 600 s dissociation. Measurements were performed in technical duplicates. Resulting sensorgrams were reference and blank subtracted prior to fitting to a steady-state affinity binding model using the Biacore Insight Evaluation Software (Cytiva).

### Mass spectrometric analysis of CPM and DTNB adducts

The protein (20 μg in 20 mM Hepes pH 7.5, 150 mM NaCl, 5% (v/v) glycerol, and 50 μM TCEP) was incubated for 2 min with CPM (240 μM for ChAT and 48 μM for CrAT) or DTNB (30 mM, ChAT only) and precipitated using addition of 1000 μl ice-cold methanol. After 30 min in freezer, precipitates were collected using 5 min centrifugation at 14,000 g. This was repeated three times in order to minimize the chromophore content. After drying, 8 μl 0.5 M ammonium bicarbonate, 5 μl trypsin (0.2 μg/μl), and 27 μl MilliQ was added. The digestion reaction was carried out at 45 °C, with gentle shaking. The reaction was terminated after 60 min by addition of 10 μl 10% formic acid. Thereafter 30 μl sample was transferred to an insert glass vial and diluted with 20 μl milliQ water. Analysis were carried out using a nano-LC system (Thermo UltiMate 3000 RSLC) connected to a Bruker QTOF mass spectrometer (MaXis Impact HD) equipped with a nano electrospray ion source (CaptiveSpray nanoBooster) boosted with acetonitrile. Mass range m/z 300 to 2200, scan frequency 2 Hz, collision energy 100 eV, ionization mode positive. The LC system was operated in microliter injection mode, and 1 μl sample was trapped on a 20 mm, 100 μm i.d. C18 trap column (5 μm Acclaim PepMap, Thermo Fisher Scientific) using 98% eluent A (water with 0.1% formic acid) and 2% B (acetonitrile with 0.1% formic acid), at a flow rate of 10 μl/min. After 5 min, the trapping valve was switched, and the peptides were separated on a 150 mm, 75 μm i.d. C18 analytical column (2 μm Acclaim PepMap, Thermo Fisher Scientific) using a 20 min linear A/B-gradient from three to 40% B. The gradient was then ramped to 60% B for 3 min, then to 99% B and kept there for 5 min before returning to 3% B. The total sample to sample time was 45 min. After each injection, the sample loop was flushed with 95% B at 100 μl/min for 6 min in order to minimize carryover in the system. Data analysis was performed with Bruker Daltonics software Compass DataAnalysis 4.2. Search parameters; enzyme trypsin, peptide tolerance + - 15 ppm, fragment mass tolerance + - 0.05 Da, max missed cleavages 4, database in-house ChAT. Modifications of CPM (+402.1579) and DTNB (+197.9867) was added to the search engine and were the only two modifications included for all samples.

### Protein crystallization and ligand soaking

ChAT-SERM was purified and crystallized as previously described (12), with the modification that all buffer exchanges were performed in Amicon Ultra centrifugal filter units (30 kDa molecular weight cutoff). Protein used for crystallization trials was concentrated to 10 to 15 mg/ml in 20 mM Tris–HCl pH 8.5, 100 mM NaCl, 0.5 mM TCEP, and 10% (v/v) glycerol, and immediately flash frozen in aliquots in liquid nitrogen. To generate enzyme-inhibitor complexes, ChAT-SERM crystal soaking was performed in a two-step procedure. First, 1 to 2 μl of mother liquid was added to the crystal drop and after 2 min of incubation, 3 μl of a 10 mM solution of **12** or **17**, or 20 mM a solution of **(E)-7**, in 10% (w/v) PEG3350, 100 mM Tris–HCl pH 8.5, and 20% (v/v) DMSO was added stepwise over 3 to 4 min. Following incubation for 3 to 4 h, the crystals were mounted in a loop and vitrified in liquid nitrogen. CrAT at 13 mg/ml in 20 mM Hepes pH 7.5, 150 mM NaCl, 5% (v/v) glycerol, and 50 μM TCEP was crystallized using the hanging drop vapor diffusion approach using a mother liquid consisting of 20% (w/v) PEG2000, 50 mM Bis-Tris pH 6.0, and 20% (v/v) glycerol. Crystals typically grew over 3 to 4 days at a temperature of 20 °C. To generate enzyme-inhibitor complexes of CrAT, 0.5 μl of a 100 mM solution of **22** in DMSO, was added to the crystals. Following incubation for 2 to 3 h, the crystals were mounted in a loop and vitrified in liquid nitrogen.

#### Data collection and structure determination

Data were collected at 100°K on the MAX IV Biomax beamline using an EIGER 16M Hybrid-pixel Detector (Dectris). The oscillation range was 0.1 ° for each exposure and the datasets consisted of 3600 frames collected at a wavelength of 0.976 Å. Intensity data were indexed and integrated with XDS and autoPROC (43, 44), and scaled using Aimless in the CCP4 suite (45). Initial models of **6**•ChAT-SERM, **8**•ChAT-SERM, **(E)-9**•ChAT-SERM and **11**•CrAT were determined using difference Fourier methods using the phenix program suite (46), starting from the coordinates of the ChAT apo structure 2FY2.pdb and the CrAT apo structure 1NM8.pdb, respectively. For subsequent refinement and manual rebuilding, the programs phenix.refine and COOT were used (47, 48). The atomic coordinates were modeled according to the 2|F_o_-F_c_| and |F_o_-F_c_| maps. Angle and bond restraints of the ligands were optimized in phenix.elbow using the eLBOW AM1 algorithm. Statistics of data collection and refinement are given in Table S4.

## Data availability

Data supporting the findings of this study are available within the article and its supporting information. The refined crystal structure coordinates and structure factors have been deposited in the Protein Data Bank with accession codes 9RT3, 9F85, 9F84, and 9SCK. Requests for additional data information may be directed to the corresponding author.

## Supporting information

This article contains supporting information with citations: (12).

## Declaration of Generative AI and AI-Assisted Technologies in the Writing Process

During the preparation of this work the authors used OpenAI’s ChatGPT for language refinement. After using this tool/service, the authors reviewed and edited the content as needed and take(s) full responsibility for the content of the published article.

## Conflict of interest

Daniel Wiktelius is an employee of AstraZeneca. The other authors declare that they have no conflicts of interest with the contents of this article.
