# Supplementary material for: Analysis of ligand recognition by choline O-acetyltransferase reveals thiol-reactive assay interference and weak ligand affinity in solution
Source: J Biol Chem. 2026 May 7;302(6):113115. doi: 10.1016/j.jbc.2026.113115 (PMC13255071; doi:10.1016/j.jbc.2026.113115)
Supplement: Supporting Information [file mmc1.docx]

**Supporting Information**

Analysis of ligand recognition by choline O-acetyltransferase reveals thiol-reactive assay interference and weak ligand affinity in solution

Nina Forsgren^a^,$, Frida Jonsson^a^,$, Marcus Carlsson^a^, Robin Afshin Sander^a,b^, Andreas Larsson^a^, Pernilla Lindén^a^, Anna Linusson^a,c^, Cecilia Springer Engdahl^a^, Daniel Wiktelius^a,d^, Fredrik Ekström^a,^*

^a^ CBRN Defence and Security, Swedish Defence Research Agency, SE-90621 Umeå, Sweden

^b^ Present address, Research School of Chemistry, Australian National University, Canberra 2601, Australia

^c^ Department of Chemistry, Umeå University, Umeå SE-90187, Sweden

^d^ Disclosure: Daniel Wiktelius is an employee of AstraZeneca

*Corresponding author

Fredrik Ekström: E-mail address: fredrik.ekstrom@foi.se

$N.F and F.J. contributed equally

This file includes

Supporting Data S1

Supporting Data S2

Supporting Table S1

Supporting Table S2

Supporting Table S3

Supporting Table S4

Supporting Figure S1

Supporting Figure S2

Supporting Figure S3

Supporting Figure S4

Supporting Figure S5

Supporting Figure S6

Supporting Figure S7

Supporting Figure S8

Supporting Figure S9

Supporting Figure S10

### Supporting data S1- Synthesis

**General Experimental Procedures**. H^1^ and C^13^ NMR spectra were recorded on a Bruker DRX-500 spectrometer (Bruker, Billerica, MA, USA) at 298 K. ^1^H and ^13^C chemical shifts are reported relative to CHCl_3_ (δ_H_ 7.26 ppm) or CDCl_3_ (δ_C_ 77.16 ppm); DMSO-*d*_6_ (δ_H_ 2.50 ppm) or (δ_C_ 39.52 ppm); MeOH (δ_H_ 3.33 ppm) or MeOD (δ_C_ 49.0 ppm) as internal reference. Liquid Chromatography High resolution mass spectra (LC-HRMS) data was recorded with a Dionex Ultimate 3000 RS UPLC coupled to a Bruker Daltonik Impact HD Mass Spectrometer using a Waters Acquity HSS T3, 2.1x100 mm, 1.8 μm column. Eluent A was 0.1% formic acid in Milli-Q water and B 0.1% formic acid in acetonitrile. Samples were separated using a linear 5 min gradient 2- 98% B. The mass spectrometer was run in positive mode (ESI +), monitoring m/z 50-1200 at 5 Hz. Calibration of the mass scale for each sample was done using sodium formate clusters at the beginning of each analysis. TLC was performed on silica gel 60 F_254_ (Merck Millipore) with detection of UV light. Flash Column chromatography [eluent for flash chromatography is given between brackets in the experimental section] was carried out on silica gel (particle size, 60Å, 230-400 mesh) Sigma-Aldrich. Preparative HPLC was performed using VP 150/21 Nucleodur C-18, HTEC, 5µm column (Macherey-Nagel) on a Gilson PLC2050 Prep-Scale system with a flow rate of 20 mL/min connected with an diode array detector, and an CH_3_CN (0.% HCO_2_H)/H_2_O (0.1% HCO_2_H) eluent system. Building blocks and reagents were purchased from Merck KGaA/ Sigma-Aldrich and solvents were purchased from Fisher Scientific. The synthesis of compounds **1-5,** and **1-CoA** have been described previously (12). The compounds were purified to > 95% purity. As we have reported before (12), compounds of type B in Fig. 4 suffers from retro-*thia*-Michael reaction, *i.e.* spontaneous reversal to their starting materials (AVP and thiol). The rate and extent of the retro-reaction is modified by which thiol and AVP that is used and under which conditions the compound is stored and handled.

*S-Ethyl Coenzyme A* (**S-ethyl-CoA**)*.* Ethyl iodide (52 µl, 637 µmol) was added to Coenzyme A trilithium salt (100 mg, 127 µmol) and lithium carbonate (28 mg, 382 µmol) in water (2 ml). The vial was capped and the reaction stirred for ca 3h at RT before being purified by preparative RP-HPLC [H2O:MeCN, NH4HCOO 0.1% w/w] and lyophilized to afford the product (**S-ethyl-CoA**) as a white solid in quantitative yield (103 mg).^1^H NMR (500 MHz, D_2_O) δ 8.53 (s, 1H), 8.45 (s, 3H), 8.23 (s, 1H), 6.16 (d, *J* = 6.8 Hz, 1H), 4.86 – 4.80 (m, 1H), 4.57 (br. s, 1H), 4.25 – 4.20 (m, 2H), 4.00 (s, 1H), 3.85 – 3.78 (m, 1H), 3.58 – 3.51 (m, 1H), 3.45 (td, *J* = 6.6, 3.3 Hz, 2H), 3.32 (t, *J* = 6.8 Hz, 2H), 2.63 (t, *J* = 6.7 Hz, 2H), 2.53 (q, *J* = 7.6 Hz, 2H), 2.45 (t, *J* = 6.5 Hz, 2H), 1.18 (t, *J* = 7.4 Hz, 3H), 0.87 (s, 3H), 0.74 (s, 3H).^13^C NMR (126 MHz, D_2_O) δ 174.67, 173.81, 171.00, 155.54, 152.82, 149.28, 139.79, 118.54, 86.43, 83.71 (dd, *J* = 9.1, 4.4 Hz), 74.08, 73.99 (d, *J* = 4.0 Hz), 73.80 (d, *J* = 4.9 Hz), 71.81 (d, *J* = 6.2 Hz), 65.51 (d, *J* = 5.4 Hz), 38.58, 38.25 (d, *J* = 8.2 Hz), 35.39, 35.30, 29.75, 24.92, 20.77, 18.07, 13.86.

**
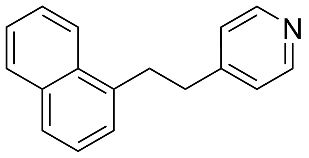
**

*4-[2-(naphthalen-1-yl)ethyl]pyridine*. The synthesis of 4-[2-(naphthalen-1-yl)ethyl]pyridine has been previously published (1).

**
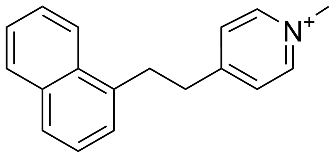
**

*1-methyl-4-[2-(naphthalen-1-yl)ethyl]pyridin-1-ium iodide* (**6**)**.** Methyl iodide (27 µl, 10 eq.) was added to a solution of 4-[2-(naphthalen-1-yl)ethyl]pyridine (1 eq., 10 mg) in CH_3_CN (300 µl) and CHCl_3_ (150 µl). The solution was stirred o.n. in the dark at r.t. and purified by trituration with ether which gave **6** (15.3 mg, 41 µmol) as white crystals in 95% yield. ^1^H NMR (500 MH*z*, CDCl_3_): δ 8.83 (d, J = 6.7 Hz, 2H), 8.02 (d, J = 8.3 Hz, 1H), 7.89 (d, J = 7.9 Hz, 1H), 7.80 – 7.72 (m, 4H), 7.62 – 7.47 (m, 1H), 7.36 (t, J = 7.6 Hz, 1H), 7.20 (d, J = 6.7 Hz, 1H), 4.45 (s, 3H), 3.51 (t, J = 7.7 Hz, 2H), 3.36 (t, J = 7.6 Hz, 2H); ^13^C NMR (125 MH*z*, CDCl_3_): δ 162.32, 144.77, 134.77, 134.15, 131.46, 129.28, 128.39, 127.92, 126.79, 126.68, 126.08, 125.64, 123.14, 48.43, 36.66, 32.75; HRMS [M]^+^ calculated for [C_18_H_18_N] ^+^: 248.1435; found, 248.1432.

*4-(naphthalen-1-ylethynyl)pyridine.* 4-bromopyridine hydrochloride (0.4 g, 2.19 mmol), Pd(PPh_3_)_4_ (60 mg, 0.052 mmol), and CuI (19 mg, 0.100 mmol) were mixed in DME (6 ml) and TEA (1.5 ml).  1-ethynyl naphthalene (943 mg, 4.11 mmol) was added to the mixture and the reaction was stirred at 80°C overnight under argon atmosphere. The resulting mixture was diluted with Et_2_O, filtrated through celite, and concentrated. Flash chromatography over silica [heptane:EtOAc 4:1, 2:1] gave the product [429 mg, 1.87 mmol] in 91% yield. ^1^H NMR (500 MH*z*, CDCl_3_): δ 8.66 (d, *J*=6.0 H*z*, 2H), 8.38 (d, *J*=8.4 H*z*, 1H) 7.92-7.88 (m, 2H), 7.80 (dd, *J_1_*=7.2 H*z*, *J_2_*=1.8 H*z* 1H), 7.65-7.54 (m, 2H), 7.51-7.48 (m, 3H); ^13^C NMR (125 MH*z*, CDCl_3_): δ 150.0, 133.3, 131.7, 131.3, 130.0, 128.6, 127.3, 126.8, 126.0, 125.7, 125.4, 119.8, 92.3, 91.6.

*(Z)-2-((1-(naphthalen-1-yl)-2-(pyridin-4-yl)vinyl)thio)ethan-1-amine*. 4-(naphthalen-1-ylethynyl)pyridine (524 mg, 2.287 mmol) and Cs_2_CO_3_ (745, 2.287 mmol) were mixed with cysteamine (863 mg, 11.186 mmol) and stirred for 16 h at room temperature. The resulting mixture was purified using Flash chromatography [DCM: TEA 200:1; DCM:MeOH:TEA 200:1:1] which gave the amine product (189 mg, 0.617 mmol) in 27% yield: ^1^H NMR (500 MH*z*, CDCl_3_): δ 8.64 (d, *J*=6.2 H*z*, 2H), 8.27-8.22 (m, 1H), 7.93-7.85 (m, 2H), 7.61 (d, *J*=6.2 H*z*, 2H), 7.56-7.45 (m, 4H), 6.57 (s, 1H), 2.58 (t, *J*=6.5 H*z*, 2H), 2.31 (t, *J*=6.5 H*z*, 2H), 1.11 (broad s, 2H); ^13^C NMR (125 MH*z*, CDCl_3_): δ 150.0, 143.9, 142.2, 137.4, 133.7, 131.5, 129.0, 128.6, 127.5, 126.9, 126.9, 126.5, 125.4, 125.3, 123.7, 41.9, 36.8.

(*Z)-N-(2-((1-(naphthalen-1-yl)-2-(pyridin-4-yl)vinyl)thio)ethyl)acetamide* (**7**). To a solution of (Z)-2-((1-(naphthalen-1-yl)-2-(pyridin-4-yl)vinyl)thio)ethan-1-amine (110 mg, 0.356 mmol) and TEA (75.1 µl, 0.538 mmol) in DCM (10 ml), AcCl (38.5 µl , 0.538 mmol) was added and the reaction was stirred at room temperature. for 1 h. The resulting mixture was concentrated and purified by Flash chromatography [DCM:MeOH, 100:1-10:1] which gave **7** (83 mg, 0.238 mmol) in 67 % yield. ^1^H NMR (500 MH*z*, CDCl_3_): δ 8.66 (d, *J*=6.1 H*z*, 2H), 8.26-8.21 (m, 1H), 7.93-7.87 (m, 2H), 7.59 (d, *J*=6.1 H*z*, 2H), 7.57-7.46 (m, 4H), 6.62 (s, 1H), 5.42 (broad s, 1H), 3.16 (q, *J*=6.2 H*z*, 2H), 2.39 (t, *J*=6.2 H*z*, 2H), 1.83 (s, 3H); ^13^C NMR (125 MH*z*, CDCl_3_): δ 169.9, 150.0, 143.8, 141.3, 137.0, 133.8, 131.4, 129.3, 128.8, 128.4, 127.1, 127.0, 126.6, 125.4, 125.2, 123.8, 39.1, 32.6, 23.3; HRMS [M+H]^+^ calculated for [C_21_H_21_N_2_OS] ^+^: 349.1369; found, 349.1367.

**
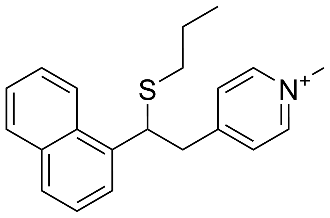
**

*1-methyl-4-[2-(naphthalen-1-yl)-2-(propylsulfanyl)ethyl]pyridin-1-ium* *formate* (**8**)**.** To (E)-4-(2-(naphthalen-1-yl)vinyl)pyridine (50 mg, 1 eq) was added n-propanthiol (314 µl, 16 eq) and TEA (60 µl, 2 eq) and the reaction was capped and stirred at 85C for two weeks before being purified by flash chromatography. Methyl iodide (6.6 µl, 6 eq.) was added to a solution of the intermediate pyridine (5.5 mg, 1 eq) in CH_3_CN (80 µl) and CHCl_3_ (160 µl). The solution was stirred in the dark at r.t. o.n. before being concentrated under reduced pressure to give **8** as a glass in 24% yield.: ^1^H NMR (500 MHz, MeOD) δ 8.69 (dd, *J* = 5.1, 1.7 Hz, 2H), 7.98 (dd, *J* = 5.1, 1.7 Hz, 2H), 7.88 (d, *J* = 8.2 Hz, 1H), 7.75 (d, *J* = 8.2 Hz, 1H), 7.59 (ddd, *J* = 8.5, 6.8, 1.4 Hz, 1H), 7.51 (ddd, *J* = 8.0, 6.8, 1.2 Hz, 1H), 7.31 (dd, *J* = 8.2, 7.0 Hz, 1H), 7.23 (dd, *J* = 6.9, 1.1 Hz, 1H), 4.54 (dd, *J* = 8.8, 6.6 Hz, 1H), 4.32 (s, 3H), 3.82 (dd, *J* = 14.3, 6.7 Hz, 1H), 3.63 (dd, *J* = 14.3, 8.7 Hz, 1H), 2.43 – 2.27 (m, 2H), 1.52 – 1.41 (m, 2H), 0.86 (t, *J* = 7.4 Hz, 3H); 13C NMR (126 MHz, MeOD) δ 164.72, 146.26, 135.39, 134.22, 132.89, 130.06, 129.02, 128.98, 128.22, 127.60, 126.94, 126.32, 124.24, 79.38, 50.25, 39.40, 34.55, 23.39, 13.52; HRMS [M]^+^ calculated for [C_21_H_24_NS] ^+^: 322.1624; found, 322.1623.

*(Z)-4-(2-((2-acetamidoethyl)thio)-2-(naphthalen-1-yl)vinyl)-1-methylpyridin-1-ium iodide* (**9**). To a solution of **7** (90 mg, 0.258 mmol) in acetonitrile (1 ml) and DCM (0.5 ml), CH_3_I (19 µl, 0.305 mmol) was added and the reaction was stirred at 50°C overnight. The resulting mixture was concentrated and triturated with Et_2_O twice and dried under vacuum which gave **9** (89 mg, 0.181 mmol) in 70% yield. ^1^H NMR (500 MH*z*, CDCl_3_): δ 8.96 (d, *J*=7 H*z*, 2H), 8.34 (d, *J*=7 H*z*, 2H), 8.08-8.03 (m, 1H), 7.93-7.87 (m, 2H), 7.56-7-49 (m, 3H), 7.44-7-40 (m, 1H), 6.94 (apparent t, *J*=6 H*z*, 1H), 6.61 (s, 1H), 4.54 (s, 3H), 3.19 (q, *J*=6 H*z*, 2H), 2.51 (t, *J*=6 H*z*, 2H), 1.98 (s, 3H); ^13^C NMR (125 MH*z*, CDCl_3_): δ 171.0, 157.5, 152.0, 144.5, 135.9, 133.6, 130.5, 130.2, 128.9, 127.6, 126.9, 126.8, 126.6, 125.4, 124.6, 122.9, 48.5, 38.6, 34.1, 23.6; HRMS [M+H]^+^ calculated for [C_22_H_23_N_2_OS] ^+^: 363.1526; found, 363.1519.

*(Z)-4-(2-((2-acetamidoethyl)thio)-2-(naphthalen-1-yl)vinyl)-1-benzylpyridin-1-ium bromide* (**10**). **7** (37 mg, 0.106 mmol) was dissolved in acetonitrile (0.2ml) and BnBr (16.4 µl, 0.138 mmol) was added and the reaction was stirred for 5 h at 80°C. The resulting solution was concentrated and triturated three times with Et_2_O. The resulting solid was dried under vacuum, which gave **10** (40 mg, 0.077 mmol) as a yellow powder in 73% yield. ^1^H NMR (500 MH*z*, CDCl_3_): δ 9.24 (d, *J*=7 H*z*, 2H), 8.26 (d, *J*=7 H*z*, 2H), 8.02-7.99 (m, 1H), 7.92-7.86 (m, 2H), 7.66-7.61 (m, 2H), 7.54-7.47 (m, 3H), 7.42-7.36 (m, 4H), 7.23 (apparent t, *J*=7 H*z*, 1H), 6.54 (s, 1H), 6.1 (s, 2H), 3.17 (q, *J*=6 H*z*, 2H), 2.51 (t, *J*=6 H*z*, 2H), 1.96 (s, 3H); ^13^C NMR (125 MH*z*, CDCl_3_): δ 171.0, 157.4, 152.1, 143.8, 135.9, 133.6, 133.2, 130.5, 130.2, 130.1, 129.8, 129.5, 128.9, 127.6, 126.9, 126.7, 126.6, 125.4, 124.5, 122.9, 63.3, 38.8, 34.0, 23.4; HRMS [M+H]^+^ calculated for [C_28_H_27_N_2_OS] ^+^: 439.1839; found, 439.1837.

*6-benzyl-2-(naphthalen-1-yl)thieno[2,3-c]pyridin-6-ium bromide* (**11**). **21** (12.4 mg, 0.047 mmol) was mixed in acetonitrile (0.3 ml) and BnBr (7.3 µl, 0.062 mmol) was added. The reaction was stirred at 60^o^C overnight and then allowed to precipitate at room temperature. After removing the solvent, the crude was triturated twice with Et_2_O. The resulting solid was dried under vacuum for 2 days which gave **11** (16.7 mg, 0.039 mmol) as a yellow solid in 82% yield; ^1^H NMR (500 MHz, CDCl_3_): δ 10.78 (s, 1H), 8.89 (dd, J_1_=1.22, J_2_= 6.79 Hz, 1H), 8.18 (d, J=6.79 Hz, 1H), 8.09-8.06 (m, 1H), 8.01 (apparent d, J=8.2 Hz, 1H), 7.97-7.94 (m, 1H), 7.73 (s, 1H), 7.72-7.68 (m, 2H), 7.64 (dd, J_1_=1.03, J_2_= 7.21 Hz, 1H), 7.61-7.54 (m, 3H), 7.43-7.35 (m, 3H), 6.35 (s, 2H); ^13^C NMR (125 MHz, CDCl_3_): δ 161.9, 149.4, 141.4, 137.9, 136.7, 134.0, 133.5, 131.6, 130.7, 130.0, 129.8, 129.7, 129.6, 129.3, 129.1, 128.1, 127.1, 125.4, 122.9, 120.5, 64.1; HRMS [M]^+^ calculated for [C_24_H_18_NS]^+^: 352.1154; found, 352.1153.

**
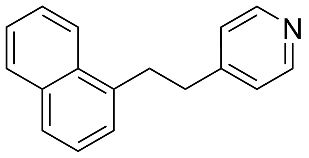
**

*4-[2-(naphthalen-1-yl)ethyl]pyridine* (**12**).The synthesis of **12** has been previously published (12).

**
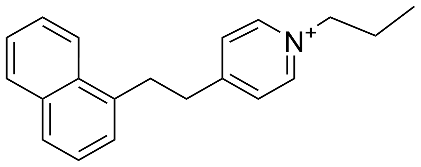
**

*4-[2-(naphthalen-1-yl)ethyl]-1-propylpyridin-1-ium iodide* (**13)** Propyl bromide (39 µl, 10 eq.) was added to a solution of **12** (1eq., 10 mg) in CH_3_CN (300 µl) and CHCl_3_ (150 µl). The solution was stirred at 70 º C o.n. and purified with trituration with ether which gave **13** (5,5 mg, 15 µmol) as an oil in 34% yield. ^1^H NMR (500 MH*z*, CDCl_3_): δ 9.28 (d, J = 6.0 Hz, 2H), 7.93 (d, J = 8.3 Hz, 1H), 7.87 (dd, J = 8.0, 1.7 Hz, 1H), 7.77 – 7.69 (m, 3H), 7.52 (dddd, J = 19.4, 8.0, 6.8, 1.4 Hz, 2H), 7.34 (dd, J = 8.3, 7.0 Hz, 1H), 7.16 (d, J = 7.0 Hz, 1H), 4.87 (t, J = 7.3 Hz, 2H), 3.47 (dd, J = 7.8, 6.3 Hz, 2H), 3.32 (dd, J = 8.5, 6.7 Hz, 2H), 2.04 (qd, J = 7.3, 7.3 Hz, 2H), 0.97 (t, J = 7.3 Hz, 3H); ^13^C NMR (125 MH*z*, CDCl_3_): δ 161.80, 144.29, 134.52, 133.95, 131.28, 129.21, 128.13, 127.90, 126.65, 126.60, 125.96, 125.49, 122.97, 62.61, 36.71, 32.78, 25.29, 10.60; HRMS [M]^+^ calculated for [C_20_H_22_N] ^+^: 276.1747; found, 276.1743.

**
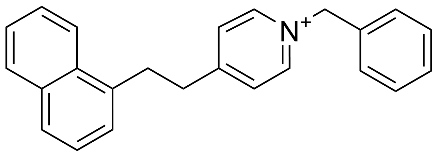
**

*1-benzyl-4-[2-(naphthalen-1-yl)ethyl]pyridin-1-ium iodide* (**14**)**.** Benzyl bromide (15 µl,3 eq.) was added to a solution of **12** (1eq., 10 mg) in CH_3_CN (300 µl) and CHCl_3_ (150 µl). The solution was stirred at 40°C and purified by trituration with ether which gave **14** (15.8 mg, 42 µmol) as an oil in 98% yield. ^1^H NMR (500 MH*z*, CDCl_3_): δ 9.45 (d, J = 6.8 Hz, 2H), 7.90 – 7.80 (m, 2H), 7.72 (d, J = 8.2 Hz, 1H), 7.69 – 7.61 (m, 4H), 7.52 – 7.41 (m, 2H), 7.37 – 7.33 (m, 3H), 7.31 (dd, J = 8.2, 7.0 Hz, 1H), 7.15 (d, J = 7.0 Hz, 1H), 6.24 (s, 2H), 3.39 (dd, J = 8.8, 6.6 Hz, 2H), 3.23 (dd, J = 8.9, 6.6 Hz, 2H); ^13^C NMR (125 MH*z*, CDCl_3_): δ 161.98, 144.39, 134.69, 133.97, 133.34, 131.31, 129.94, 129.64, 129.22, 128.05, 127.89, 126.57, 125.99, 125.57, 122.95, 63.31, 36.73, 32.65; HRMS [M]^+^ calculated for [C_24_H_22_N] ^+^: 324.1747; found, 324.1746.

**
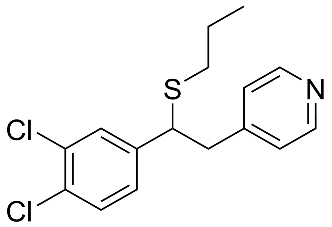
**

*4-[2-(3,4-dichlorophenyl)-2-(propylsulfanyl)ethyl]pyridine* (**15**)**.** To (E)-4-(2,3-dichlorostyryl)pyridine (250 mg, 1 eq.) was added n-propanthiol (450 µl, 5 eq) and TEA (280 µl, 2 eq). The reaction was capped and stirred at 85C for two weeks before being purified by flash chromatography over silica [heptane:EtOAC gradient] which gave **15** as a glass in 9% yield. ^1^H NMR (500 MH*z*, CDCl_3_): δ 8.45 (d, J = 6.0 Hz, 2H), 7.37 (d, J = 2.1 Hz, 1H), 7.34 (d, J = 8.3 Hz, 1H), 7.06 (dd, J = 8.2, 2.2 Hz, 1H), 6.96 (d, J = 6.1 Hz, 2H), 3.93 (dd, J = 8.6, 6.5 Hz, 1H), 3.13 (dd, J = 13.8, 6.6 Hz, 1H), 3.00 (dd, J = 13.8, 8.5 Hz, 1H), 2.34 – 2.19 (m, 2H), 1.56 – 1.41 (m, 2H), 0.89 (t, J = 7.4 Hz, 3H).; ^13^C NMR (125 MH*z*, CDCl_3_): δ 149.91, 147.12, 142.11, 132.75, 131.40, 130.56, 129.79, 127.37, 124.46, 49.62, 42.47, 33.60, 22.54, 13.54.; HRMS [M+H]^+^ calculated for [C_16_H_18_Cl_2_NS] ^+^: 326.0532; found, 326.0531.

**
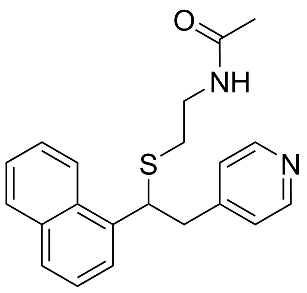
**

*N-(2-{[1-(naphthalen-1-yl)-2-(pyridin-4-yl)ethyl]sulfanyl}ethyl)acetamide* (**16**). To (E)-4-(2-(naphthalen-1-yl)vinyl)pyridine (47 mg, 1eq) dissolved in DMF (0.5 ml) was added N-acetylcysteamine (242 mg, 10 eq.) and the mixture was stirred at 100C for two days. The reaction was purified by HPLC which gave **16** as a glass in 67% yield.  ^1^H NMR (500 MH*z*, CDCl_3_): δ 8.38 (d, J = 6.1 Hz, 2H), 8.02 (br s, 1H) 7.86 (d, J = 7.9 Hz, 1H), 7.76 (d, J = 8.2 Hz, 1H), 7.54 – 7.37 (m, 3H), 7.02 (br s, 2H), 5.78 (s, 1H), 5.03 (br s, 1H), 3.34 (br. s, 2H), 3.24 (app. dq, J = 13.0, 6.5 Hz, 1H), 3.15 (app. dq, J = 14.0, 6.1 Hz, 1H), 2.48 (br. s, 0H), 1.78 (s, 3H).; ^13^C NMR (125 MH*z*, CDCl_3_): δ δ 170.07, 149.46, 147.76, 136.45, 133.94, 131.00, 129.25, 128.33, 125.84, 125.34, 124.49, 122.02, 43.99, 42.07, 38.26, 31.23, 22.99; HRMS [M+H]^+^ calculated for [C_21_H_23_N_2_OS]^+^: 351.1526; found, 351.1527.

*(Z)-4-(2-((2-(3-acetamidopropanamido)ethyl)thio)-2-(naphthalen-1-yl)vinyl)-1-methylpyridin-1-ium iodide* (**17**): To a stirred solution of **19** (100 mg, 0.238 mmol) in MeCN (1ml) and DCM (0.5 ml), MeI (19 µl, 0.305 mmol) was added and the reaction was stirred for 5h at 50°C. The resulting solution was concentrated and re-dissolved in a small amount of DCM and triturated as an oil with Et_2_O. The resulting oil was collected and dried under vacuum, which gave **17** (90 mg, 0.674 mmol) as a yellow solid in 67% yield ;^1^H NMR (500 MH*z*, CDCl_3_): δ 8.98 (d, *J*=6.9 H*z*, 2H), 8.33 (d, *J*=6.9 H*z*, 2H), 8.12-8.08 (m, 1H), 7.91-7.86 (m, 2H), 7.55-7.45 (m, 5H), 7.06 (apparent t, *J*=5.6 H*z*, 1H), 6.61 (s, 1H), 4.51 (s, 3H), 3.43 (q, *J*=6.0 H*z*, 2H), 3.16 (q, *J*=5.6 H*z*, 2H), 2.56-2.46 (m, 4H), 1.93 (s, 3H); ^13^C NMR (125 MH*z*, CDCl_3_): δ 172.0, 170.8, 156.7, 152.1, 144.5, 135.8, 133.6, 130.6, 130.2, 128.8, 127.5, 126.9, 126.8, 125.4, 124.6, 123.6, 48.3, 38.4, 36.1, 35.9, 33.9, 23.6; HRMS [M+H]^+^ calculated for [C_25_H_28_N_3_O_2_S] ^+^: 434.1897; found, 434.190.

*(Z)-4-(2-((2-acetamidoethyl)thio)-2-(2,3-dichlorophenyl)vinyl)-1-methylpyridin-1-ium iodide* (**18**). **20** (30 mg, 0.081 mmol) was dissolved in acetonitrile (0.35 ml) and DCM (0.15 ml). CH_3_I (6.1 µl, 0.097 mmol) was added and the reaction was stirred for 5 h at 50°C. The resulting solution was concentrated and re-dissolved in a small amount of DCM and triturated as an oil with Et_2_O. The resulting oil was collected and dried under vacuum, which gave **18** (29 mg, 0.057 mmol) as a yellow powder in 70% yield. ^1^H NMR (500 MH*z*, CDCl_3_): δ 8.94 (d, *J*=7 H*z*, 2H), 8.32 (d, *J*=7 H*z*, 2H), 7.59-7.51 (m, 1H), 7.37-7.26 (m, 2H), 7.03 (apparent t, *J*=5.3 H*z*, 1H), 6.50 (s, 1H), 4.54 (s, 3H), 3.36 (q, *J*=6 H*z*, 2H), 2.70 (t, *J*=6 H*z*, 2H), 2.04 (s, 3H); ^13^C NMR (125 MH*z*, CDCl_3_): δ 171.0, 154.2, 151.6, 144.4, 139.-0, 134.3, 131.5, 130.6, 128.4, 128.0, 126.9, 123.3, 48.5, 38.5, 34.0, 23.5; HRMS [M+H]^+^ calculated for [C_18_H_19_Cl_2_N_2_OS] ^+^: 381.0590; found, 381.0586.

*(Z)-3-acetamido-N-(2-((1-(naphthalen-1-yl)-2-(pyridin-4-yl)vinyl)thio)ethyl)propanamide* **(19)**. (Z)-2-((1-(naphthalen-1-yl)-2-(pyridin-4-yl)vinyl)thio)ethan-1-amine (183 mg, 0.597 mmol) was dissolved in dry DMF (10 ml) and HATU (250 mg, 0.657 mmol), DIPEA (416 µl, 2.388 mmol), and 3-acetamidopropanoic acid (86.5 mg, 0.726 mmol) were added and the reaction was stirred at room temperature for 1h. The resulting mixture was diluted with DCM, and washed three times with water. Organic phase was dried with anhydrous Na_2_SO_4_(s), filtrated and concentrated. The resulting dark oil was purified using Flash chromatography over silica gel [DCM: MeOH 10:0-10:1] which gave **19** (200 mg, 0.477 mmol) in 80% yield; ^1^H NMR (500 MH*z*, CDCl_3_): δ 8.65 (d, *J*=6 H*z*, 2H), 8.26-8.21 (m, 1H), 7.93-7.87 (m, 2H), 7.58 (d, *J*=6 H*z*, 2H), 7.57-7.46 (m, 4H), 6.62 (s, 1H), 6.14 (broad s, 1H), 5.65 (broad s, 1H), 3.41 (q, *J*=6.0 H*z*, 2H), 3.11 (q, *J*=6.2 H*z*, 2H), 2.39 (t, *J*=6.2 H*z*, 2H), 2.23(t, *J*=6.0 H*z*, 2H), 1.89 (s, 3H); ^13^C NMR (125 MH*z*, CDCl_3_): δ 171.4, 170.2, 149.9, 143.6, 141.0, 136.8, 133.6, 131.3, 129.2, 128.6, 128.2, 126.9, 126.9, 126.5, 125.2, 125.1, 123.6, 38.8, 35.3, 35.3, 32.3, 23.3; HRMS [M+H]^+^ calculated for [C_24_H_26_N_3_O_2_S] ^+^: 420.1740; found, 420.1737.

*(Z)-N-(2-((1-(2,3-dichlorophenyl)-2-(pyridin-4-yl)vinyl)thio)ethyl)acetamide* (**20**). In a sealable tube, 4-((2,3-dichlorophenyl)ethynyl)pyridine (250 mg, 1.01 mmol) and Cs_2_CO_3_ (66 mg, 0.20 mmol) were mixed with EtOH (1 ml). N-Acetylcysteamine (180 mg, 1.51 mmol) dissolved in EtOH (1 ml) was added and the tube was capped and stirred overnight at 90°C. The resulting mixture was allowed to reach room temperature and was subsequently concentrated under reduced pressure. The resulting residue was purified by flash chromatography over silica gel [20:1 DCM:MeOH] followed by preparatory HPLC gave **20** (158 mg, 0.43 mmol) in 43% yield. ^1^H NMR (500 MH*z*, CDCl_3_): δ 8.63 (d, *J*=6.3 H*z*, 2H), 7.53-7.48 (m, 3 H), 7.34-7.26 (m, 2H), 6.51 (s, 1H), 5.80 (broad s, 1H), 3.27 (q, *J*=6.1 H*z*, 2H), 2.53 (t, *J*=6.1 H*z*, 2H), 1.90 (s, 3H); ^13^C NMR (125 MH*z*, CDCl_3_): δ 170.2, 150.0, 143.2, 140.4, 139.1, 134.1, 131.5, 130.7, 129.9, 129.3, 127.6, 123.8, 39.0, 32.6, 23.3; HRMS [M+H]^+^ calculated for [C_17_H_17_Cl_2_N_2_OS] ^+^: 367.0433; found, 367.0428.

*2-(naphthalen-1-yl)thieno[2,3-c]pyridine (***21***).* B-Thieno[2,3-c]pyridin-2-ylboronic acid (179 mg, 0.72 mmol), 1-bromonaphtalene (74 mg, 0.36 mmol), PdCl_2_DPPF (15 mg, 0.18 mmol), and Cs_2_CO_3_ (352 mg, 1.08 mmol) were mixed with dioxane (1ml) and H_2_O (0.1 ml). The reaction was stirred under Ar(g) at 80^o^C for 2 h. The resulting mixture was diluted with water and extracted with dichloromethane. The organic phase was washed two times with water, dried over anhydrous Na_2_SO_4_ (s), filtrated, and concentrated. The resulting oil was purified by flash chromatography over silica gel [heptane:EtOAC gradient] followed by flash chromatography over C18 stationary phase [H_2_O(0.1% formic acid):ACN (0.1% formic acid)] to give **21** (50 mg, 0.19 mmol) as a colorless solid in 53% yield. ^1^H NMR (500 MH*z*, CDCl_3_): δ 9.18 (s, 1H), 8.57 (d, *J*=5.4 H*z*, 1H), 8.22-8.18 (m, 1H), 7.98-7.92 (m, 2H), 7.77 (dd, *J*_1_=5.4Hz, *J*_2_=0.7 Hz, 1H), 7.67 (dd, *J*_1_=7.1Hz, *J*_2_=1.2 Hz, 1H), 7.58-7.51 (m, 3H), 7.48 (s, 1H); ^13^C NMR (125 MH*z*, CDCl_3_): δ 148.5, 145.4, 144.4, 143.6, 137.0, 133.9, 131.7, 131.5, 129.8, 128.8, 128.7, 127.1, 126.5, 125.4, 125.3, 123.1, 117.8; HRMS [M+H]^+^ calculated for [C_17_H_12_NS] ^+^: 262.0685; found, 262.0686.

*6-methyl-2-(naphthalen-1-yl)thieno[2,3-c]pyridin-6-ium (***22**). Methyl iodide (167 µl, 5 eq) was added to **21** (140 mg, 1 eq) in CH_3_CN (2 ml) and CHCl_3_ (4 ml). The reaction was capped and stirred at 50°C for 2 h before being purified by HPLC, which gave **22** (6.1 mg. 15 µmol) as a glass in 28% isolated yield.  ^1^H NMR (500 MHz, CDCl_3_+CD_3_OD) δ 8.57 (d, *J* = 6.7 Hz, 1H), 8.22 (d, *J* = 6.7 Hz, 1H), 8.14 – 8.07 (m, 1H), 8.02 (dt, *J* = 8.3, 1.1 Hz, 1H), 7.99 – 7.93 (m, 1H), 7.79 (s, 1H), 7.69 (dd, *J* = 7.2, 1.2 Hz, 1H), 7.62 – 7.55 (m, 3H), 7.27 (s, 1H), 4.61 (s, 3H). ^13^C NMR (126 MHz, CDCl_3_+CD_3_OD) δ 169.09, 161.56, 149.31, 141.40, 137.88, 137.49, 133.92, 131.63, 130.69, 129.53, 129.14, 129.01, 128.04, 127.04, 125.34, 124.39, 123.05, 120.54, 29.76. HRMS [M]^+^ calculated for [C_18_H_14_NS]^+^: 276.0841; 276.0843.

*Tert-butyl 2-(naphthalen-1-yl)-4,7-dihydrothieno[2,3-c]pyridine-6(5H)-carboxylate*. Tert-butyl 4,7-dihydrothieno[2,3-c]pyridine-6(5H)-carboxylate (239 mg, 1.0 mmol), PdCl(C_3_H_5_)(dppb) (12 mg, 0.02 mmol), and KOAc (196 mg, 2.0 mmol) were dissolved in DMA (2 ml) followed by addition of 1-bromonaphthalene (280 µl, 2.0 mmol). The reaction was sealed and stirred overnight at 155^o^C. The resulting mixture was purified by flash chromatography over silica gel [8:1 heptane:EtOAc] which gave the product (312 mg, 0.854 mmol) as a yellow sticky oil in 85% yield. ^1^H NMR (500 MH*z*, CDCl_3_): δ 8.29-8.23 (m, 1H), 7.91-7.87 (m, 1H), 7.85 (d, J=8 Hz, 1H), 7.56-7.46 (m, 4H), 6.94 (s, 1H), 2.17 (broad s, 2H), 3.81-3-71 (m, 2H), 2.85-2.75 (m, 2H), 9.03 (s, 9H); ^13^C NMR (125 MH*z*, CDCl_3_): δ 154.8, 139.4, 133.9, 132.4, 131.8, 128.4, 128.4, 128.0, 127.4, 126.5, 126.1, 125.8, 125.3, 80.2, 43.3 (apparent broad d), 41.4 (apparent broad d), 28.6, 25.8 ; HRMS [M+H]^+^ calculated for [C_22_H_24_NO_2_S] ^+^: 366.1517; found, 366.1523.

*2-(naphthalen-1-yl)-4,5,6,7-tetrahydrothieno[2,3-c]pyridine* (**23**). Tert-butyl 2-(naphthalen-1-yl)-4,7-dihydrothieno[2,3-c]pyridine-6(5H)-carboxylate (120 mg, 0.328 mmol) was dissolved in DCM (0.5 ml) and TFA (0.25 ml, 3.28 mmol) and stirred for two hours at room temperature. The resulting greenish solution was diluted with DCM and washed three times with NaHCO_3_ (aq., 1 M) and brine. The organic phase was dried over anhydrous Na_2_SO_4_ (s), filtrated, and concentrated which gave **23** (82 mg, 0.309 mmol) as a yellow oil in 94 % yield. ^1^H NMR (500 MH*z*, CDCl_3_): δ 8.32-8.25 (m, 1H), 7.91-7.87 (m, 1H), 7.85-7.81 (m, 1H), 7.56-7.45 (m, 4H), 6.93 (s, 1H), 4.12-4.10 (m, 2H), 3.20 (t, *J*=5.8 H*z*, 2H), 2.76-2.71 (m, 2H), 1.67(broad s, 1H); ^13^C NMR (125 MH*z*, CDCl_3_): δ 138.5, 135.2, 134.3, 134.0, 132.9, 131.9, 128.5, 128.3, 128.0, 126.5, 126.1, 126.0, 125.4, 45.2, 43.7, 27.0; HRMS [M+H]^+^ calculated for [C_17_H_16_NS] ^+^: 266.0998; found, 266.0995.

*6-methyl-2-(naphthalen-1-yl)-4,5,6,7-tetrahydrothieno[2,3-c]pyridin-6-ium chloride* (**24**). Tert-butyl 2-(naphthalen-1-yl)-4,7-dihydrothieno[2,3-c]pyridine-6(5H)-carboxylate (33 mg, 0.091 mmol) was dissolved in dry THF (1 ml) followed by addition of LAH (7.5 mg). After stirring at 40^o^C overnight, the reaction was quenched with water and extracted twice with Et_2_O. The combined organic phases were washed with brine, dried over Na_2_SO_4_, filtrated, and concentrated. The resulting residue was converted to the HCl salt using HCl in Et_2_O and purified by trituration with Et_2_O which gave **24** (25 mg, 0.079 mmol) as the hydrochloric salt in 87% yield. ^1^H NMR (500 MH*z*, DMSO-d6): δ 10.95 (broad s, 1H), 8.20-8.14 (m, 1H), 8.06-7.98 (m, 2H), 7.63-7.55 (m, 4H), 7.20 (s, 1H), 4.73-4.64 (m, 1H), 4.46-4.36 (m, 1H), 3.75-3.66 (m, 2H), 3.47-3.37 (m, 1H), 3.17-3.00 (m, 2H); ^13^C NMR (125 MH*z*, DMSO-d6): δ140.4, 134.0, 133.0, 131.4, 131.2, 129.3, 129.1, 128.4, 127.8, 127.5, 127.3, 126.8, 126.0, 125.2; HRMS [M+H]^+^ calculated for [C_18_H_18_NS] ^+^: 280.1154; found, 280.1152.

### Supporting data S2- NMR Data

*S-Ethyl Coenzyme A (****S-ethyl-CoA****).*

**
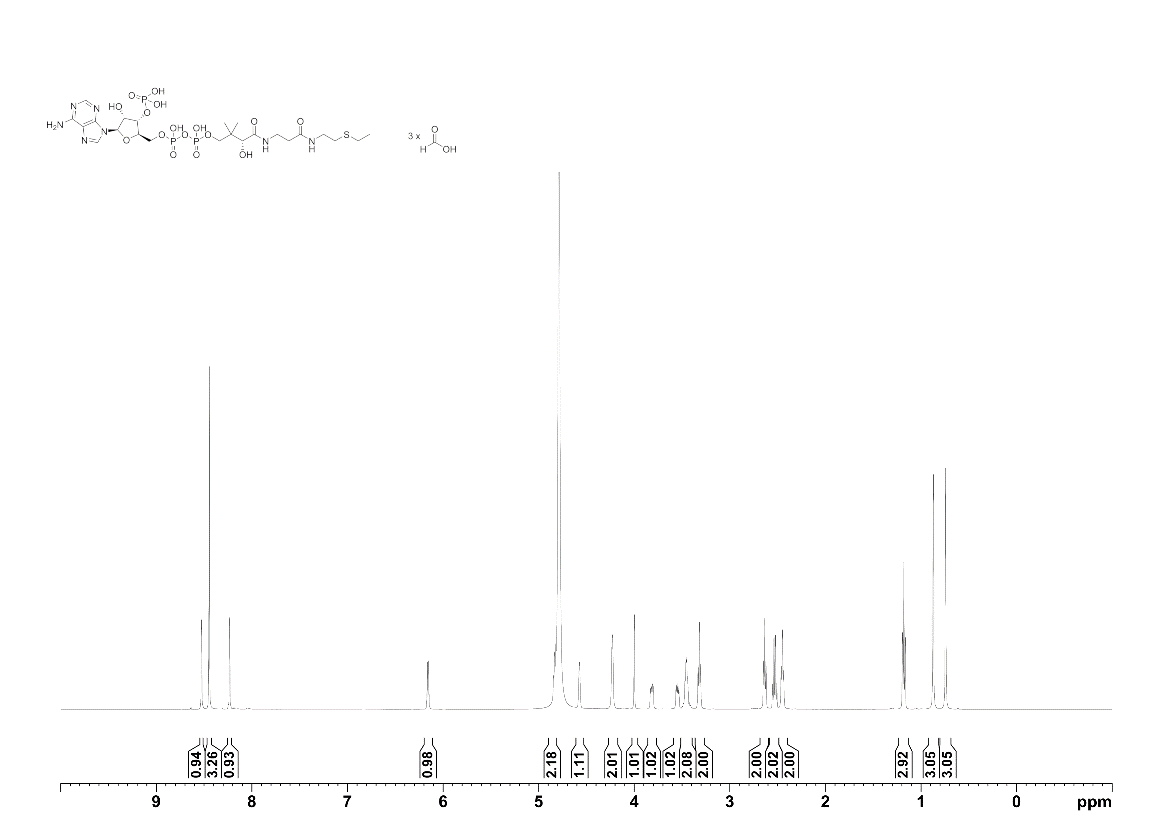
**


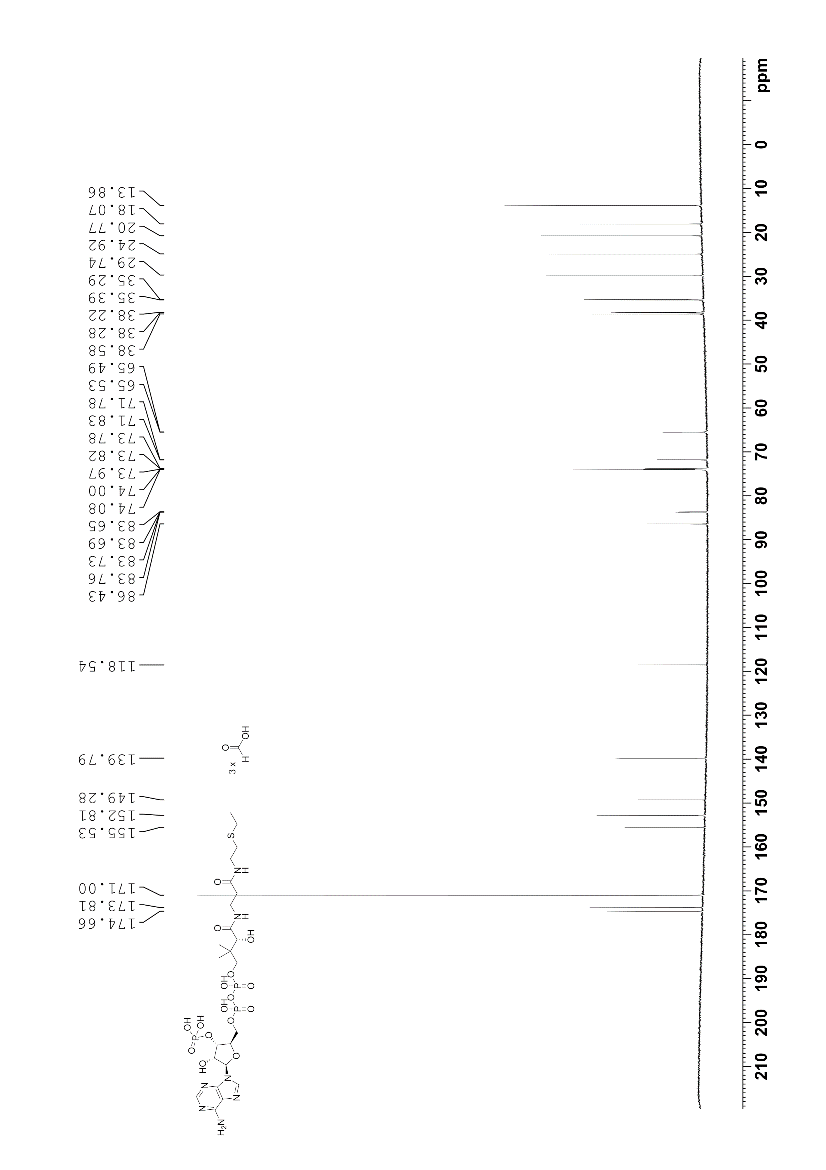


*1-methyl-4-[2-(naphthalen-1-yl)ethyl]pyridin-1-ium (****6****)*

**
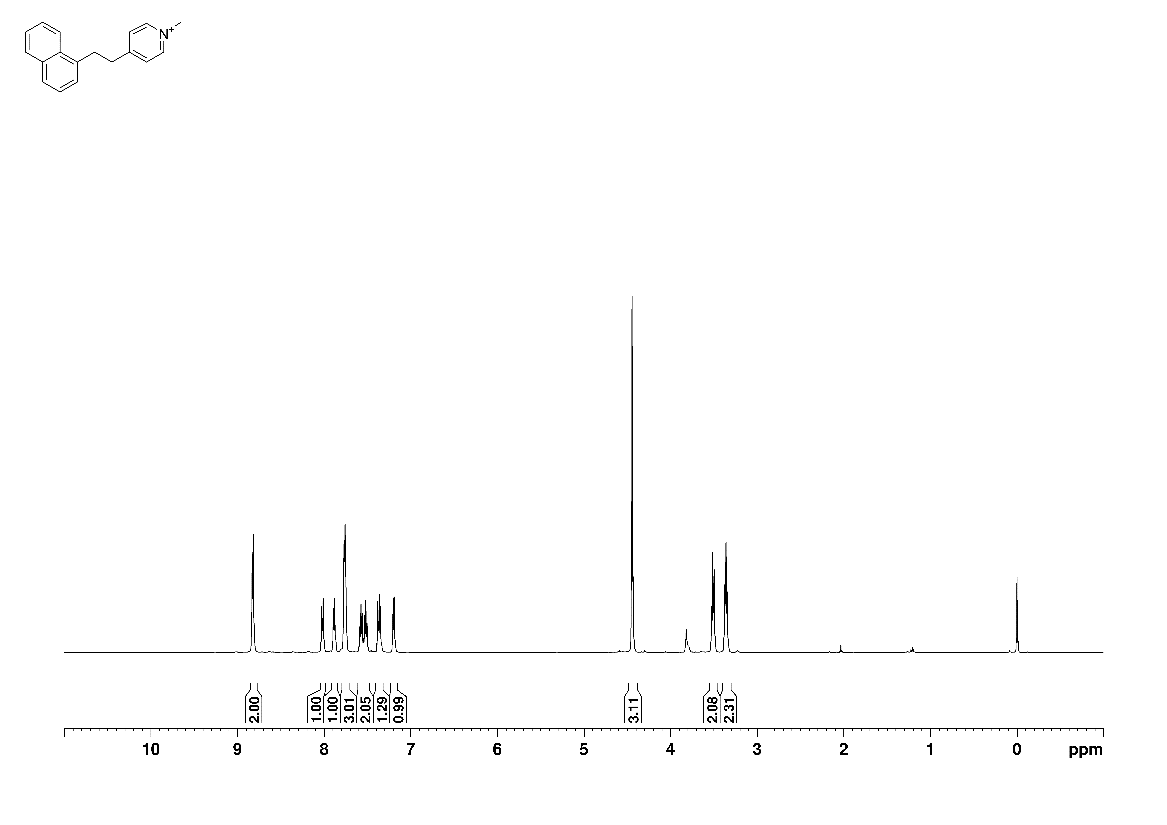
**

**
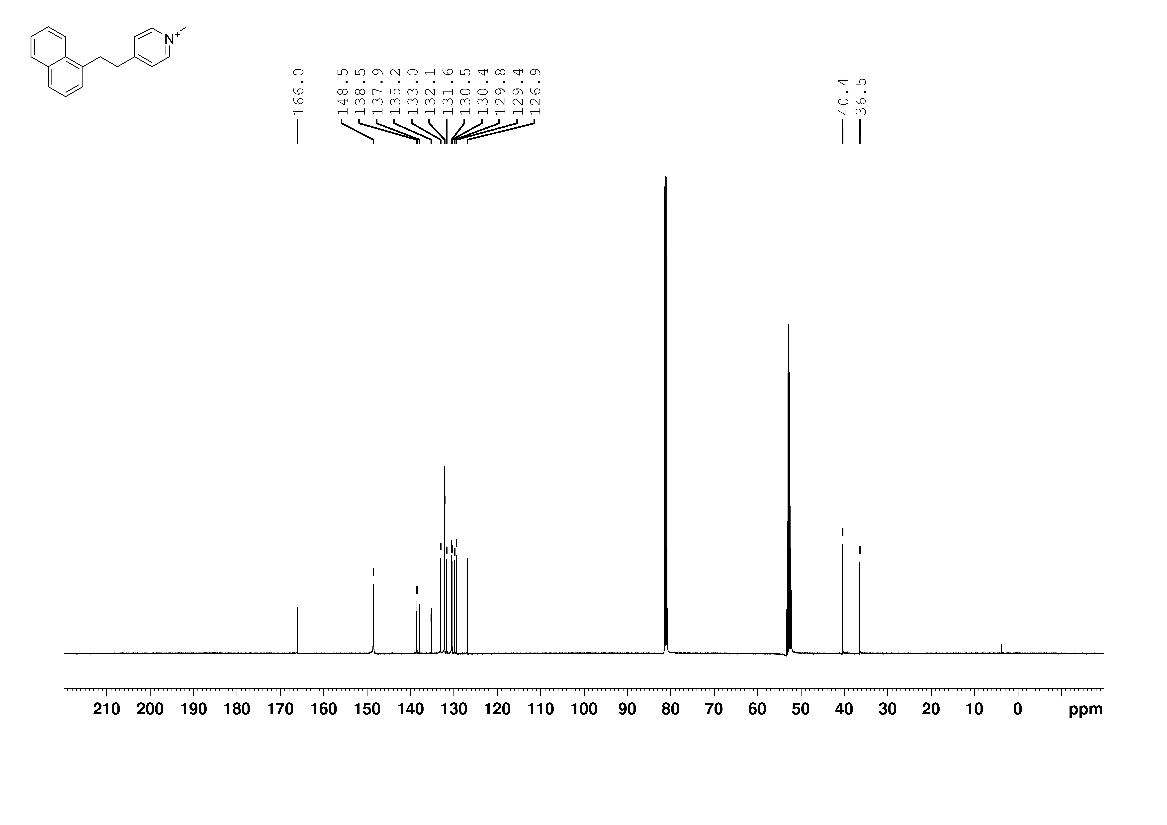
**

*(Z)-N-(2-((1-(naphthalen-1-yl)-2-(pyridin-4-yl)vinyl)thio)ethyl)acetamide (****7****)*

**
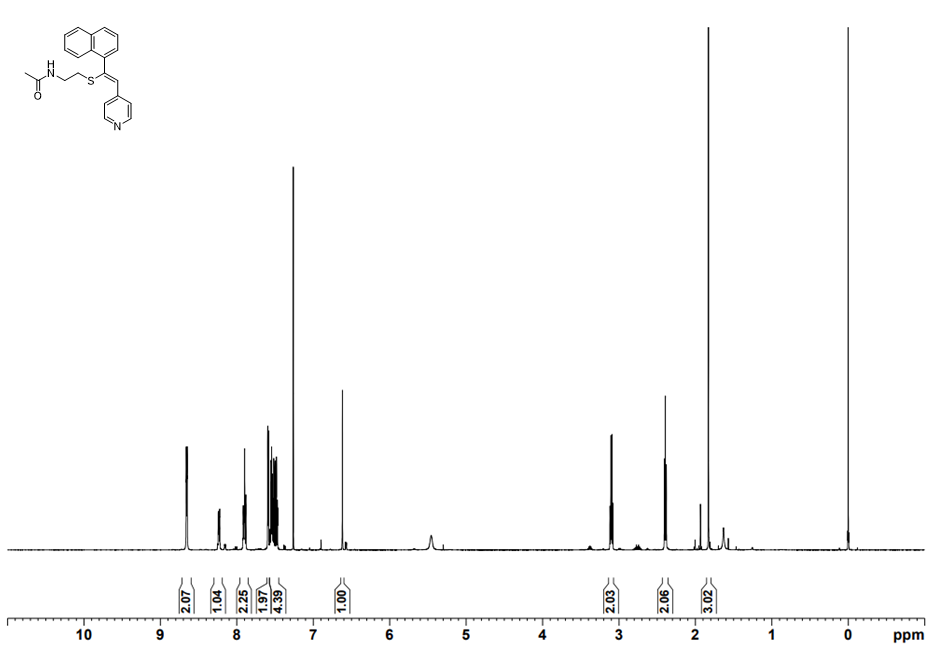
**

**
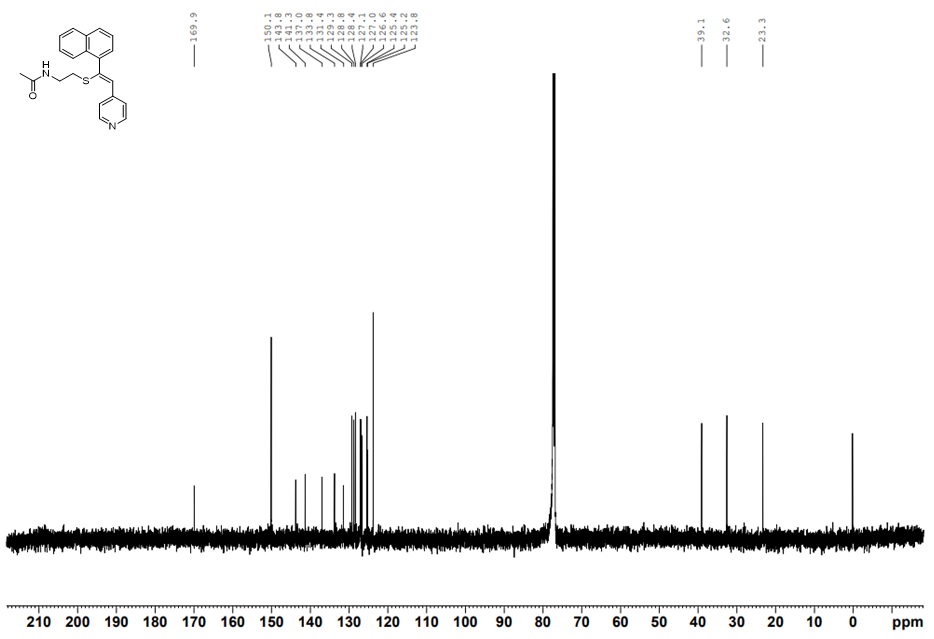
**

*1-methyl-4-[2-(naphthalen-1-yl)-2-(propylsulfanyl)ethyl]pyridin-1-ium (****8****)*

**
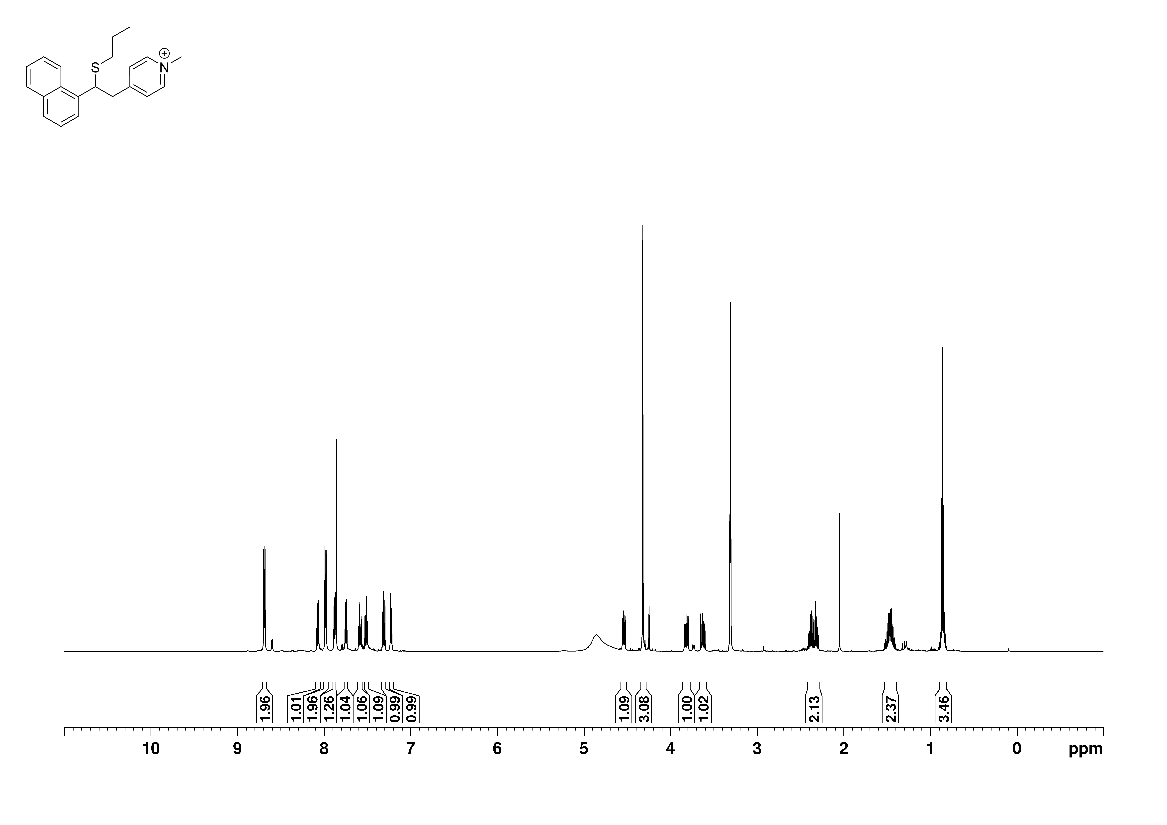
**

**
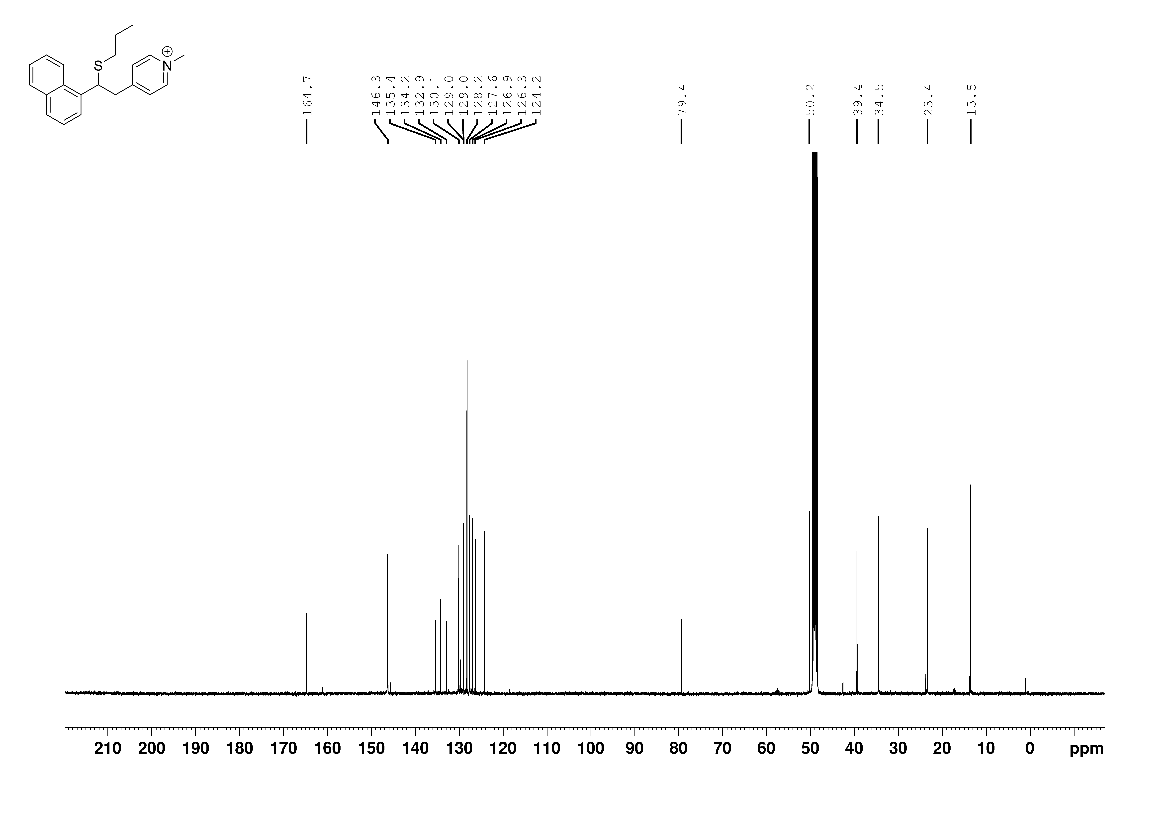
**

*(Z)-4-(2-((2-acetamidoethyl)thio)-2-(naphthalen-1-yl)vinyl)-1-methylpyridin-1-ium iodide (****9****)*

**
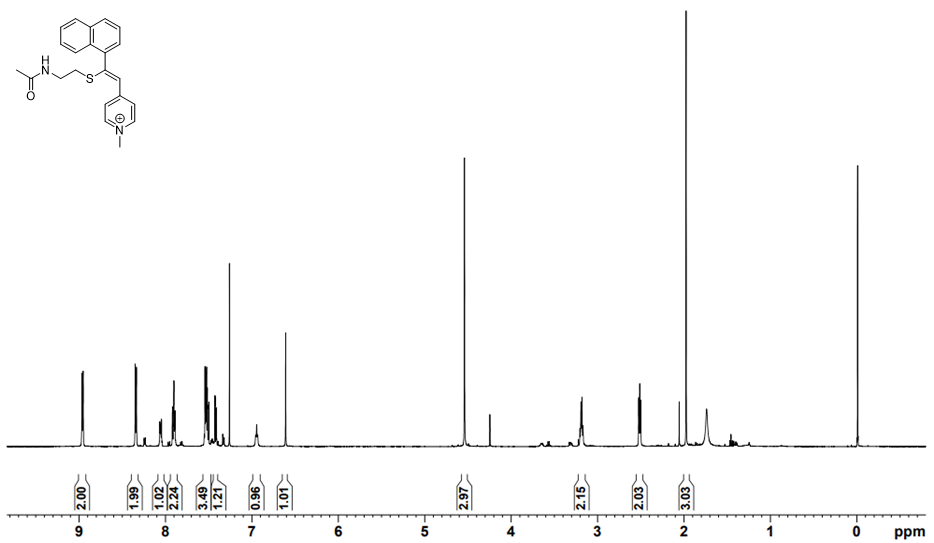
**

**
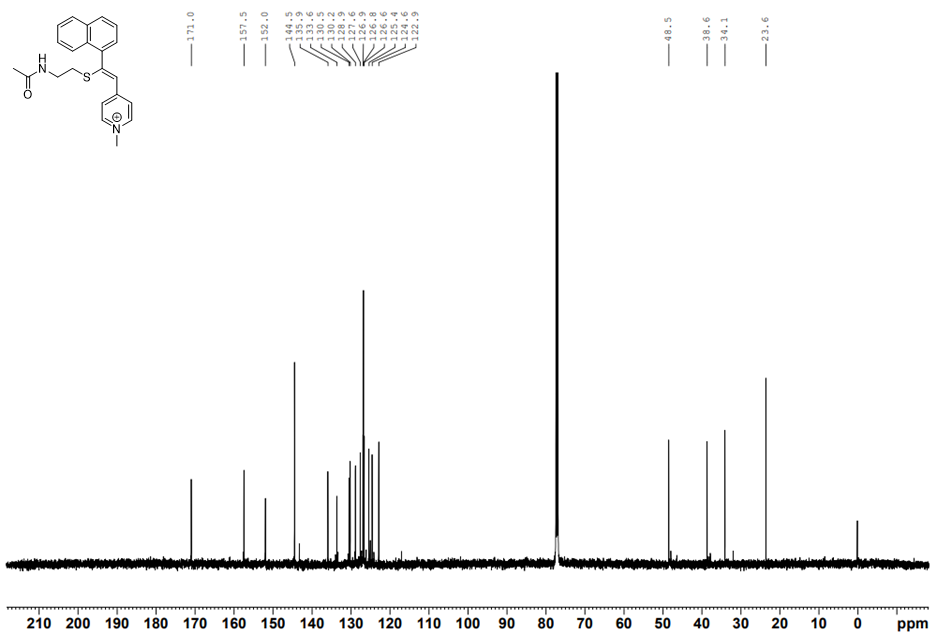
**

*(Z)-4-(2-((2-acetamidoethyl)thio)-2-(naphthalen-1-yl)vinyl)-1-benzylpyridin-1-ium bromide (****10****)*

**
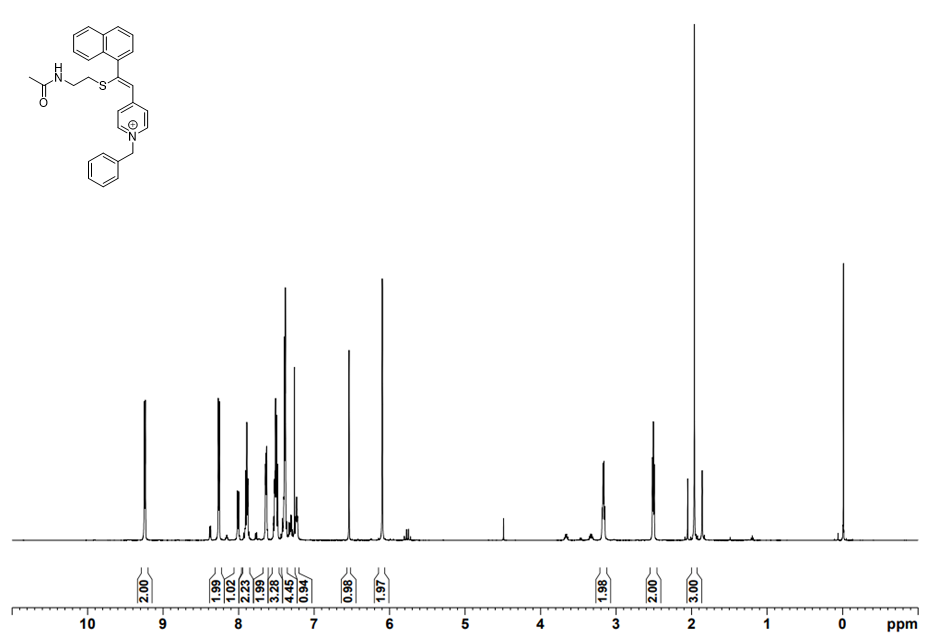
**

**
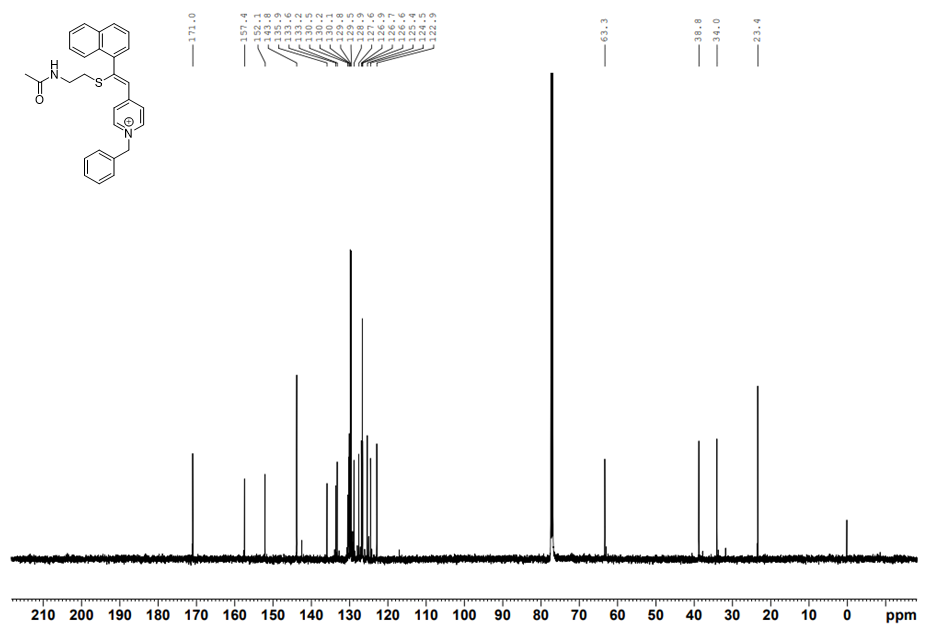
**

*6-benzyl-2-(naphthalen-1-yl)thieno[2,3-c]pyridin-6-ium bromide (****11****)*


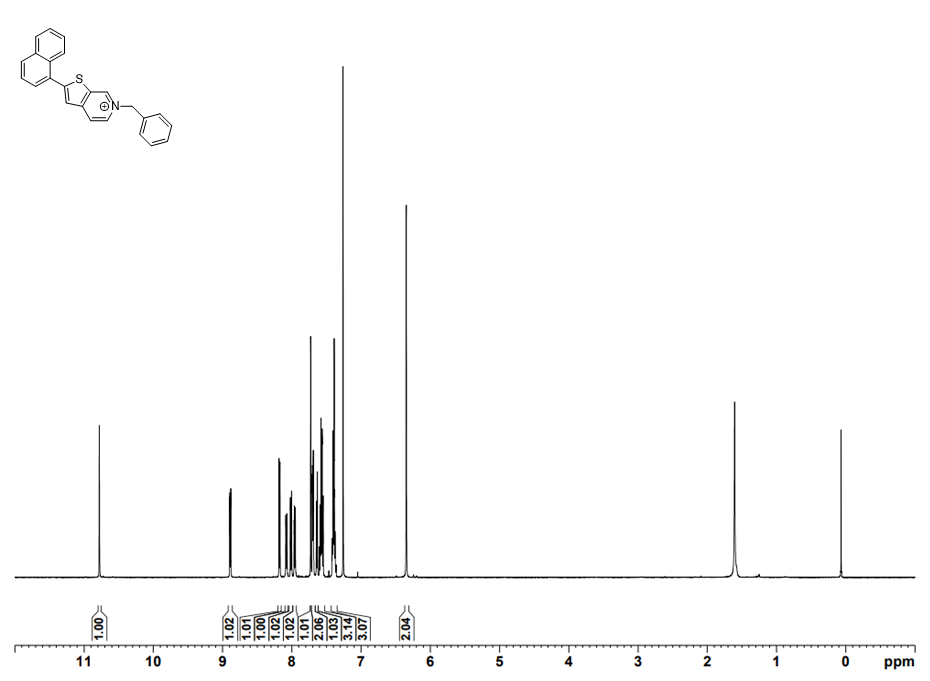


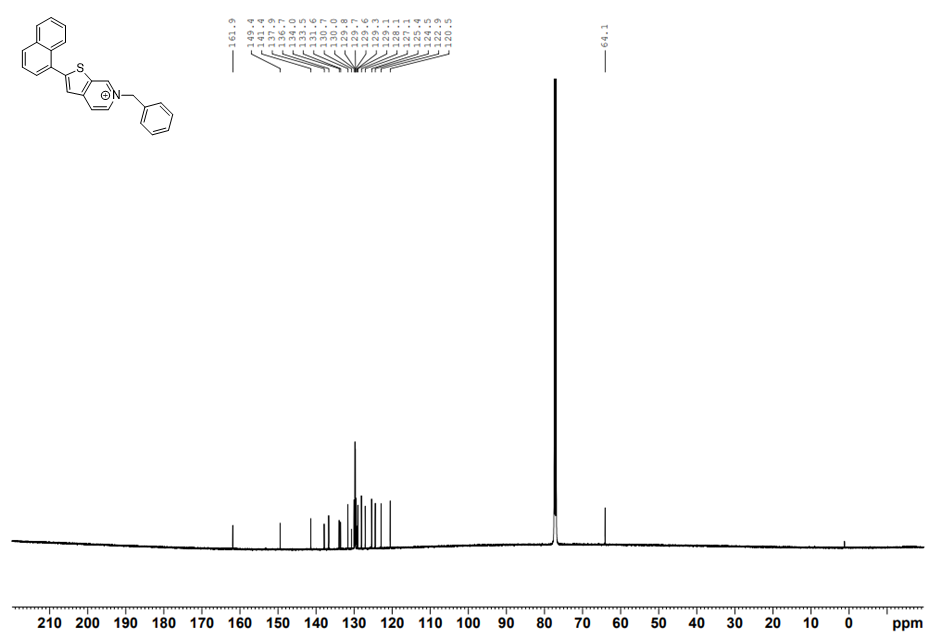


*4-[2-(naphthalen-1-yl)ethyl]pyridine (****12****)*


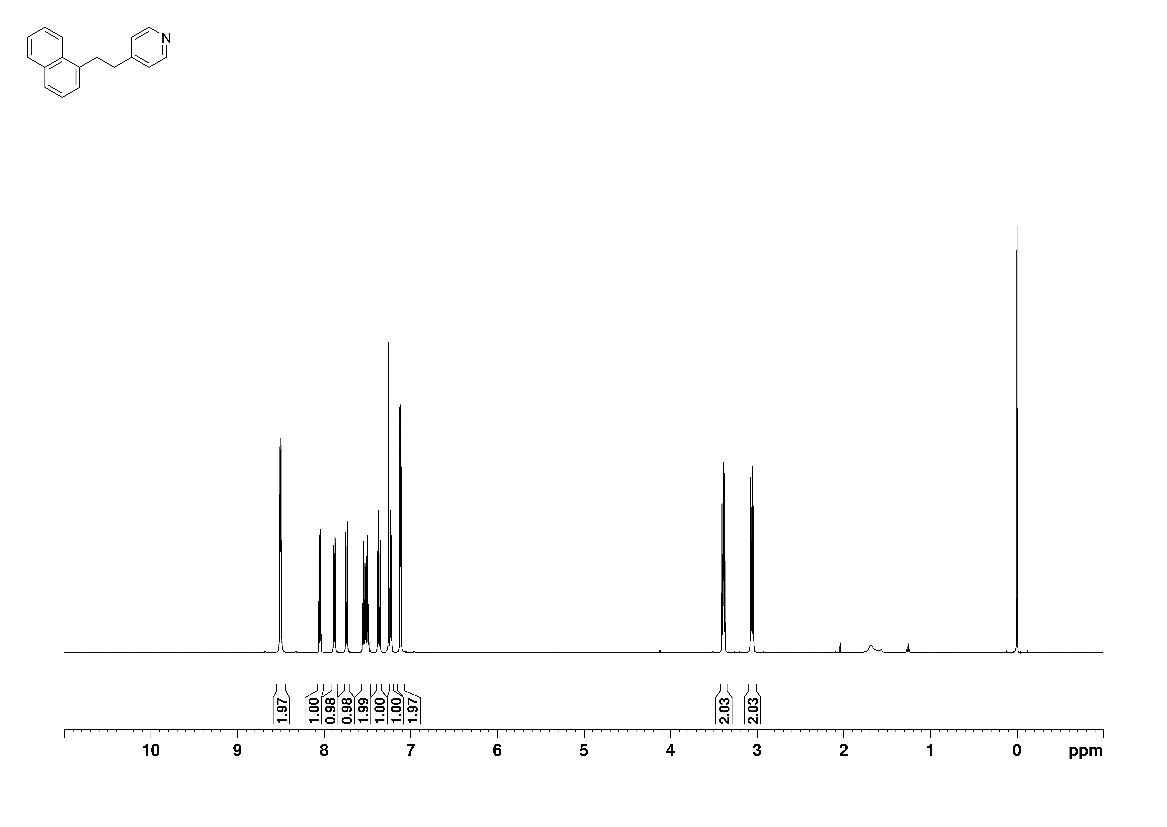


**
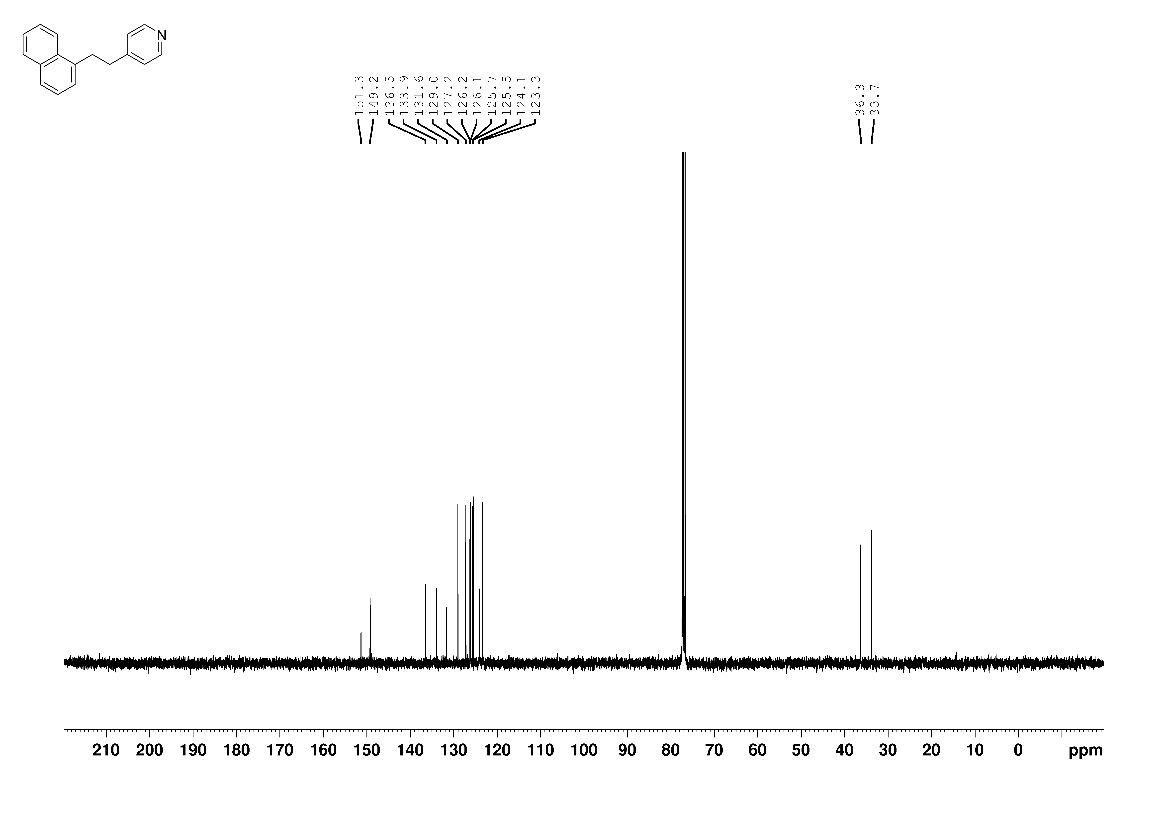
**

*4-[2-(naphthalen-1-yl)ethyl]-1-propylpyridin-1-ium* *(****13****)*

**
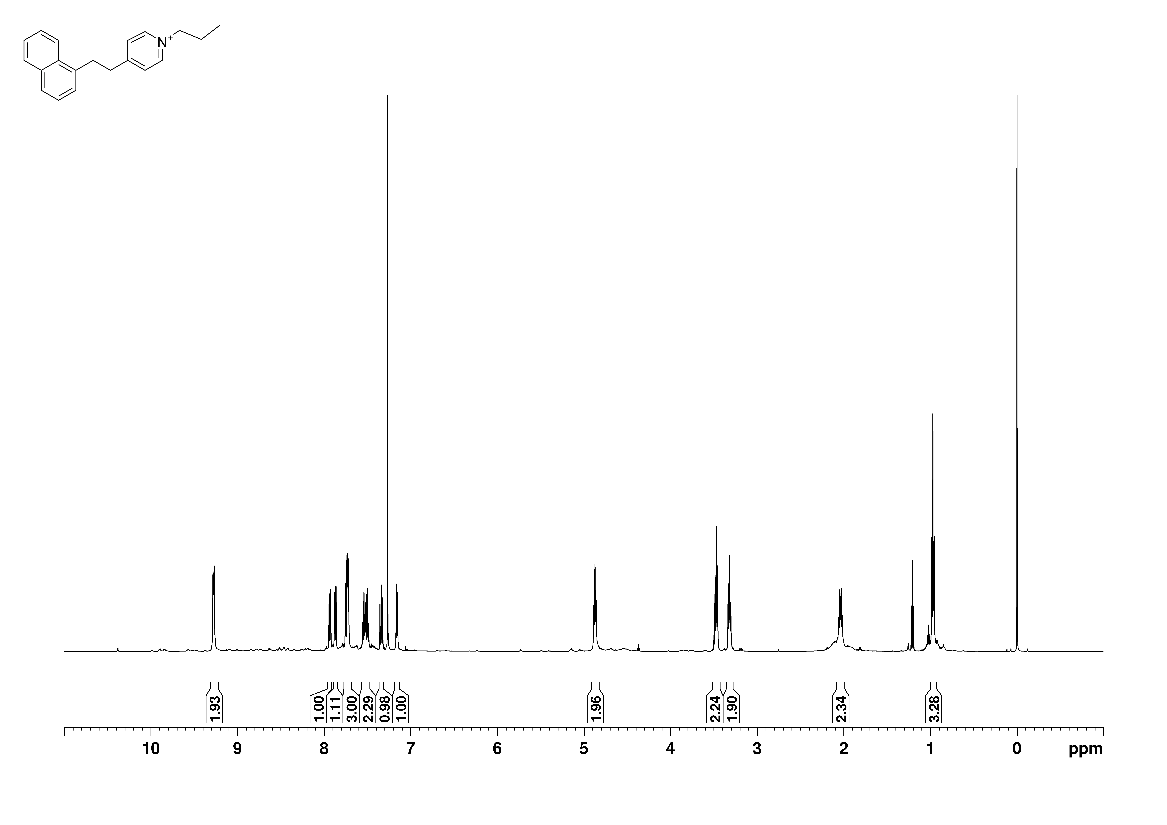
**

**
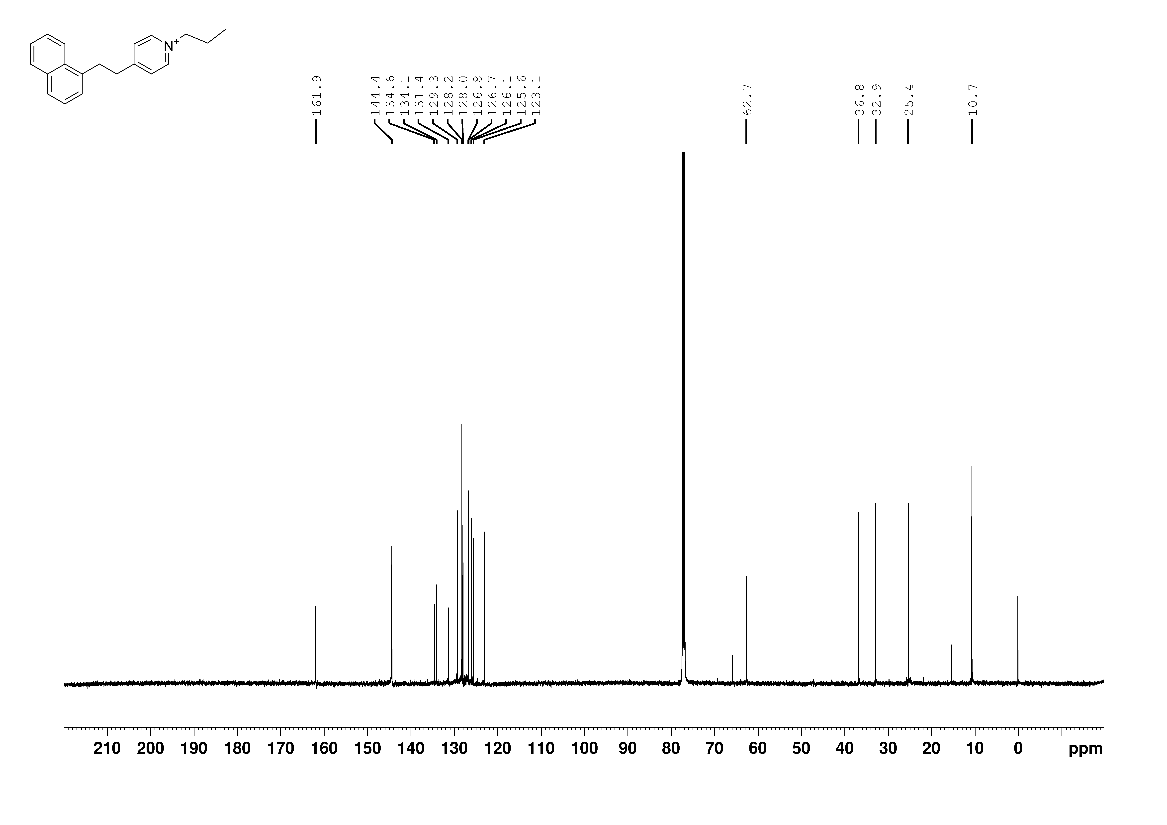
**

*1-benzyl-4-[2-(naphthalen-1-yl)ethyl]pyridin-1-ium (****14****)*

**
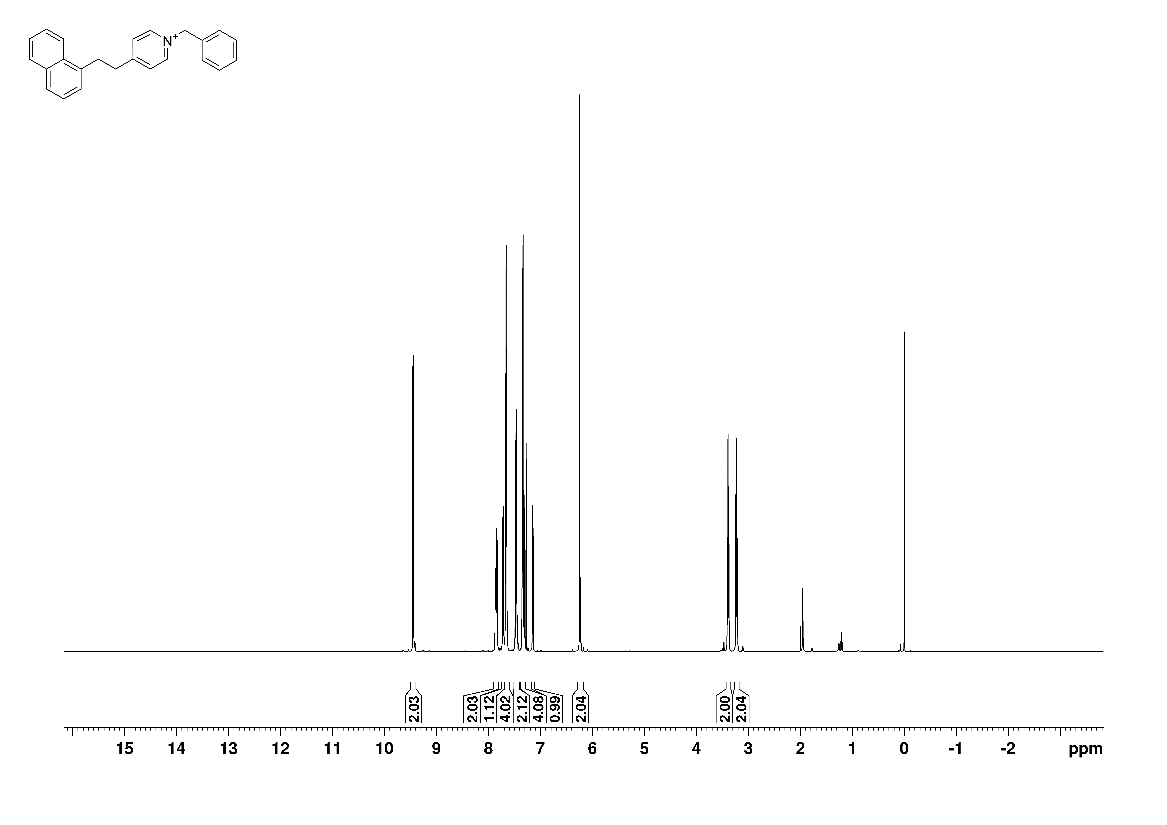
**

**
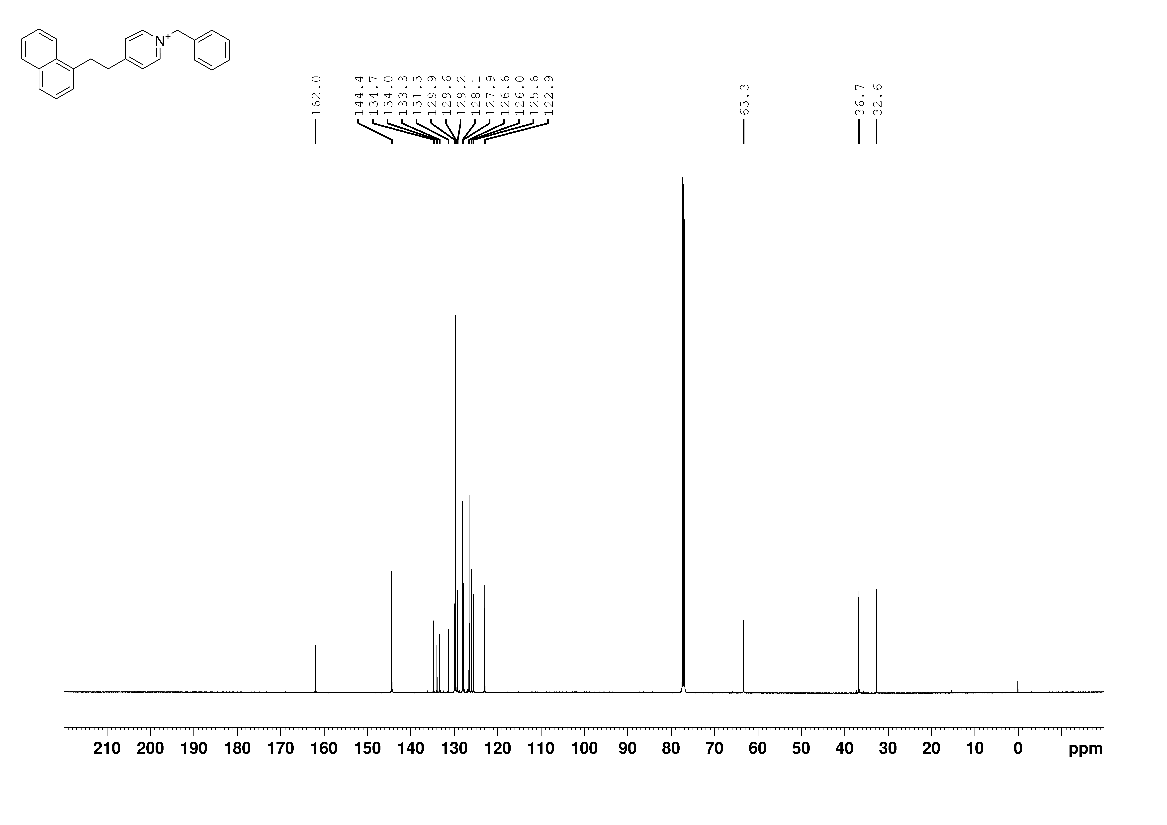
**

*4-[2-(3,4-dichlorophenyl)-2-(propylsulfanyl)ethyl]pyridine (****15****)*

**
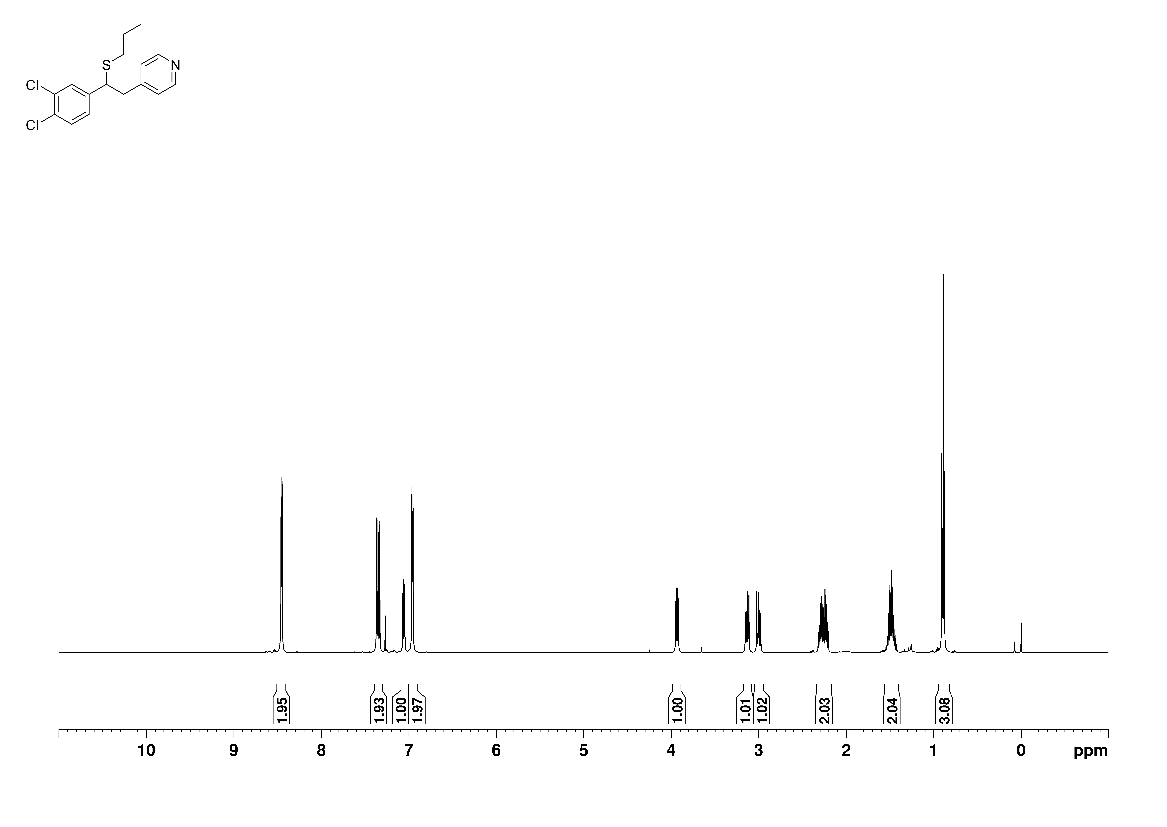
**

**
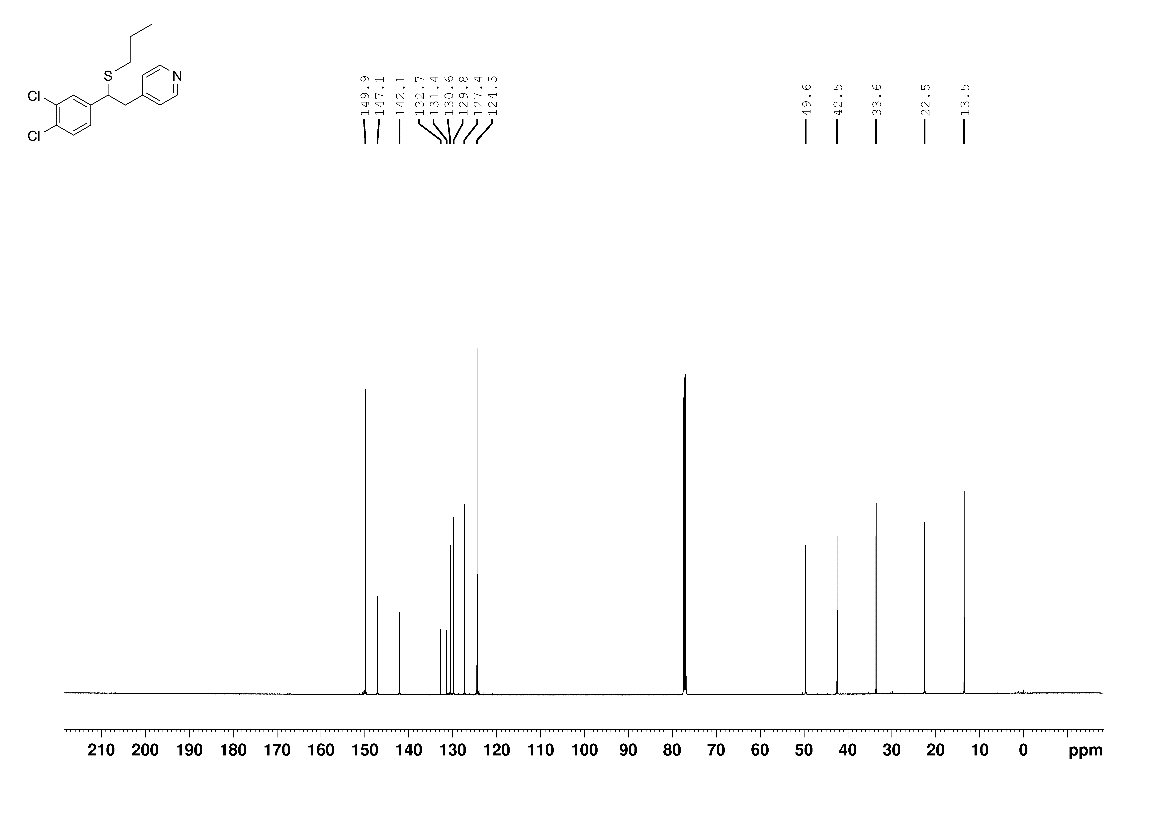
**

*N-(2-{[1-(naphthalen-1-yl)-2-(pyridin-4-yl)ethyl]sulfanyl}ethyl)acetamide* *(****16****)*

**
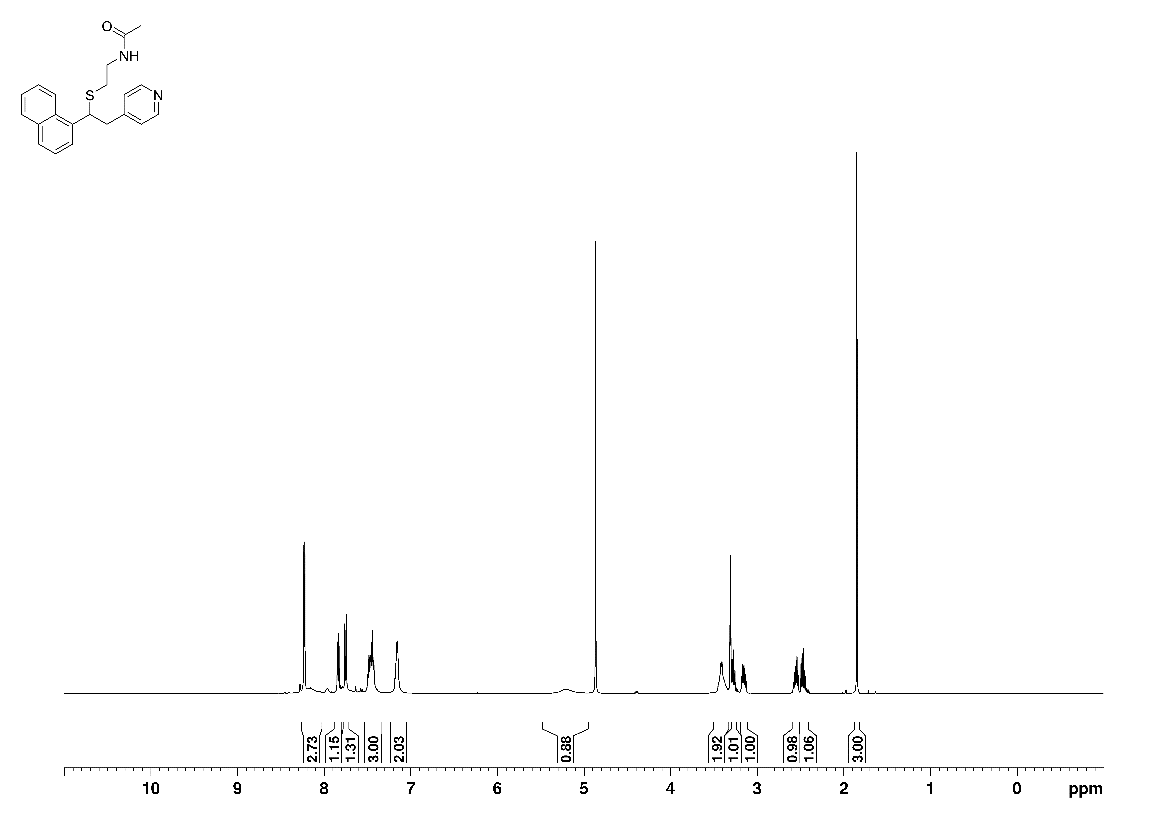
**

**
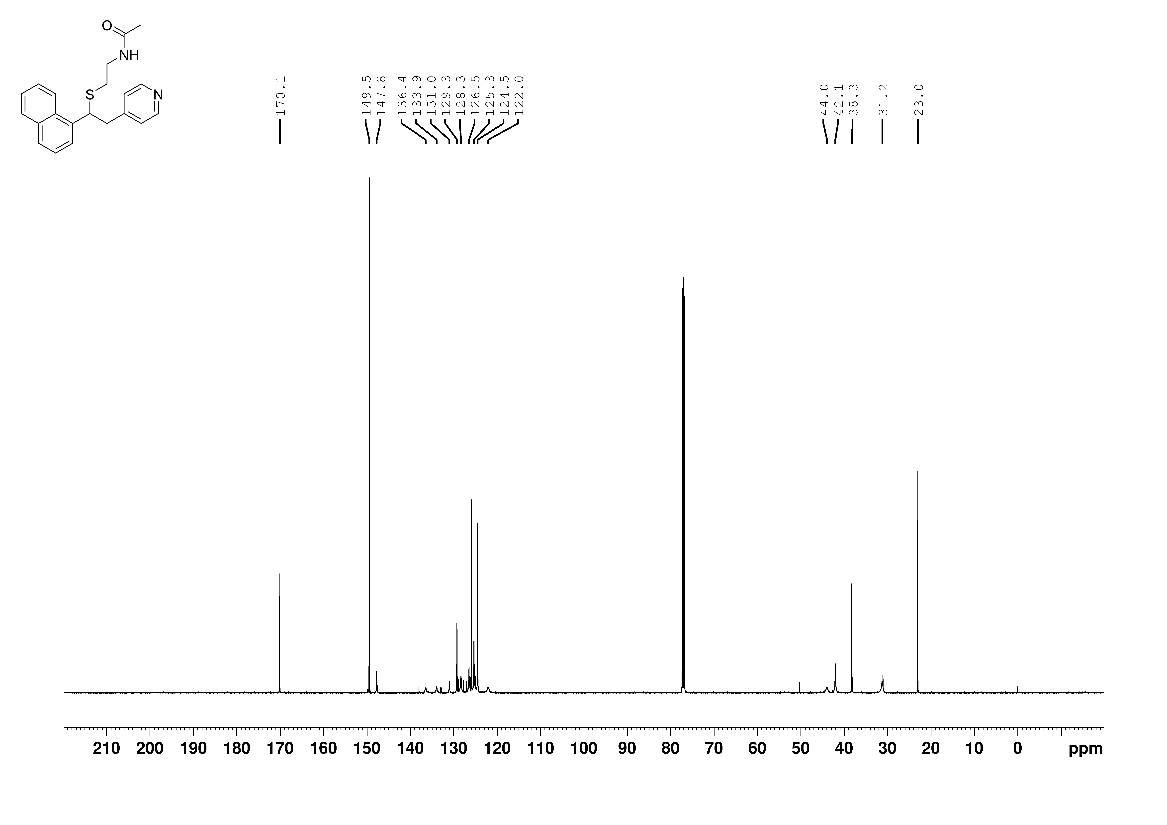
**

*(Z)-4-(2-((2-(3-acetamidopropanamido)ethyl)thio)-2-(naphthalen-1-yl)vinyl)-1-methylpyridin-1-ium iodide (****17****)*

**
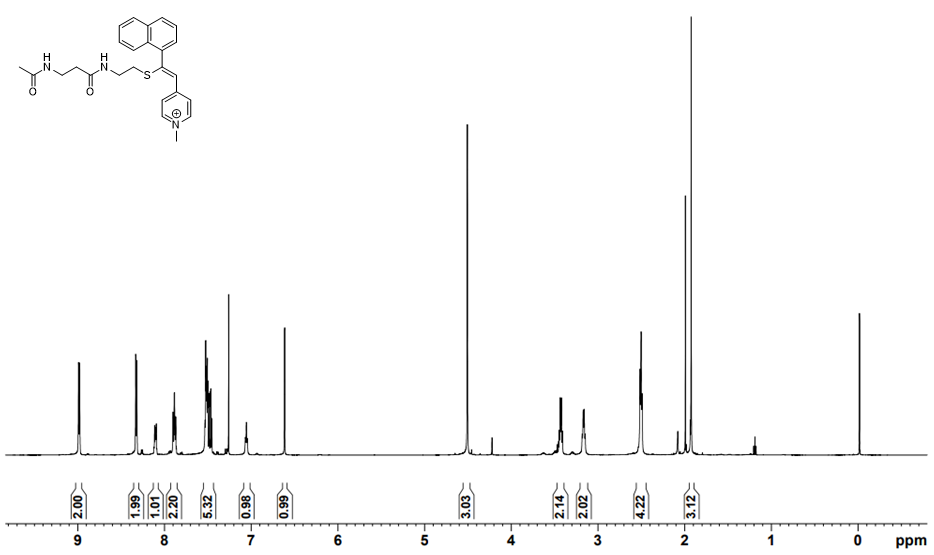
**

**
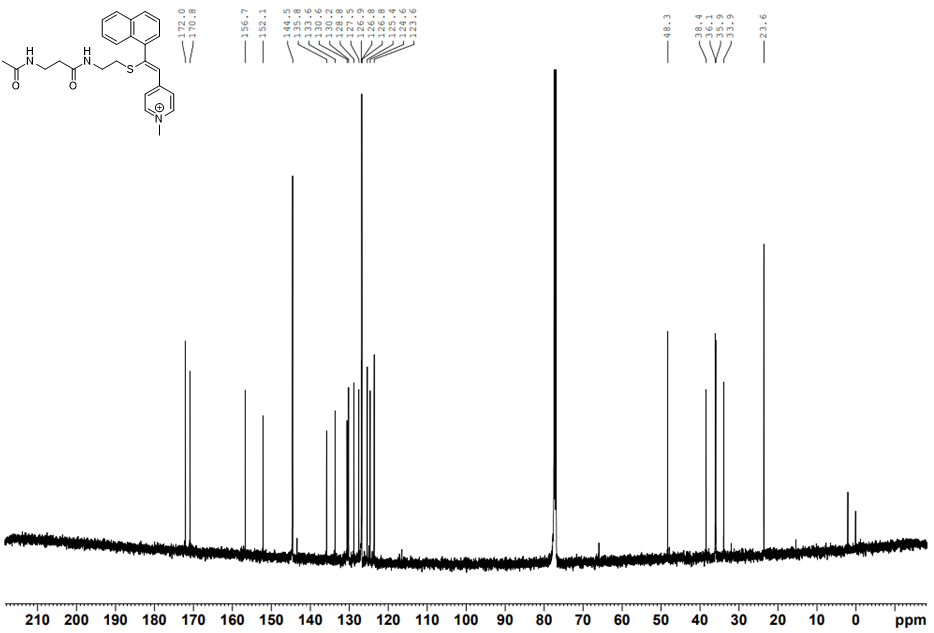
**

*(Z)-4-(2-((2-acetamidoethyl)thio)-2-(2,3-dichlorophenyl)vinyl)-1-methylpyridin-1-ium iodide (****18****)*


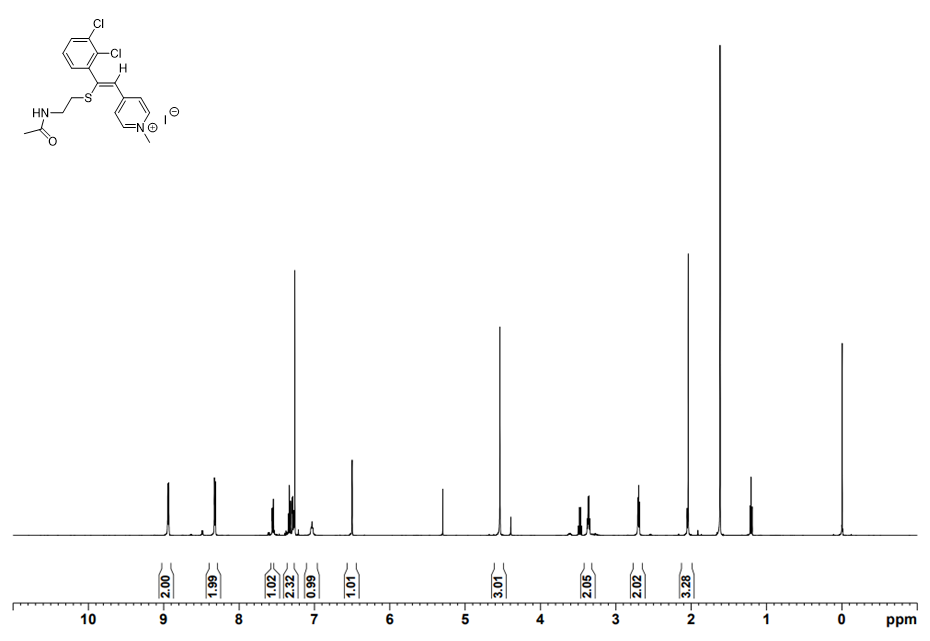


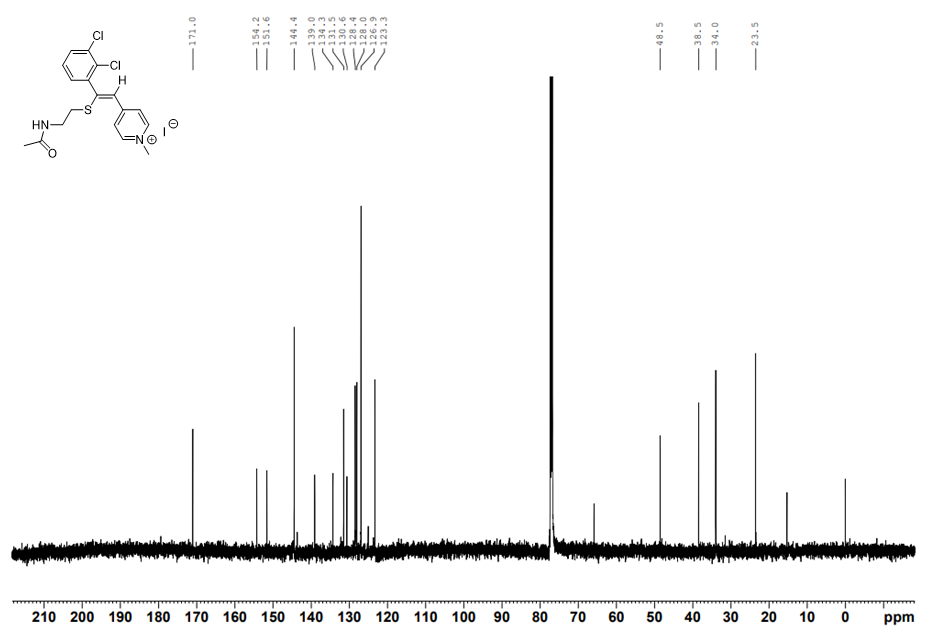


*(Z)-3-acetamido-N-(2-((1-(naphthalen-1-yl)-2-(pyridin-4-yl)vinyl)thio)ethyl)propanamide (****19****)*

**
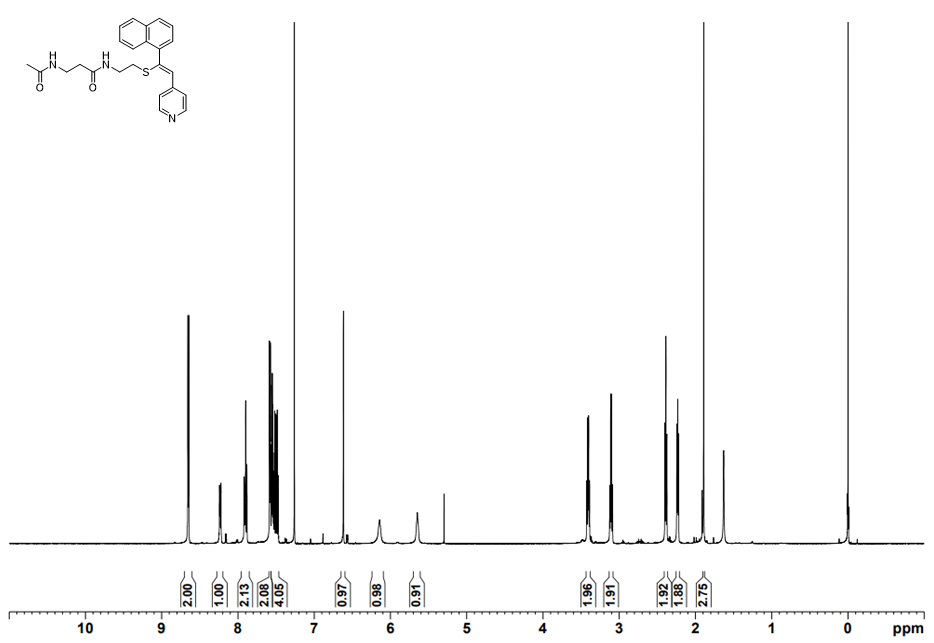
**

**
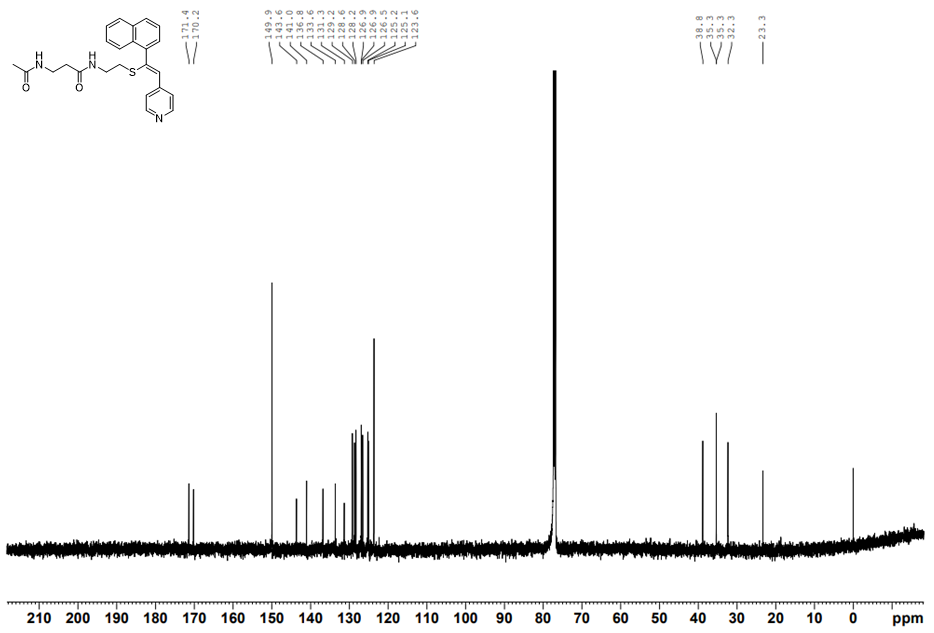
**

*(Z)-N-(2-((1-(2,3-dichlorophenyl)-2-(pyridin-4 yl)vinyl)thio)ethyl)acetamide (****20****)*


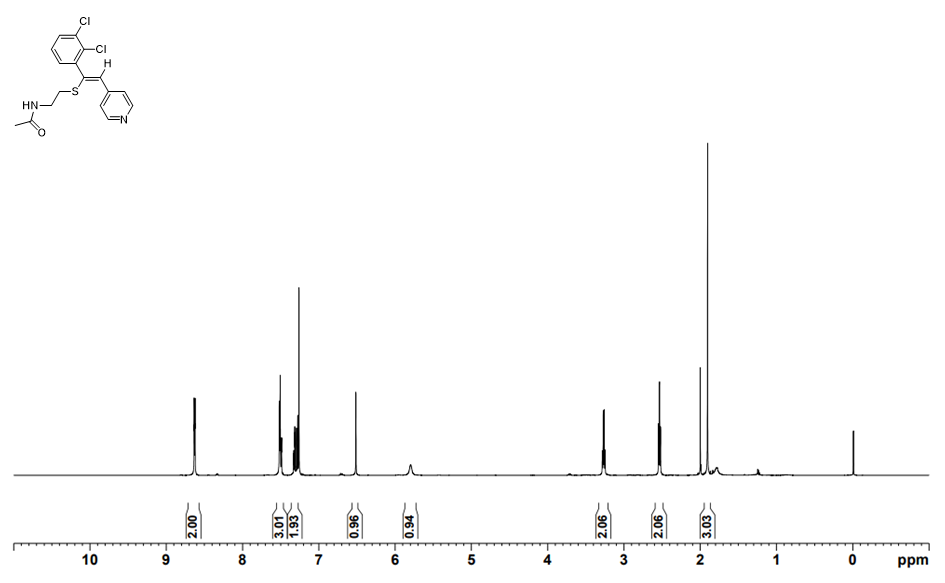


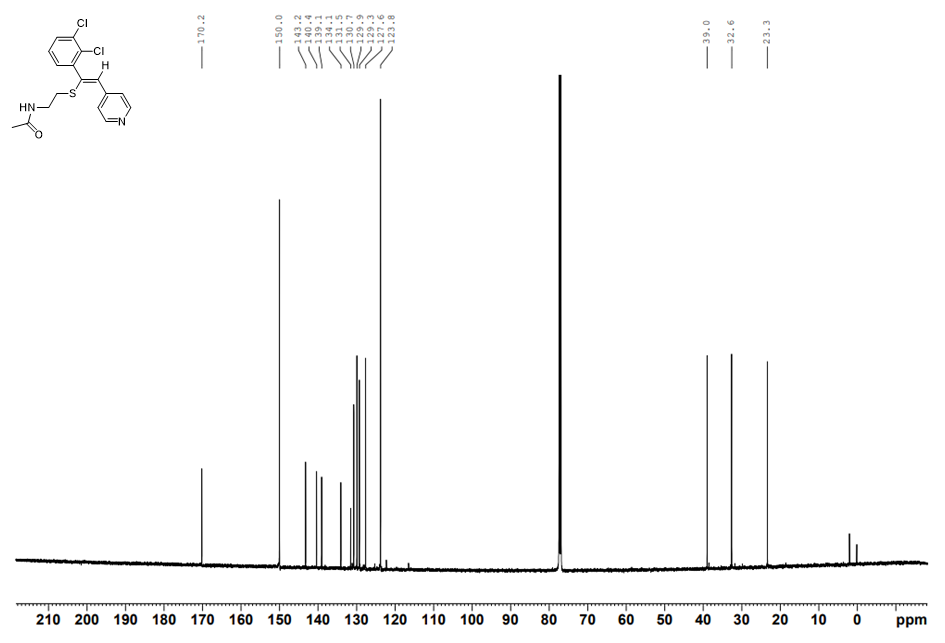


*2-(naphthalen-1-yl)thieno[2,3-c]pyridine (****21****)*

**
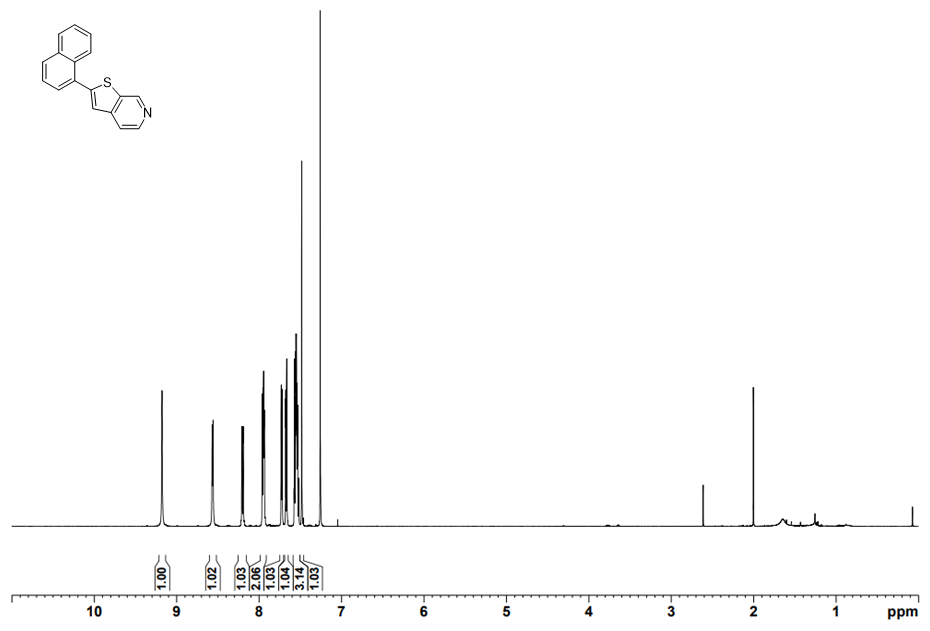
**

**
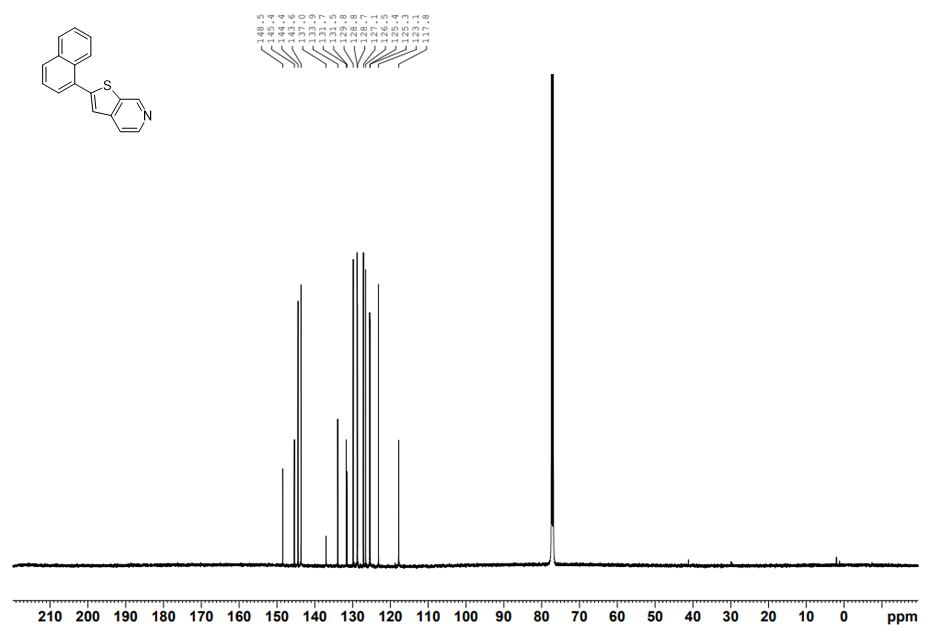
**

*6-methyl-2-(naphthalen-1-yl)thieno[2,3-c]pyridin-6-ium formate (****22****)*


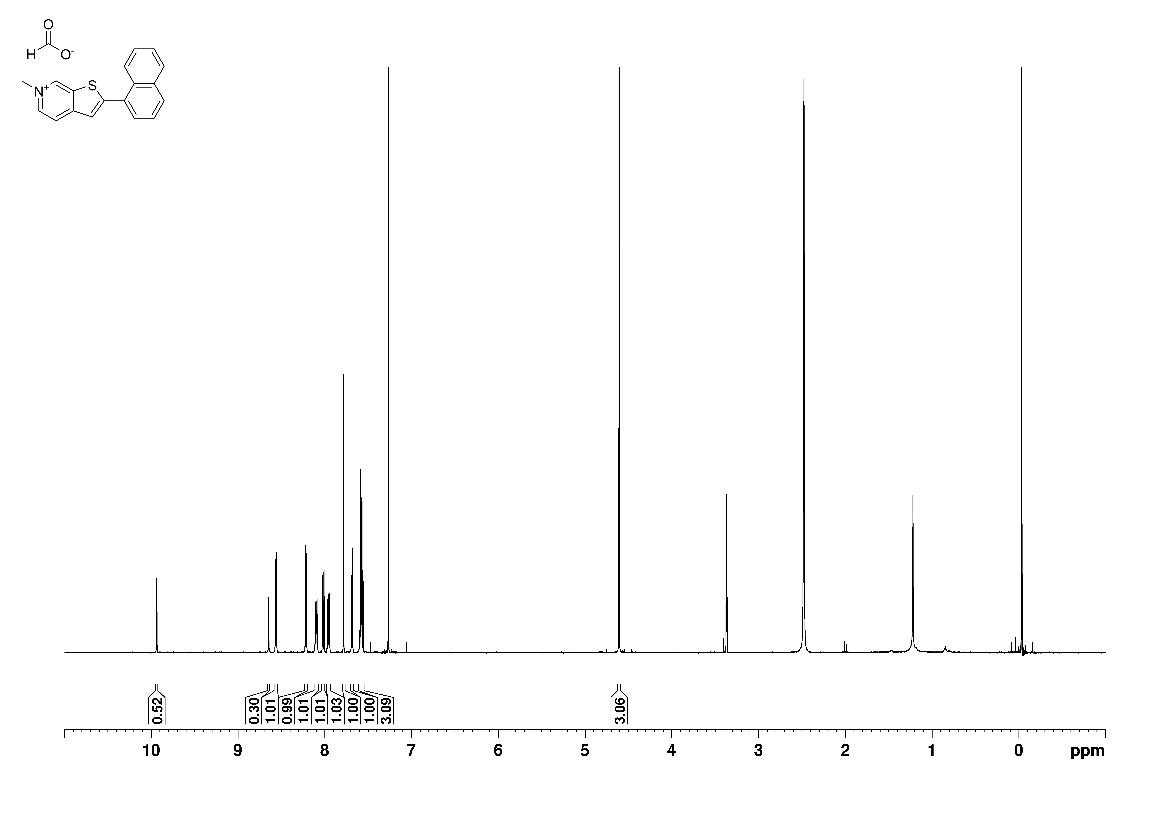


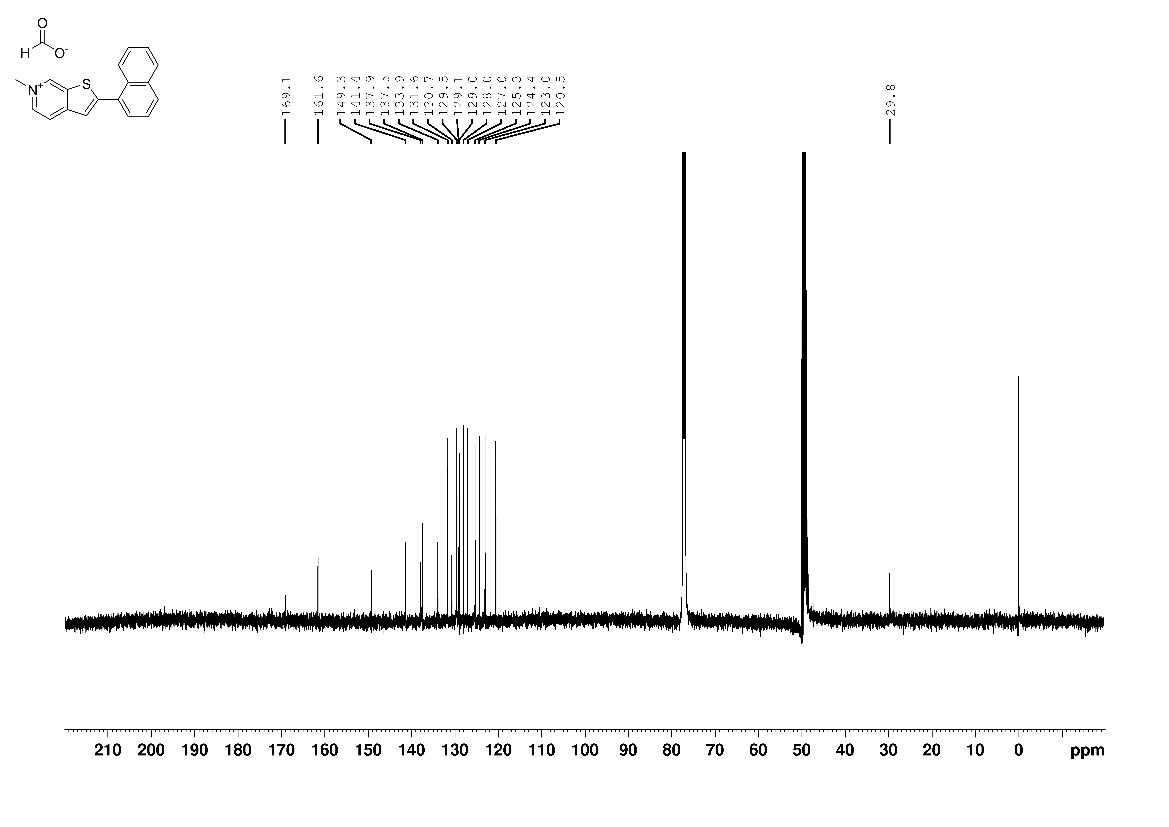


*2-(naphthalen-1-yl)-4,5,6,7-tetrahydrothieno[2,3-c]pyridine (****23****)*

**
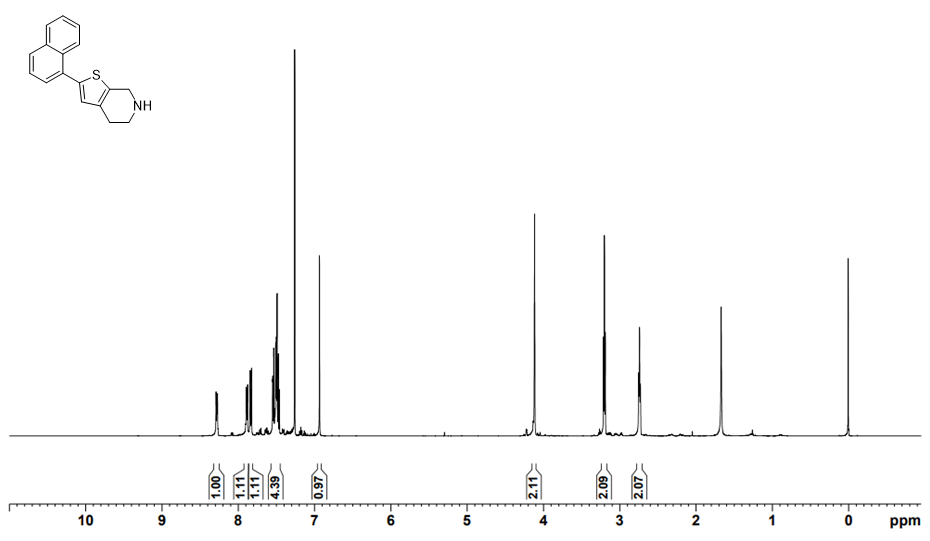
**

**
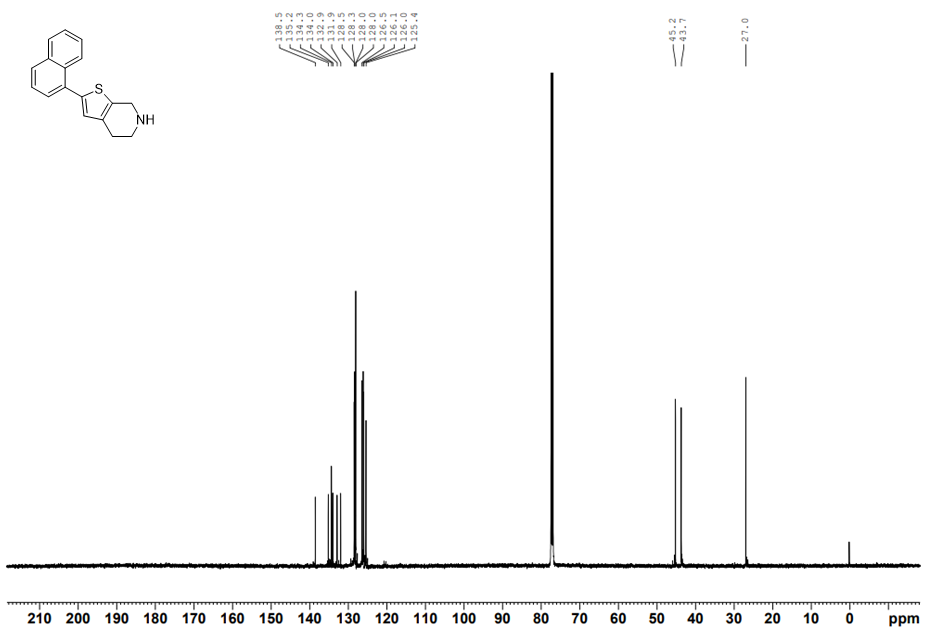
**

*6-methyl-2-(naphthalen-1-yl)-4,5,6,7-tetrahydrothieno[2,3-c]pyridin-6-ium (****24****)*

**
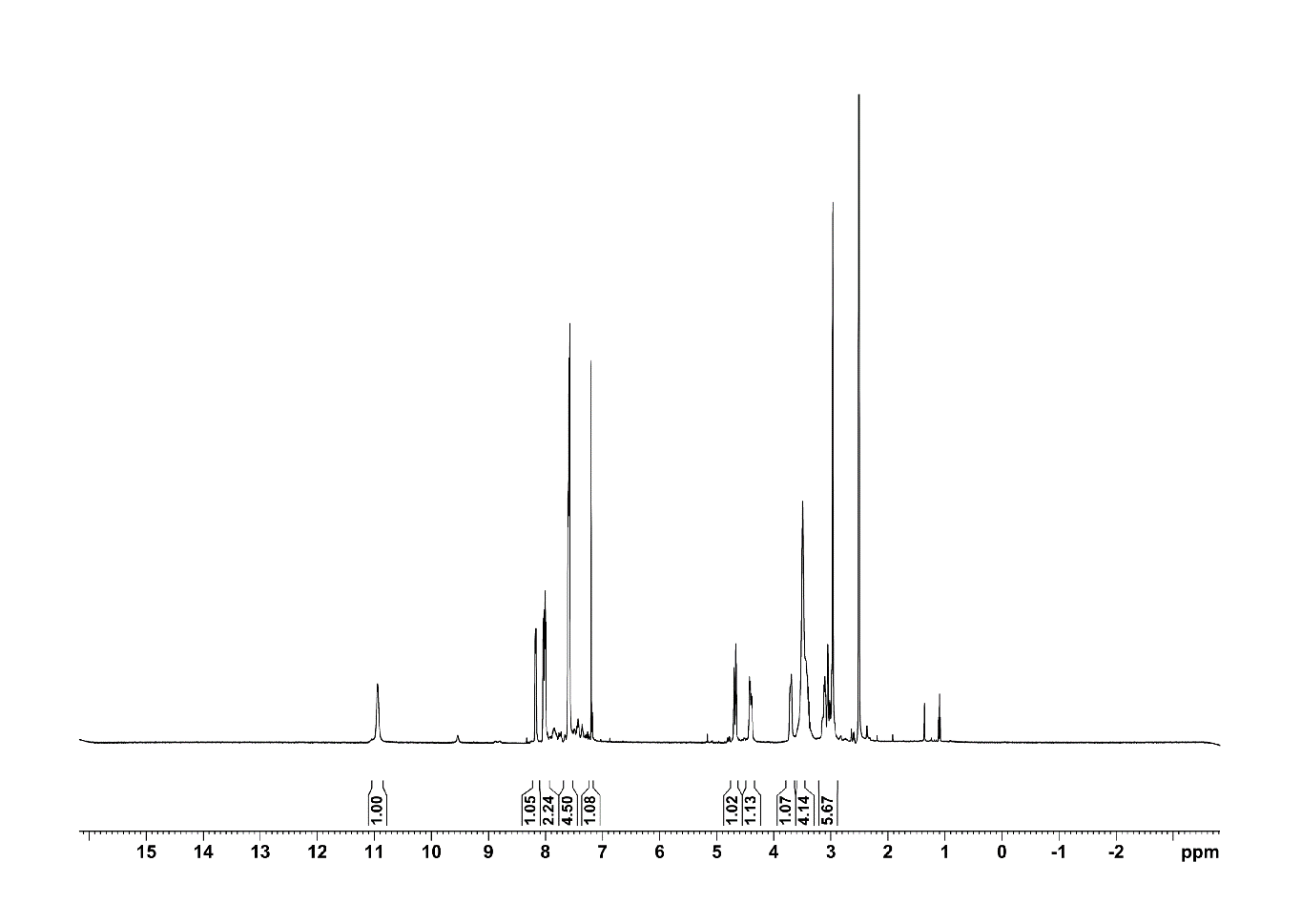
**

**
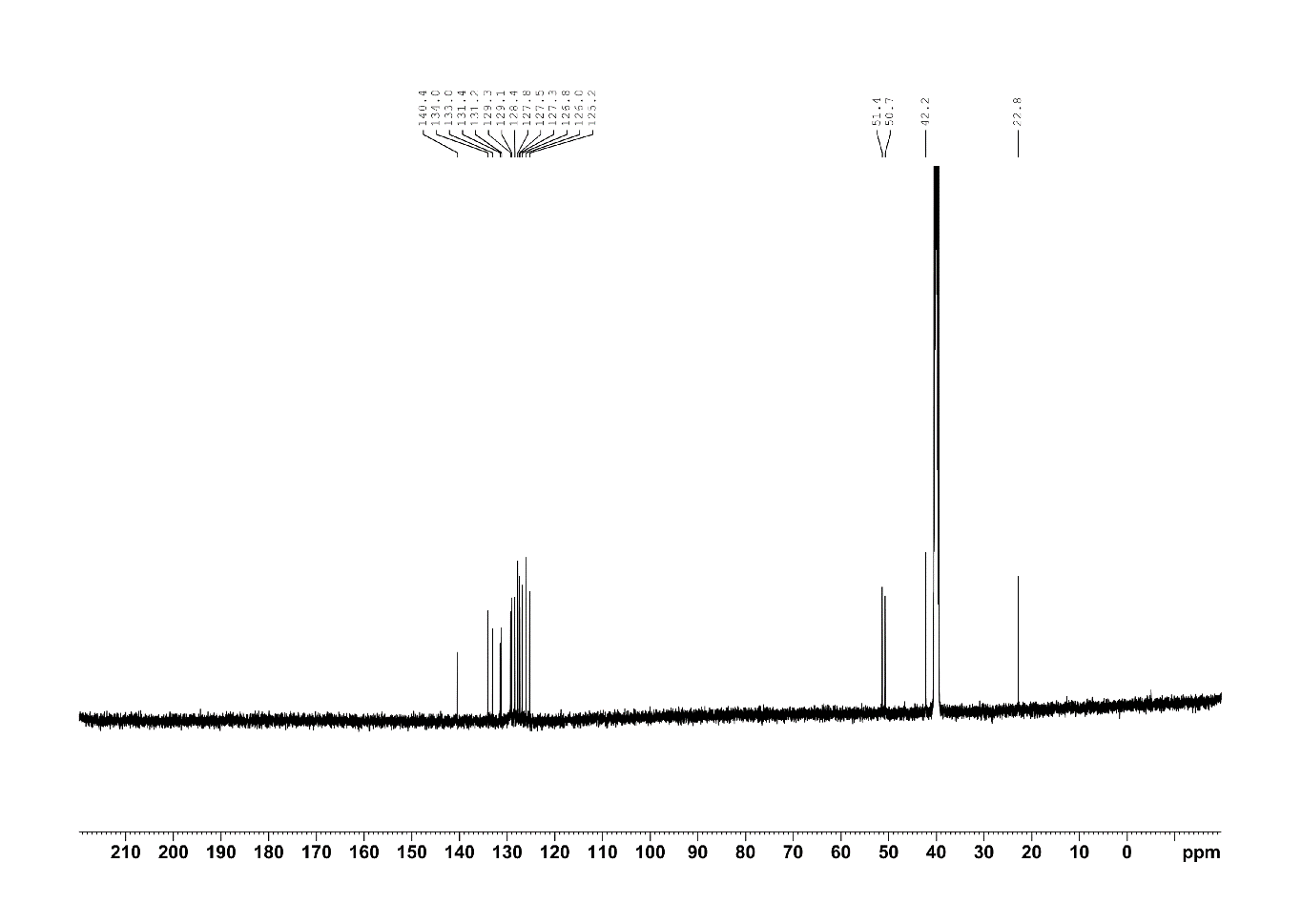
**

### Supporting Table S1 – Molecular docking

**Table S1.** Molecular docking results for selected designed molecules using Glide^a^

| ID | Design | Docking score^a^  (kcal/mol) | | No of similar X-ray poses | No of alter-native poses | Discarded poses |
| --- | --- | --- | --- | --- | --- | --- |
|  |  | Mean | Stdev |  |  |  |
| **1-CoA** short (S) | Ref^b^ | **-9,1** | **0,4** | **7** | **2** | **0** |
| **1-CoA** short (R) | Ref^b^ | **-8,8** | **0,3** | **7** | **2** | **1** |
| **8**^C^ (S) | B | **-8,6** | **0,2** | **9** | **1** | **3** |
| **8**^C^ (R) | B | **-8,3** | **0,3** | **6** | **3** | **10** |
| **(E)-9**^C^ | C | **-9,3** | **0,3** | **9** | **1** | **3** |
| **(Z)-9**^C^ | C | **-8,7** | **0,2** | **9** | **1** | **8** |
| **11**^C^ | D | **-8,4** | **0,1** | **9** | **1** | **21** |
| **21**^C^ | D | **-7,8** | **0,2** | **8** | **2** | **8** |
| ^a^10% av top GlideSP docking poses and their score values are included. ^b^**1-CoA** short was used as reference molecule in the dockings. ^C^Chemical structures are shown in table 2 and table S4. | | | | | | |

### Supporting Table S2 – Designed and synthesized compounds

**Table S2.** Complementary list of designed and synthesised compounds with Apparent IC_50_ values against ChAT, CrAT and AChE.

| Cpd No. | Structure | Apparent IC_50_ (µM)^a, b^ | | | Counter screen | |
| --- | --- | --- | --- | --- | --- | --- |
|  |  | ChAT | CrAT | AChE |  |  |
| Group A | | | | | | |
| 12 | 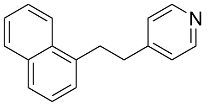 | > 500^c^ | 400^c^  (330-500) | n.d. | n.d. | |
| 13 | 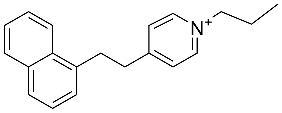 | > 500 | > 500 | n.d. | n.d. | |
| 14 | 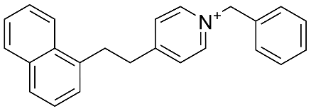 | > 500 | > 500 | 0.61  (0.53-0.7) | n.d. | |
| Group B | | | | | | |
| 15 | 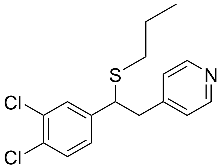 | 500^c^  (370-810) | 270^c^  (180-630) | n.d. | | n.d. |
| Group C | | | | | | |
| (Z)-16 | 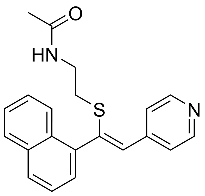 | > 500^c^ | > 500^c^ | > 1000 | | n.d. |
| (Z)-17 | 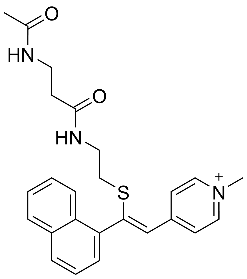 | 250  (160-710) | 260  (210-370) | 11  (9.9-13) | | 240  (120-740) |
| Z-(18) | 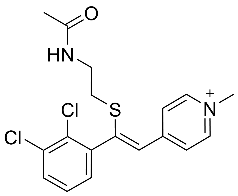 | 520  (320-900) | 350  (150-490) | 24  (17-38) | | 340  (170 -) |
| Z-(19) | 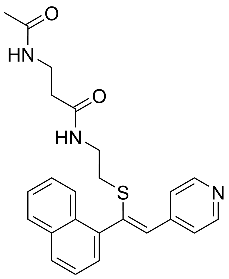 | > 500 | > 500 | n.d. | | n.d. |
| Z-(20) | 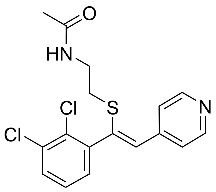 | > 500 | > 500 | > 500 | | n.d. |
| Group D | | | | | | |
| 21 | 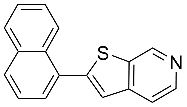 | 160^c^  (120-230) | 110^c^  (80-150) | n.d. | | n.d. |
| 22 | 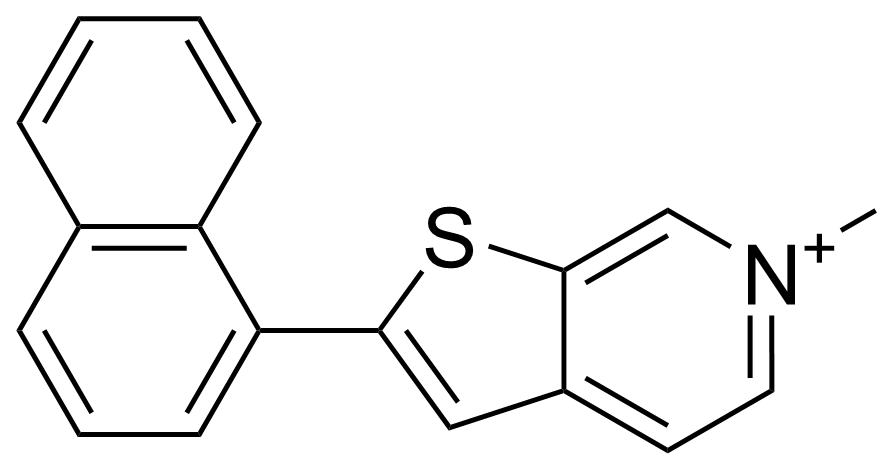 | >500 | 260  (170-410) | n.d. | | > 500 |
| 23 | 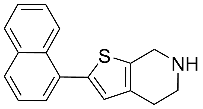 | 650^c^  (540-960) | 420^c^  (350-540) | > 500 | | >500 |
| 24 | 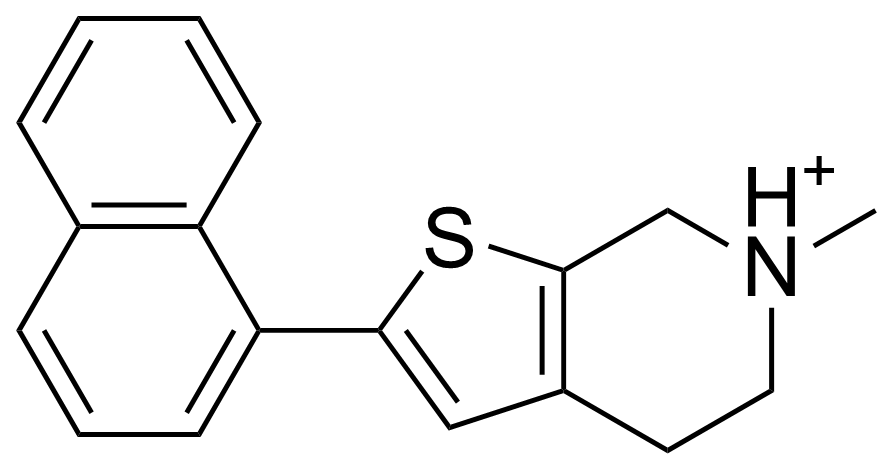 | >500^c^ | 410^c^  (240-730) | n.d. | | n.d. |

^a^ Dose response curves are shown in supporting information Fig. S6

^b^ Mean of at least three replicates. The 95% C.I. is shown in parenthesis

^c^ Ligand precipitated at high concentrations

### Supporting Table S3 – SPR data quality

**Table S3**. Steady state data quality of representative SPR measurements.

| Parameter | CoA | S-ethyl-CoA |
| --- | --- | --- |
| K_D_ (M) | 1.17e-5 | 6.91e-8 |
| R_max_ (RU) | 37.2 | 40.3 |
| Offset (RU) | 0.9 | 0.5 |
| Chi^2^ (RU^2^) | 9.82e-1 (2%) | 2.22e0 (5%) |
| T(K_D_) | 1.04e+01 | 7.78e+0 |
| T(R_max_) | 4.44e+01 | 3.90e+01 |
| T(offset) | 1.79e+0 | 6.55e-01 |

### Supporting Table S4 – X-ray collection and refinement statistics

**Table S4**. Data collection and refinement statistics of **6**•ChAT-SERM, **8**•ChAT-SERM, **(E)-9**•ChAT-SERM och **11**•CrAT.

|  | **(E)-9**•ChAT-SERM | **6**•ChAT-SERM | **8**•ChAT-SERM | **11**•CrAT |
| --- | --- | --- | --- | --- |
| *Data collection^#^* |  |  |  |  |
| PDB ID code | 9RT3 | 9F85 | 9F84 | 9SCK |
| Space group | P2_1_2_1_2_1_ | P2_1_2_1_2_1_ | P2_1_2_1_2_1_ | P2_1_2_1_2_1_ |
| Wavelength (Å) | 0.729 | 0.976 | 0.976 | 0.729 |
| Unit cell dimensions (Å) | 54.8, 76.2, 165 | 54.8, 76.4, 164 | 54.7, 76.8, 164 | 57.9, 84.9, 138 |
| Resolution range (Å) | 165-1.80  (1.84-1.80) | 164-1.6  (1.63-1.6) | 82.2-1.9  (1.94-1.90) | 47.8-1.40 (1.42-1.4) |
| Total no. of reflections | 867736  (52948) | 1240640 (60102) | 479594  (30011) | 1600519 (39385) |
| Unique reflections | 64023 (3805) | 91684 (4471) | 55320 (3496) | 133946 (6467) |
| Completeness (%) | 98.8 (100) | 100 (100) | 99.8 (99.5) | 99.9 (98.5) |
| Multiplicity | 13.6 (13.9) | 13.5 (13.4) | 8.7 (8.6) | 11.9 (6.1) |
| *R*_merge_* | 0.052 (1.24) | 0.054 (0.940) | 0.047 (1.02) | 0.085 (0.648) |
| Mean *I/σ(I)* | 13.9 (2.3) | 13.5 (2.6) | 12.3 (2.3) | 9.0 (2.1) |
| CC_1/2_ | 0.998 (0.643) | 0.998 (0.850) | 0.998 (0.719) | 0.996 (0.714) |
| *Refinement* |  |  |  |  |
| *R* factor/*R*_free_^§^ (%) | 18.3/21.2 | 17.5/20.0 | 16.8/19.7 | 14.5/16.8 |
| Average B value for  protein atoms (Å^2^) | 30.4 | 30.3 | 31.6 | 17.7 |
| Rmsd from ideal values |  |  |  |  |
| Bond lengths (Å) | 0.004 | 0.014 | 0.009 | 0.005 |
| Bond angles (°) | 0.640 | 1.25 | 0.91 | 0.824 |
| Ramachandran (%) |  |  |  |  |
| Favored | 98.3 | 97.5 | 98.1 | 98.3 |
| Allowed | 1.75 | 2.47 | 1.93 | 1.70 |
| Outliers | 0 | 0 | 0 | 0 |

^#^Statistics from Aimless.

**R*_merge_ = (∑|*I - <I>*|)/∑*I*, where *I* is the observed intensity, and *<I>* is the average intensity obtained after multiple observations of symmetry-related reflections.

*R* factor = (∑||*F_o_*| - |*F_c_*|)/∑*F_o_*, where *F_o_* is observed structure factor, and *F_c_* is a calculated structure factor

^§^*R*_free_ uses 2% randomly chosen reflections from a reference data set.

### Supporting Figure S1 – TSA selectivity studies data


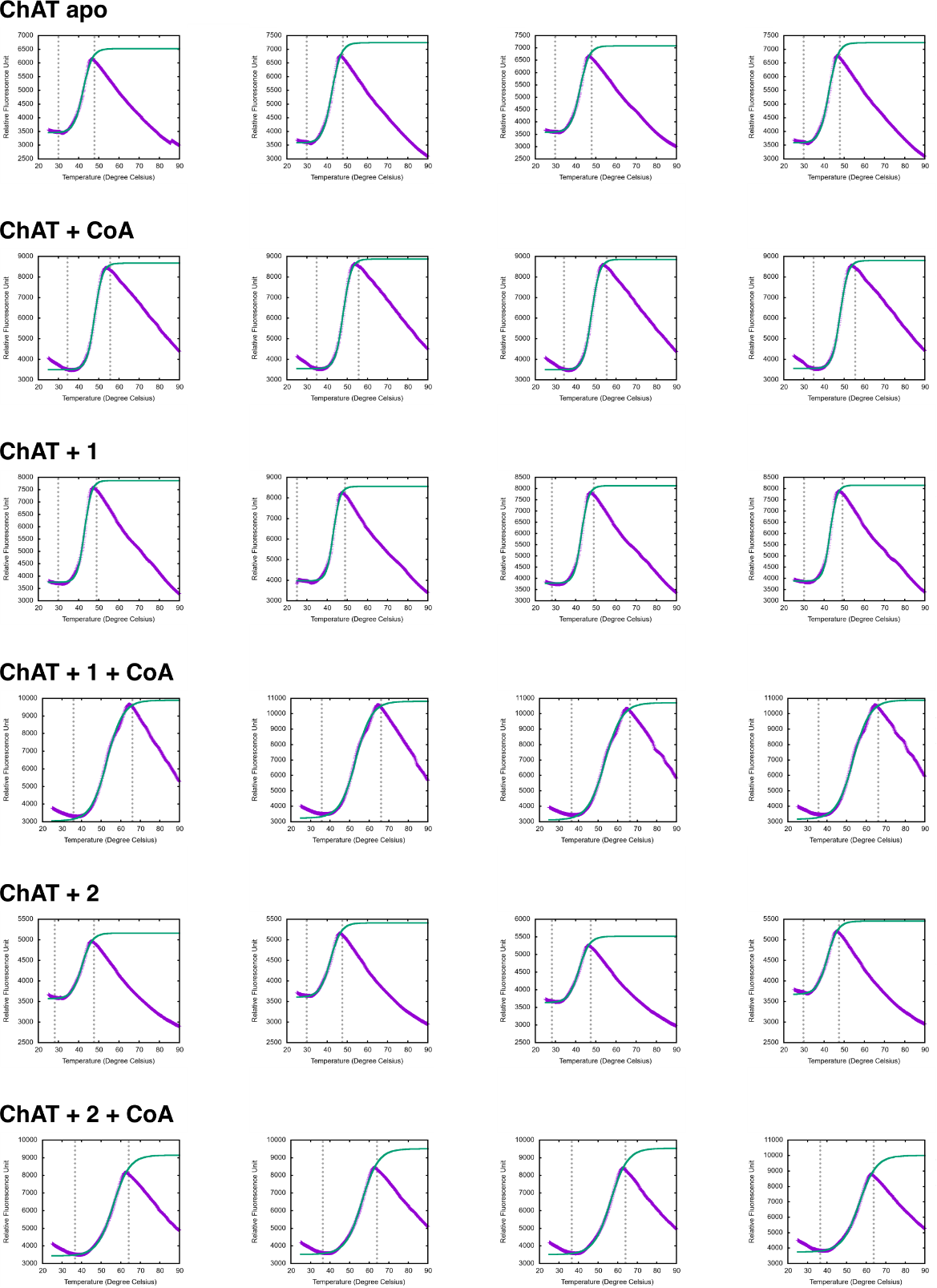


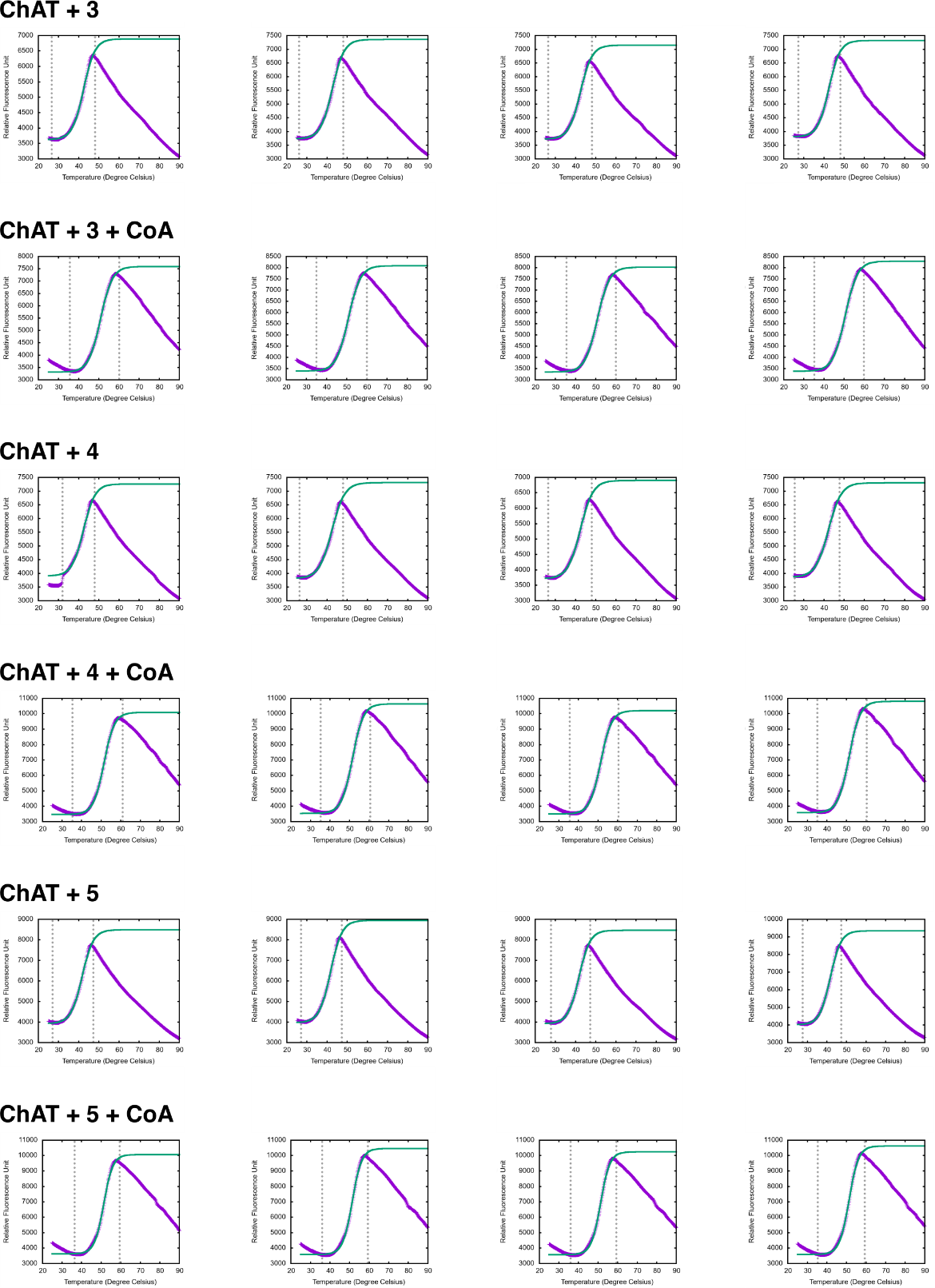


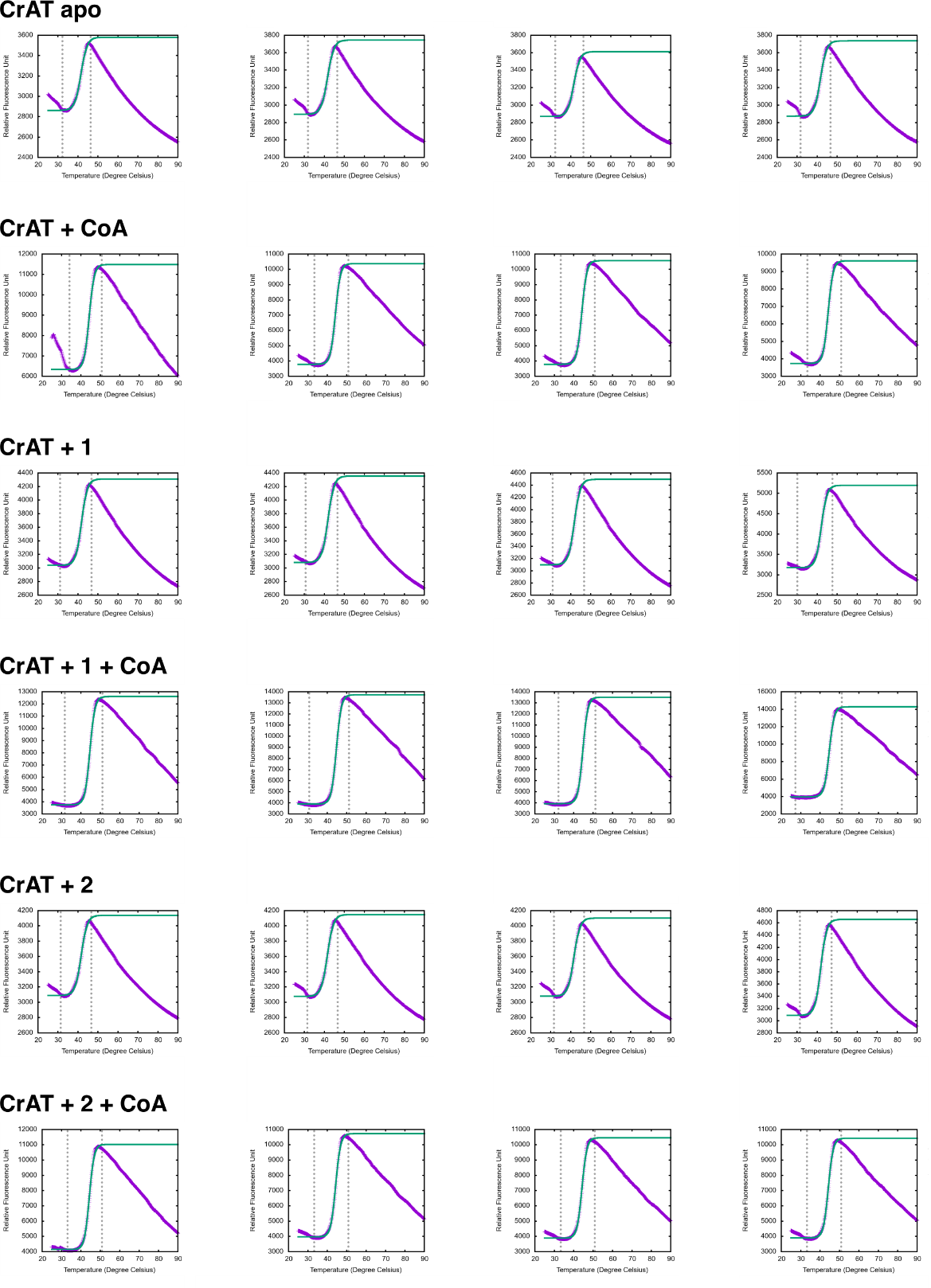


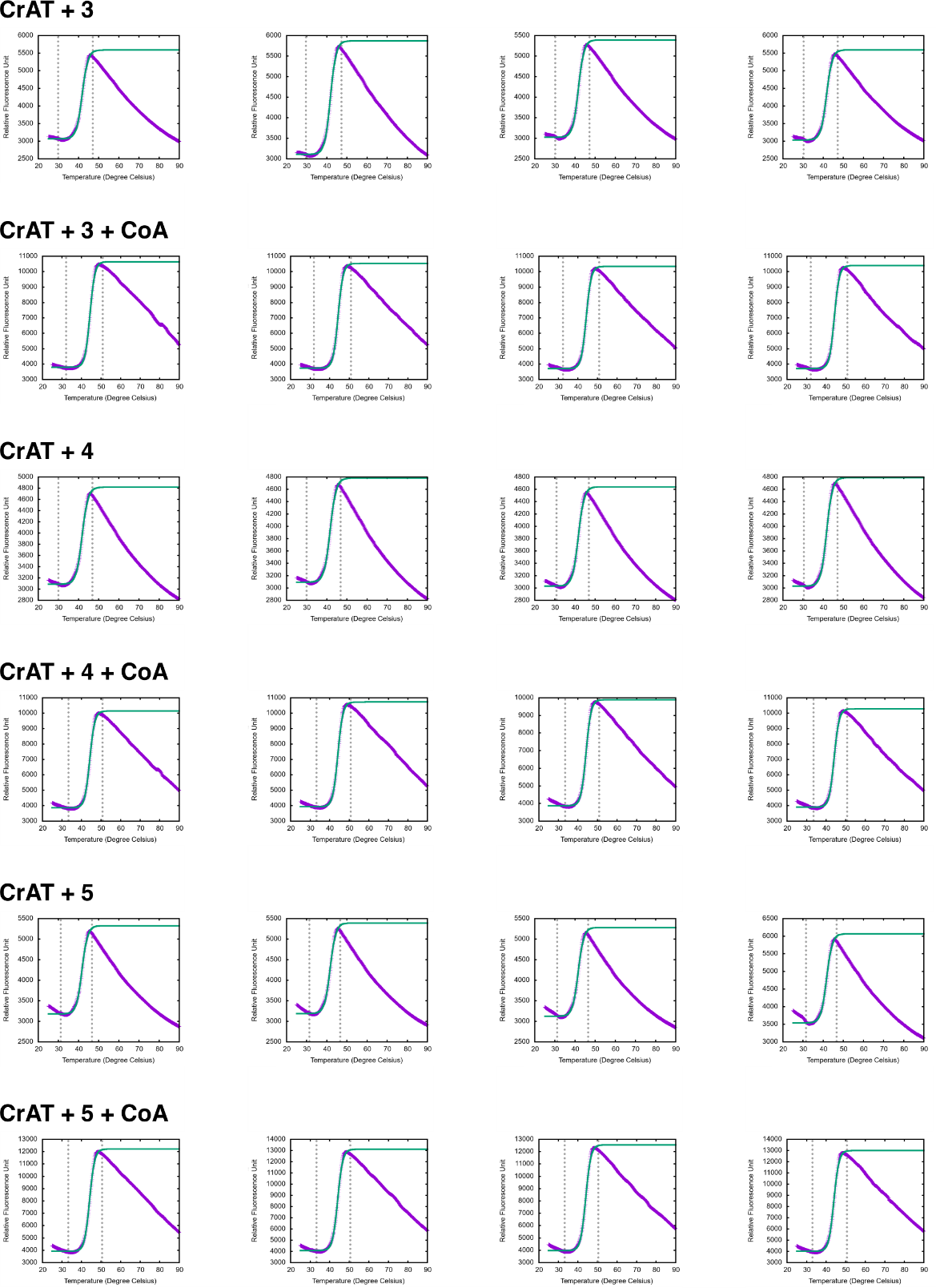


**Fig. S1. Selectivity studies**. TSA raw data shown as solid lines (magenta) and the fit to Boltzmann sigmoidal equation (shown as solid green lines).

### Supporting Figure S2 – Molecular docking


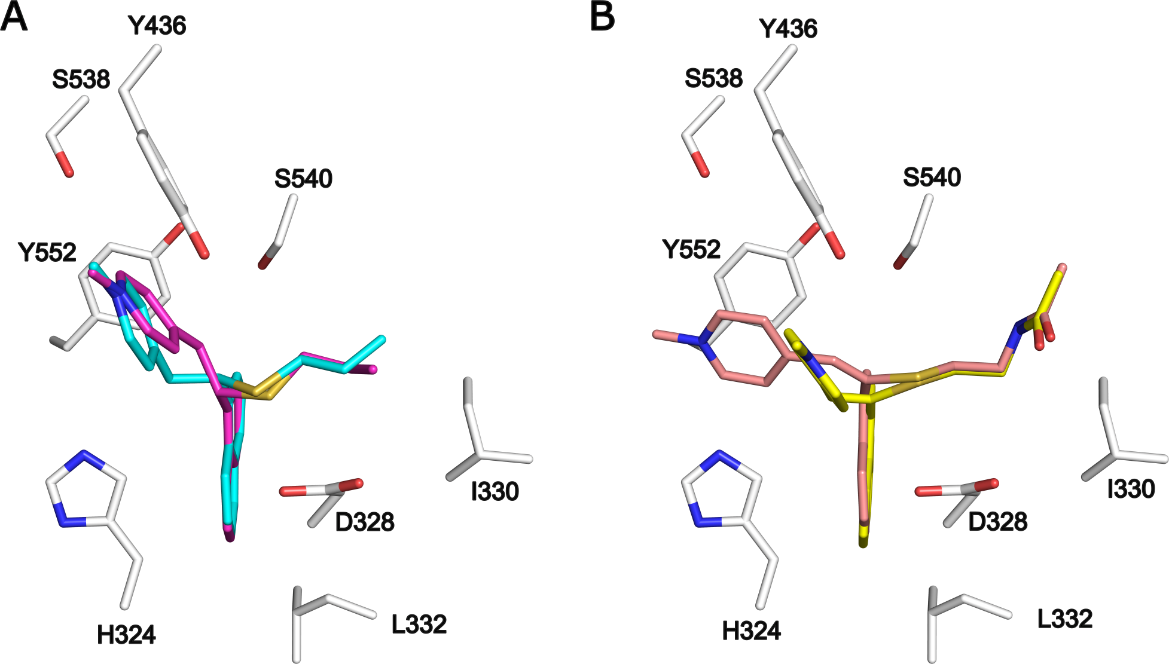


**Fig. S2. Top-ranked docked structures.** (A) Binding of compounds **8 (S)** and **8 (R)** in the active site tunnel of ChAT-SERM; (B) Binding of **(E)-9** and **(Z)-9** in ChAT-SERM. Compounds **8 (S)**, **8 (R)**, **(E)-9**, and **(Z)-9** are shown in magenta, cyan, pink, and yellow, respectively. Oxygen, nitrogen, and sulfur atoms are colored red, blue, and yellow, respectively.

### Supporting Figure S3 – CPM assay

**Fig. S3. Fluorescence of CPM in presence and absence of ChAT and CrAT**. Mean and S.D. (n=4)

### Supporting Figure S4 – UHPLC-HRMS of CPM

1. CPM treated ChAT, sequence coverage 63%

| **1** | HHHHHHDYDI | PTTENLYFQG | AAAAK**TPSSE** | **ESGLPKLPVP** | **PLQQTLATYL** |
| --- | --- | --- | --- | --- | --- |
| **51** | **QCMRHLVSEE** | **QFRKSQAIVQ** | **QFGAPGGLGE** | **TLQQK**LLERQ | EK**TANWVSEY** |
| **101** | **WLNDMYLNNR** | **LALPVNSSPA** | **VIFARQHFPG** | **TDDQLRFAAS** | **LISGVLSYKA** |
| **151** | **LLDSHSIPTD** | **CAKGQLSGQP** | **LCMKQYYGLF** | **SSYR**LPGHTQ | DTLVAQNSSI |
| **201** | MPEPEHVIVA | CCNQFFVLDV | VINFR**RLSEG** | **DLFTQLRK**IV | K**MASNEDERL** |
| **251** | **PPIGLLTSDG** | **RSEWAEAR**TV | LVK**DSTNRDS** | **LDMIERCICL** | **VCLDAPGGVE** |
| **301** | **LSDTHRALQL** | **LHGGGYSK**NG | ANR**WYDKSLQ** | **FVVGR**DGTCG | VVCEHSPFDG |
| **351** | IVLVQCTEHL | LKHMTQSSRK | **LIRADSVSEL** | **PAPR**RLRWK**C** | **SPEIQGHLAS** |
| **401** | **SAEK**LQRIVK | **NLDFIVYKFD** | **NYGK**TFIKKQ | K**CSPDAFIQV** | **ALQLAFYR**LH |
| **451** | R**RLVPTYESA** | **SIRRFQEGRV** | **DNIRSATPEA** | **LAFVR**AVTDH | KAAVPASEKL |
| **501** | LLLKDAIR**AQ** | **TAYTVMAITG** | **MAIDNHLLAL** | **R**ELAR**AMCKE** | **LPEMFMDETY** |
| **551** | **LMSNR**FVLST | SQVPTTTEMF | CCYGPVVPNG | YGACYNPQPE | TILFCISSFH |
| **601** | SCKETSSSKF | AK**AVEESLID** | **MRDLCSLLPP** | **TESKPLATK**E | KATRPSQGHQ |
| **651** | P |  |  |  |  |

1. CPM treated CrAT, sequence coverage 62%

| **1** | MRGSHHHHHH | TDPLPR**LPVP** | **PLQQSLDHYL** | **KALQPIVSEE** | **EWAHTKQLVD** |
| --- | --- | --- | --- | --- | --- |
| **51** | **EFQASGGVGE** | **R**LQKGLERRA | R**KTENWLSEW** | **WLKTAYLQYR** | **QPVVIYSSPG** |
| **101** | **VMLPKQDFVD** | **LQGQLR**FAAK | **LIEGVLDFKV** | **MIDNETLPVE** | **YLGGKPLCMN** |
| **151** | **QYYQILSSCR** | **VPGPKQDTVS** | **NFSK**TKKPPT | HITVVHNYQF | FELDVYHSDG |
| **201** | TPLTADQIFV | QLEK**IWNSSL** | **QTNKEPVGIL** | **TSNHR**NSWAK | **AYNTLIK**DKV |
| **251** | NRDSVRSIQK | **SIFTVCLDAT** | **MPRVSEDVYR** | SHVAGQMLHG | GGSRLNSGNR |
| **301** | WFDK**TLQFIV** | **AEDGSCGLVY** | **EHAAAEGPPI** | **VTLLDYVIEY** | **TK**KPELVR**SP** |
| **351** | **MVPLPMPK**KL | R**FNITPEIK**S | DIEKAK**QNLS** | **IMIQDLDITV** | **MVFHHFGKDF** |
| **401** | **PKSEKLSPDA** | **FIQMALQLAY** | **YRIYGQACAT** | **YESASLR**MFH | LGRTDTIR**SA** |
| **451** | **SMDSLTFVKA** | **MDDSSVTEHQ** | **KVELLR**KAVQ | AHRGYTDRAI | RGEAFDRHLL |
| **501** | GLK**LQAIEDL** | **VSTPDIFMDT** | **SYAIAMHFHL** | **STSQVPAK**TD | CVMFFGPVVP |
| **551** | DGYGVCYNPM | EAHINFSLSA | YNSCAETNAA | RLAHYLEKAL | LDMRALLQSH |
| **601** | PRAK**LISEED** | **LSLISG** |  |  |  |

**Fig. S4. UHPLC-HRMS of CPM treated samples**. ChAT (20 µg) incubated with CPM (240 µM), CrAT (20 µg) incubated with CPM for 2 minutes prior to methanol precipitation, peptidic cleavage and analysis using UHPLC-HRMS. Identified peptides are shown in bold red: (A) The CPM treated ChAT sample had a sequence coverage of 63% and 10 of 20 cysteins were detected. All cysteine residues detected was found to form an adduct with CPM (cyan); (B) The CPM treated CrAT sample had a sequence coverage of 62%, 5 of 8 cysteins was detected and 3 of these were found to form an adduct with CPM (cyan).

### Supporting Figure S5 – UHPLC-HRMS of controls

1. Untreated reference ChAT, sequence coverage 74%

| **1** | HHHHHHDYDI | PTTENLYFQG | AAAAK**TPSSE** | **ESGLPKLPVP** | **PLQQTLATYL** |
| --- | --- | --- | --- | --- | --- |
| **51** | **QCMRHLVSEE** | **QFRKSQAIVQ** | **QFGAPGGLGE** | **TLQQK**LLERQ | EK**TANWVSEY** |
| **101** | **WLNDMYLNNR** | **LALPVNSSPA** | **VIFARQHFPG** | **TDDQLRFAAS** | **LISGVLSYKA** |
| **151** | **LLDSHSIPTD** | **CAKGQLSGQP** | **LCMKQYYGLF** | **SSYRLPGHTQ** | **DTLVAQNSSI** |
| **201** | **MPEPEHVIVA** | **CCNQFFVLDV** | **VINFRRLSEG** | **DLFTQLRK**IV | K**MASNEDERL** |
| **251** | **PPIGLLTSDG** | **RSEWAEAR**TV | LVK**DSTNRDS** | **LDMIERCICL** | **VCLDAPGGVE** |
| **301** | **LSDTHRALQL** | **LHGGGYSK**NG | ANR**WYDKSLQ** | **FVVGRDGTCG** | **VVCEHSPFDG** |
| **351** | **IVLVQCTEHL** | **LK**HMTQSSRK | **LIRADSVSEL** | **PAPR**RLRWK**C** | **SPEIQGHLAS** |
| **401** | **SAEK**LQRIVK | **NLDFIVYKFD** | **NYGK**TFIKK**Q** | **KCSPDAFIQV** | **ALQLAFYR**LH |
| **451** | RR**LVPTYESA** | **SIRRFQEGRV** | **DNIRSATPEA** | **LAFVR**AVTDH | KAAVPASEK**L** |
| **501** | **LLLKDAIRAQ** | **TAYTVMAITG** | **MAIDNHLLAL** | **R**ELARAMCK**E** | **LPEMFMDETY** |
| **551** | **LMSNR**FVLST | SQVPTTTEMF | CCYGPVVPNG | YGACYNPQPE | TILFCISSFH |
| **601** | SCKETSSSKF | AK**AVEESLID** | **MRDLCSLLPP** | **TESKPLATK**E | KATRPSQGHQ |
| **651** | P |  |  |  |  |

1. Untreated reference CrAT, sequence coverage 62%

| **1** | MRGSHHHHHH | TDPLPR**LPVP** | **PLQQSLDHYL** | **KALQPIVSEE** | **EWAHTKQLVD** |
| --- | --- | --- | --- | --- | --- |
| **51** | **EFQASGGVGE** | **RLQK**GLERRA | R**KTENWLSEW** | **WLKTAYLQYR** | **QPVVIYSSPG** |
| **101** | **VMLPKQDFVD** | **LQGQLRFAAK** | **LIEGVLDFK**V | MIDNETLPVE | YLGGKPLCMN |
| **151** | QYYQILSSCR | **VPGPKQDTVS** | **NFSK**TKKPPT | HITVVHNYQF | FELDVYHSDG |
| **201** | TPLTADQIFV | QLEK**IWNSSL** | **QTNKEPVGIL** | **TSNHR**NSWAK | **AYNTLIK**DKV |
| **251** | NRDSVR**SIQK** | **SIFTVCLDAT** | **MPRVSEDVYR** | **SHVAGQMLHG** | **GGSR**LNSGNR |
| **301** | WFDK**TLQFIV** | **AEDGSCGLVY** | **EHAAAEGPPI** | **VTLLDYVIEY** | **TK**KPELVR**SP** |
| **351** | **MVPLPMPK**KL | R**FNITPEIKS** | **DIEK**AK**QNLS** | **IMIQDLDITV** | **MVFHHFGKDF** |
| **401** | **PKSEKLSPDA** | **FIQMALQLAY** | **YRIYGQACAT** | **YESASLR**MFH | LGRTDTIR**SA** |
| **451** | **SMDSLTFVKA** | **MDDSSVTEHQ** | **KVELLR**KAVQ | AHRGYTDRAI | RGEAFDRHLL |
| **501** | GLK**LQAIEDL** | **VSTPDIFMDT** | **SYAIAMHFHL** | **STSQVPAK**TD | CVMFFGPVVP |
| **551** | DGYGVCYNPM | EAHINFSLSA | YNSCAETNAA | RLAHYLEKAL | LDMRALLQSH |
| **601** | PRAK**LISEED** | **LSLISG** |  |  |  |

**Fig. S5. UHPLC-HRMS of control samples**. Untreated reference samples of ChAT and CrAT (20 µg) precipitated 2 min in methanol prior to peptidic cleavage and analysis using UHPLC-HRMS. Identified peptides are shown in bold red: (A) The untreated reference ChAT sample had a sequence coverage of 74% and 14 of 20 cysteins were detected; (B) Untreated reference CrAT had a sequence coverage of 62% and 3 of 8 cysteins was detected.

### Supporting Figure S6 – Dose response curves

**Fig. S6. Dose-response curves for (E)-9 (A), 11 (B), 14 (C), (Z)-17 (D), (Z)-18 (E) and 22 (F) in ChAT, CrAT, and AChE assays**. Apparent inhibitory activity was measured using the CPM assay for ChAT and CrAT and the Ellman assay for AChE. Only compound - protein combinations yielding apparent IC_50_ values ≤ ~ 500 µM are shown. Datasets for compounds that precipitated at any concentration were excluded. ChAT, CrAT, and enzyme-free counter-screen data are plotted as RFU/s (left axis), while AChE activity is shown as mAU/min (right axis). Where indicated, counter-screens were performed in the absence of enzyme and choline, and with AcCoA substituted with CoA to assess interference with the CPM detection chemistry. Error bars represent the standard deviation of triplicate measurements.

### Supporting Figure S7 – SPR of non-binders

**
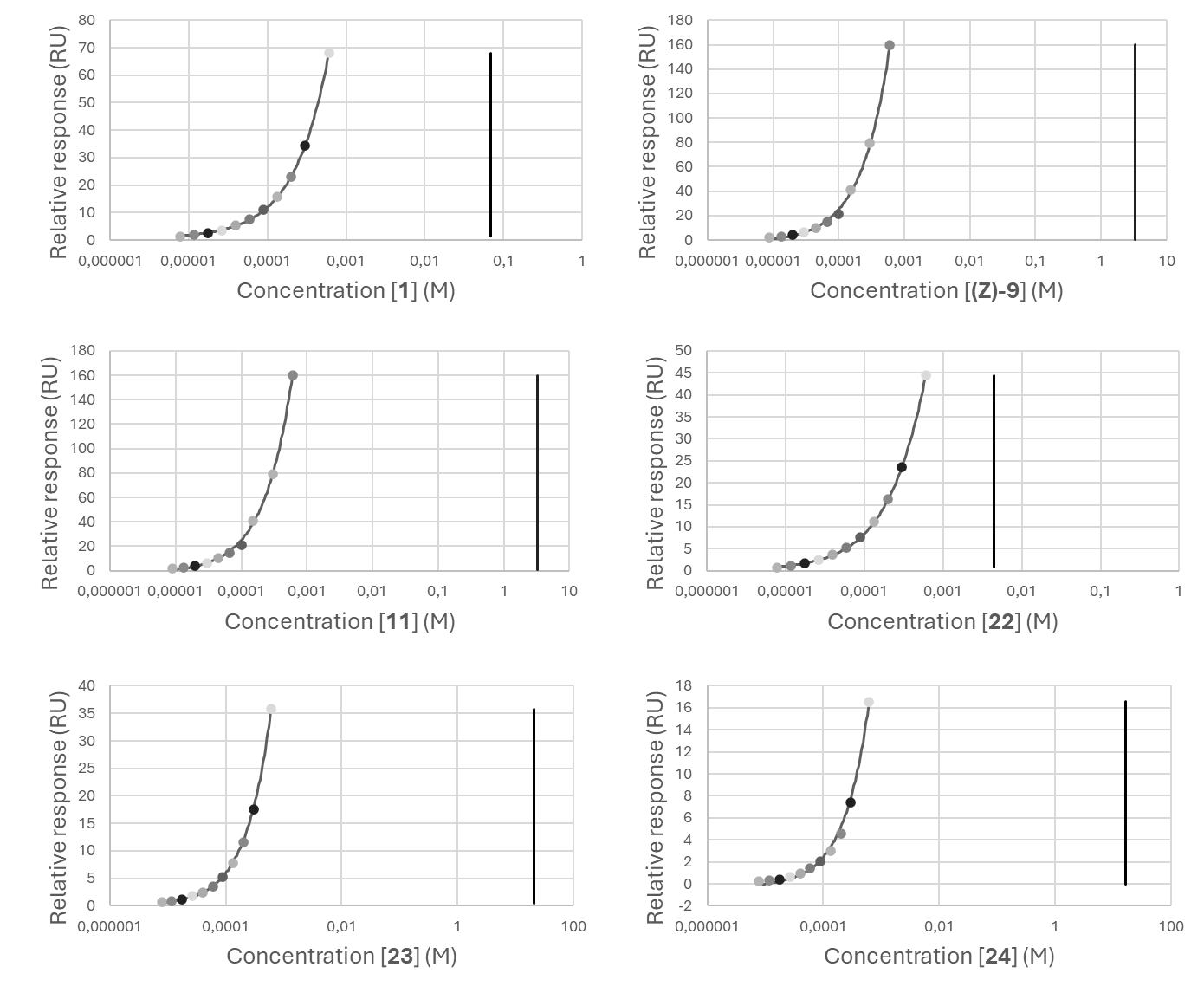
**

**Fig. S7. SPR measurements for non-binding ligands.** Compounds **1**, **(E)-9**, **11**, **22**, **23** and **24** showed no concentration-dependent binding to immobilized ChAT, indicating no detectable reversible interaction within the tested concentration range. SPR measurements of compounds **1**, **(E)-9**, **11**, **22**, **23** and **24** were performed as described in experimental procedures.

### Supporting Figure S8 – Electron density maps


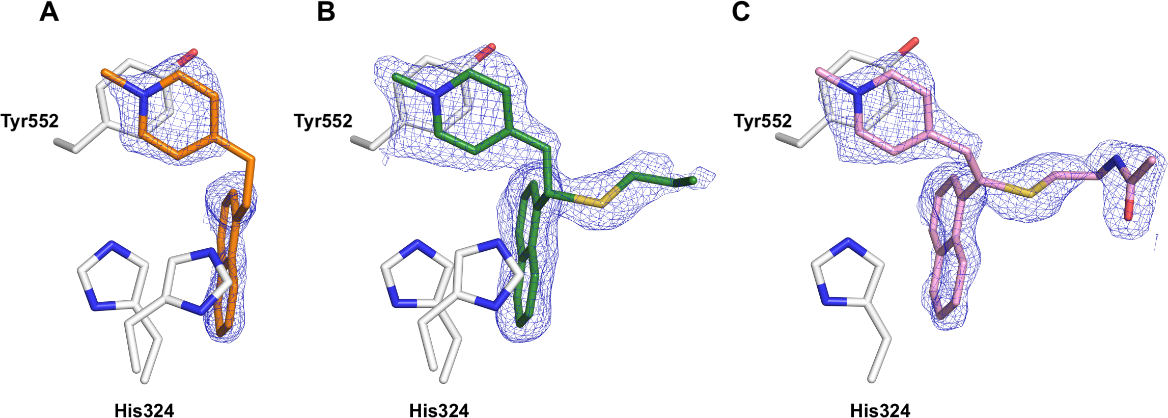


**Fig. S8. Electron density maps.** (A) Complex of **6**•ChAT-SERM; (B) Complex of **8**•ChAT-SERM; (C) Complex of **(E)-9**•ChAT-SERM. The 2F₀-F_c_ omit electron-density maps contoured at 1 σ (blue mesh) are superimposed on the refined models. The ligands are shown in orange, green and pink for **6,** **8** and **(E)-9**, respectively and they were omitted from the refinement to minimise model bias. Protein carbon atoms are grey, nitrogen blue, oxygen red and sulfur yellow.

### Supporting Figure S9 – Structure of **11**•CrAT


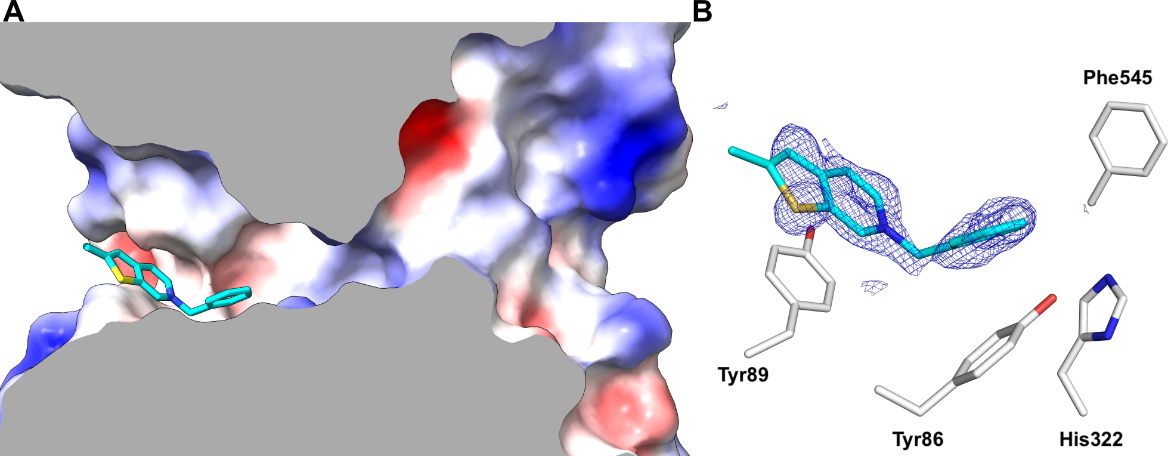


**Fig. S9. X-ray crystal structure of 11•CrAT.** (A) The binding of **11** at the entrance of CrATs vestibule (B) The 2F₀-F_c_ omit electron-density map contoured at 1 σ (blue mesh) superimposed on the refined model. The ligand is shown in cyan and was omitted from the refinement to minimise model bias. Protein carbon atoms are grey, nitrogen blue, oxygen red and sulfur yellow.

### Supporting Figure S10 – UHPLC-HRMS of DTNB

DTNB treated ChAT, sequence coverage 50%

| **1** | HHHHHHDYDI | PTTENLYFQG | AAAAK**TPSSE** | **ESGLPK**LPVP | PLQQTLATYL |
| --- | --- | --- | --- | --- | --- |
| **51** | QCMR**HLVSEE** | **QFRKSQAIVQ** | **QFGAPGGLGE** | **TLQQK**LLER**Q** | **EKTANWVSEY** |
| **101** | **WLNDMYLNNR** | **LALPVNSSPA** | **VIFARQHFPG** | **TDDQLRFAAS** | **LISGVLSYK**A |
| **151** | LLDSHSIPTD | CAK**GQLSGQP** | **LCMKQYYGLF** | **SSYR**LPGHTQ | DTLVAQNSSI |
| **201** | MPEPEHVIVA | CCNQFFVLDV | VINFR**RLSEG** | **DLFTQLRK**IV | K**MASNEDERL** |
| **251** | **PPIGLLTSDG** | **RSEWAEAR**TV | LVK**DSTNRDS** | **LDMIERCICL** | **VCLDAPGGVE** |
| **301** | **LSDTHRALQL** | **LHGGGYSK**NG | ANR**WYDKSLQ** | **FVVGR**DGTCG | VVCEHSPFDG |
| **351** | IVLVQCTEHL | LKHMTQSSRK | **LIRADSVSEL** | **PAPR**RLRWKC | SPEIQGHLAS |
| **401** | SAEKLQRIVK | **NLDFIVYKFD** | **NYGK**TFIKKQ | KCSPDAFIQV | ALQLAFYRLH |
| **451** | R**RLVPTYESA** | **SIRRFQEGRV** | **DNIRSATPEA** | **LAFVR**AVTDH | KAAVPASEKL |
| **501** | LLLKDAIR**AQ** | **TAYTVMAITG** | **MAIDNHLLAL** | **R**ELARAMCK**E** | **LPEMFMDETY** |
| **551** | **LMSNR**FVLST | SQVPTTTEMF | CCYGPVVPNG | YGACYNPQPE | TILFCISSFH |
| **601** | SCKETSSSKF | AK**AVEESLID** | **MR**DLCSLLPP | TESKPLATKE | KATRPSQGHQ |
| **651** | P |  |  |  |  |

**Fig. S10 UHPLC-HRMS of samples treated with DTNB**. ChAT (20 µg) incubated with DTNB (30 mM) for 2 minutes prior to methanol precipitation, peptidic cleavage and analysis using UHPLC-HRMS. Identified peptides are shown in bold red. The DTNB treated ChAT had a sequence coverage of 50% and 4 of 20 cysteins was detected. One cysteine residue that was found to form an adduct with DTNB is highlighted in Cyan.
